# MAPPER: An Open-Source, High-Dimensional Image Analysis Pipeline Unmasks Differential Regulation of *Drosophila* Wing Features

**DOI:** 10.3389/fgene.2022.869719

**Published:** 2022-04-11

**Authors:** Nilay Kumar, Francisco J. Huizar, Keity J. Farfán-Pira, Pavel A. Brodskiy, Dharsan K. Soundarrajan, Marcos Nahmad, Jeremiah J. Zartman

**Affiliations:** ^1^ Department of Chemical and Biomolecular Engineering, University of Notre Dame, Notre Dame, IN, United States; ^2^ Department of Physiology, Biophysics, and Neurosciences, Center for Research and Advanced Studies of the National Polytechnical Institute (Cinvestav), Mexico City, Mexico

**Keywords:** machine and deep learning, genotype-to-phenotype, statistical approaches for phenomics, feature selection, high-dimensional, complementary genomic tools, developmental biology, systems bioengineering

## Abstract

Phenomics requires quantification of large volumes of image data, necessitating high throughput image processing approaches. Existing image processing pipelines for *Drosophila* wings, a powerful genetic model for studying the underlying genetics for a broad range of cellular and developmental processes, are limited in speed, precision, and functional versatility. To expand on the utility of the wing as a phenotypic screening system, we developed MAPPER, an automated machine learning-based pipeline that quantifies high-dimensional phenotypic signatures, with each dimension quantifying a unique morphological feature of the *Drosophila* wing. MAPPER magnifies the power of *Drosophila* phenomics by rapidly quantifying subtle phenotypic differences in sample populations. We benchmarked MAPPER’s accuracy and precision in replicating manual measurements to demonstrate its widespread utility. The morphological features extracted using MAPPER reveal variable sexual dimorphism across *Drosophila* species and unique underlying sex-specific differences in morphogen signaling in male and female wings. Moreover, the length of the proximal-distal axis across the species and sexes shows a conserved scaling relationship with respect to the wing size. In sum, MAPPER is an open-source tool for rapid, high-dimensional analysis of large imaging datasets. These high-content phenomic capabilities enable rigorous and systematic identification of genotype-to-phenotype relationships in a broad range of screening and drug testing applications and amplify the potential power of multimodal genomic approaches.

## Introduction

### The Challenge of Phenomics in Multicellular Organs

The architectural maxim of L. Sullivan “form follows function” is rigorously observed in many biological structures where shape is a key determinant of function (Sullivan, 1896). Mapping the functional relationships between genotypes and phenotypes involves translating phenotypic data, typically available as an image, into a high-dimensional space that describes key morphometric features. The quantification and subsequent comparison of morphometric features is crucial for identifying and explaining gene conditions responsible for the phenotype. Advances in imaging and machine learning (ML) empower the application of phenomics in a high throughput fashion due to the ease of identification of patterns in features (Houle et al., 2017).

**FIGURE 1:**
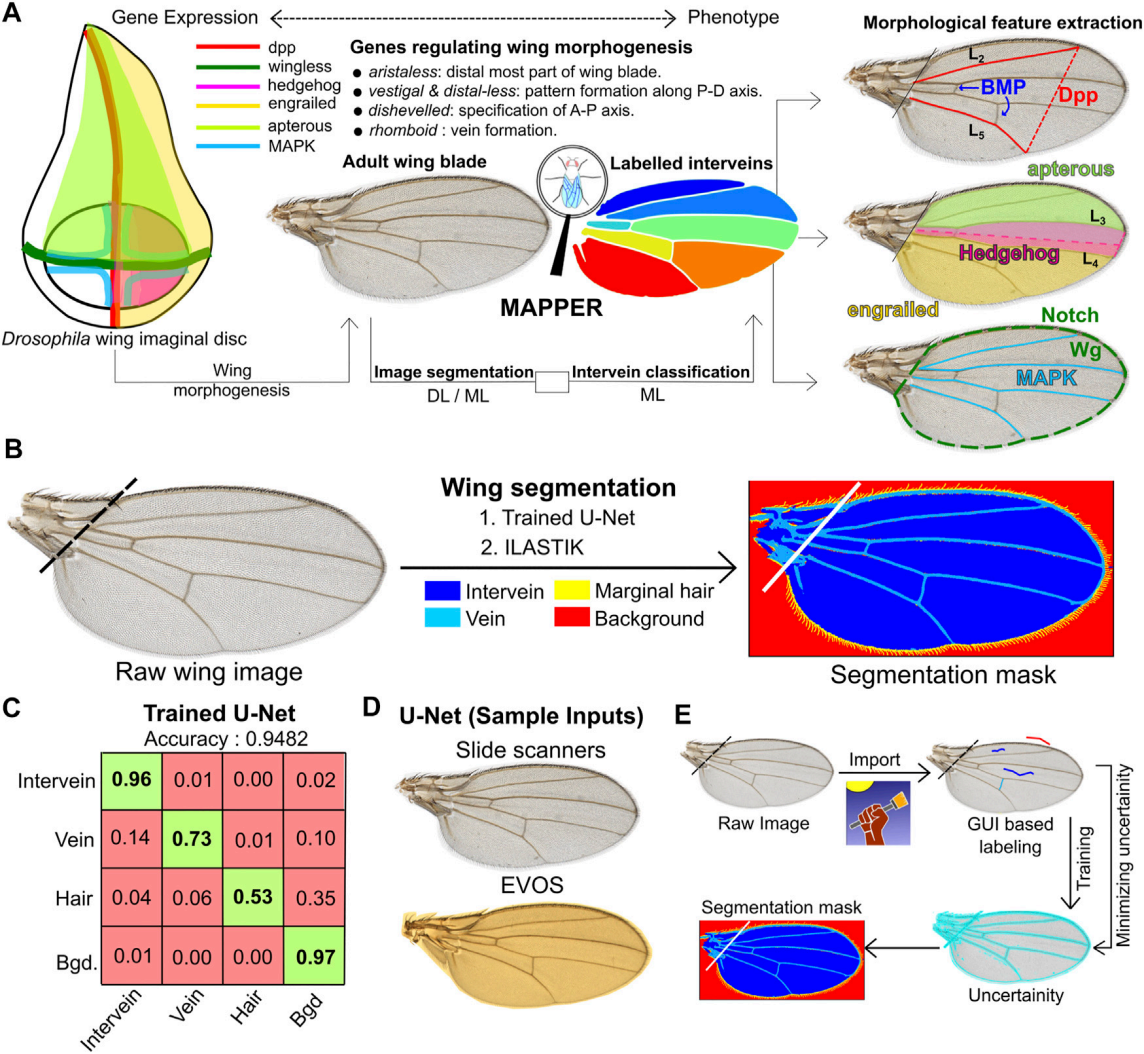
MAPPER automates segmentation of wings. **(A)** Overview of the MAPPER pipeline. The wing imaginal disc development is regulated through the spatiotemporal patterning of multiple classes of genes (Williams et al., 1991; Campbell et al., 1993; Gómez-Skarmeta et al., 1996; Boutros and Mlodzik, 1999). During pupal stages, the wing blade is formed from the wing disc. The coupled image segmentation and intervein classification processes of MAPPER enables morphological feature extraction. **(B)** Segmentation of wings (left) is carried out for identification and labelling of different regions of interest (right) within the adult wing blade. Two methods of training are available: 1 The trained U-Net or 2 ILASTIK. **(C)** MAPPER utilizes the U-Net architecture, which consists of convolutional layers for feature extraction, followed by deconvolution layers to achieve pixel-level predictions. The confusion matrix shows high pixel classification accuracy for a U-Net trained to identify different regions of interest. The numbers in the boxes represent the prediction accuracy for classification of a pixel into a class represented in the vertical labels against the true class in the horizontal labels. **(D)** Sample wings from multiple imaging sources that can be processed by MAPPER. **(E)** Schematic describing methodology followed by ILASTIK, an open source pixel classifier, for the purpose of pixel classification.

The *Drosophila* wing has an excellent track record for genetic screening studies and is ideal for phenomic studies to uncover conserved biological processes relevant to human development and diseases (Pitchers et al., 2019). The *Drosophila* wing has successfully identified genes crucial for organ development and relevant to human health (Strigini and Cohen, 1999; Bier, 2005; Buchmann et al., 2014; Restrepo et al., 2014; Brock et al., 2017; Narciso and Zartman, 2018; Kim et al., 2020). Further, the developing wing imaginal disc has often been used for studying growth, development, and tissue regeneration (Smith-Bolton et al., 2009; Jaszczak and Halme, 2016; Hariharan and Serras, 2017). Thus, the wing is an ideal model system for genotype-phenotype studies due to its balance between structural simplicity and functional complexity. Subtle changes in the shape and size of the wing can provide insights into conserved signaling mechanisms that occur during wing development (Gibson and Dworkin, 2004; Kawecki et al., 2012; Matamoro-Vidal et al., 2018). This unique characteristic of the *Drosophila* wing has enabled completion of a multivariate genome-wide association analysis linking single nucleotide polymorphisms from genotypes to wing shape deformations induced by gene knockdown (Pitchers et al., 2019). The *Drosophila* wing blade consists of five longitudinal veins, two cross veins, intervein trichomes, and marginal hairs along the surface and edge of the wing. These visual features provide a flat readout of conserved signaling pathway activity (Figure 1A; Supplementary Figure S1) (Bier, 2005). Wing development is a systems-level process that requires coordinated regulation of cellular processes such as proliferation, differentiation, and morphogenesis (De Celis, 2003; O’Connor et al., 2006; Neto-Silva et al., 2009; Restrepo et al., 2014; Diaz de la Loza and Thompson, 2017; Huizar et al., 2020). The final shape and size of the adult wing depends on the integration of both intrinsic genetic regulatory networks and extrinsic environmental cues such as temperature, nutrition, and hormones (Johnston and Gallant, 2002; Parker and Struhl, 2020).

Most of the phenotypic studies of wings result in large volumes of imaging data that are not fully utilized. Such data traditionally has been analyzed manually or aided by semi-automated pipelines. Manual extraction of key morphometric features, such as wing size, interveinal areas, shape, trichome (cell number) number and trichome distribution, is impractical over the large sample sizes required to obtain reproducible results. Previous efforts have developed algorithms to perform high throughput analysis of a subset of these features (Houle et al., 2003; Dobens and Dobens, 2013). However, they are still limited by a lack of computational speed, accuracy, and flexibility for various imaging arrangements with respect to the quantification of morphometric traits. Further, existing pipelines only extract a limited number of morphometric traits that provide an incomplete picture of biological implications imposed during experiments.

### High-Dimensional Features Provided by MAPPER

To overcome the limitations of manual and semi-automated platforms, we developed the Multicellular Analysis Processing Platform for Experimental Research (MAPPER), a fully automated pipeline for *Drosophila* wing segmentation and morphometric feature extraction. MAPPER is composed of two distinct modules that operate sequentially. The first module employs a deep learning (DL)-based image segmentation platform to separate wing interveins and veins from the imaging background. This is achieved using the concepts of transfer learning, where we trained the weights of the last few layers of a previously trained convolutional neural network (CNN), U-Net (Ronneberger et al., 2015). The trained DL model generates segmentation masks that define different regions of the wing, at a much faster rate compared to conventional image segmentation algorithms such as active contours or image thresholding. This model can also be re-trained with new images, easily making it more generalizable for datasets belonging to different imaging sources, thereby allowing versatility across various research labs. A second option allows users to employ ILASTIK, a ML-based pixel classifier for the same task.

Following the image segmentation pipeline, is a k-nearest neighbor (KNN)-based machine learning classifier (Cover and Hart, 1967) that classifies and labels each intervein region. This facilitates high throughput feature extraction of each intervein subregions resulting in the extraction of hundreds of geometrical features. Together, these methods allow MAPPER to accurately and swiftly extract large amounts of phenotypic data from wing imaging datasets. MAPPER extracts Elliptic Fourier Descriptors (EFDs) to describe the shape of the wing (Kuhl and Giardina, 1982). EFDs measure local and global changes in the overall shape of wing. The labeling of interveins also provides an orientation-free classification of veins. The pipeline then estimates landmark features and anatomical axes lengths, such as the proximal-distal (PD) axis and the anterior-posterior (AP) axis.

### Case Studies Demonstrating the Capability, Versatility, Accuracy, and Implementation of MAPPER

To benchmark MAPPER’s accuracy and precision in replicating manual measurements, we compared MAPPER’s output to manual measurements of *Drosophila* wings and demonstrate MAPPER measurements are statistically identical to manual measurements. From these measurements, MAPPER was able to reveal scaling relationship differences between males and females from the Samarkand strain of *Drosophila melanogaster*. Further, to compare MAPPER with previous wing analysis packages, we used MAPPER to confirm the role of insulin receptors (InsR) in regulating *Drosophila* wings (Brogiolo et al., 2001). Additionally, MAPPER enabled a complete systematic analysis of how wing shape varies across four *Drosophila* species: *D. ananassae*, *D. melanogaster*, *D. simulans*, and *D. virilis.* MAPPER’s measurements revealed subtle differences, such as the scaling relationships between intervein regions, that would be very difficult to identify from manual or semi-automated platforms. These observations shed light on the genetic regulatory processes that regulate wing shape and size of various experimental conditions. MAPPER is available as an open-source tool in the form of an interactive GUI, making the tool usable and extensible to researchers with no prior experience in programming.

## Results: Pipeline Development, Features, and Usage

### MAPPER Utilizes Statistical Learning Algorithms to Automate Segmentation of *Drosophila* Wing Images

Automation of any quantitative feature extraction pipeline depends primarily on the accuracy of segmentation masks. These masks are used for defining regions of interest within an image. Many regions of interest exist within wings including the intervein regions, the longitudinal veins, and the marginal hairs. Conventional image processing algorithms face a challenge in accurate processing of wing images that might be obtained from variable imaging conditions, such as changes in background lighting or wing rotation. Packages such as WINGMACHINE rely on image thresholding, where parameters need to be recalibrated for separate datasets. In our hands, the WINGMACHINE pipeline required a specific wing orientation for extraction of landmark positions to a pre-fit spline model. WINGMACHINE requires new spline models for wing conditions that result in landmark region abnormalities, such as missing or partial anterior or posterior cross veins. The semi-automated wing analysis platform, FIJIWings, uses the trainable Weka segmentation module to identify these regions (Dobens and Dobens, 2013; Arganda-Carreras et al., 2017). However, manual training is time consuming and needs to be repeated when using images from very different imaging sources. There has been recent work assessing wing phenotypes using an open source ML-based pixel classifier ILASTIK (Sommer et al., 2011) for the task of segmenting the overall wing blade (Alba et al., 2020). However, to date, there is not a fully automated and high throughput image analysis pipeline that can be used for processing a broad range of phenotypes (*e.g.*, severe vein defects and wing deformations) or imaging conditions.

MAPPER provides for flexible training. For larger, high resolution images or very high sample sizes (10^3^) we utilized a CNN, which served as a segmentation algorithm that can be adapted to new identification problems (Kim, 2016). In particular, we retrained the last few neural network layers of a pre-trained U-Net model (Ronneberger et al., 2015; Zhou et al., 2018; Falk et al., 2019), which is a DL-based image segmentation pipeline for identifying different regions of interest. U-Net relies on data augmentation for efficient use of annotated samples. Here, we used a batch size of approximately 1,000 *Drosophila* wings as the initial training dataset.

This training process annotates four different regions within an image. These regional classes are the non-wing background, the intervein regions, the veins, and the periphery hairs (Figure 1B). Wing images that contain sample preparation defects, such as mounting defects or torn wings, are excluded from image analysis (Supplementary Figure S2). Training U-Net through PyTorch using a GPU (Ketkar, 2017) resulted in a deployable model with an overall accuracy of 95% (Figure 1C). The default U-Net model was trained to be compatible for images either taken using a medical slide scanner or an EVOS microscope at a magnification of 4× or higher (Figure 1D).

As a second method that is ideal for low resolution or images with low sample sizes, we used the open source ML-based pixel classifier ILASTIK to generate segmentation masks (Sommer et al., 2011). The ILASTIK toolkit extracted 37 features for each color channel within each pixel. These features included intensity, edge-detection, and texture features. Following the extraction step, a random forest classifier from sci-kit learn was used to obtain a consensus classification for each pixel (Pedregosa et al., 2011). When training MAPPER, ground truth images are added iteratively to reduce the calculated uncertainty of each pixel until the calculated uncertainty reaches a minimum threshold desired by the user (Figure 1E). This segmentation mask is then imported into the custom pipeline of MAPPER for high throughput morphometric quantification of features. Full details on training the image segmentation pipeline for each training method are provided in Supplementary File S1 Section S1.

### MAPPER Provides High-Dimensional Morphological Features Analysis

A key feature of MAPPER is the classification of individual intervein regions. This is carried out by training a ML-based intervein classifier that takes unlabeled intervein regions from the segmentation mask as an input and classifies them according to their location (Supplementary Figure S1). MAPPER then identifies individual veins, intervein regions, and extracts wing shape features (Supplementary File S1 Section S3). The size and positioning of intervein boundaries provides a readout of multiple conserved signaling pathways (Figure 1A). The systematic labeling of interveins also allows construction of quantified phenomes that can establish geometric similarities and dissimilarities between disparate wing samples.

**FIGURE 2:**
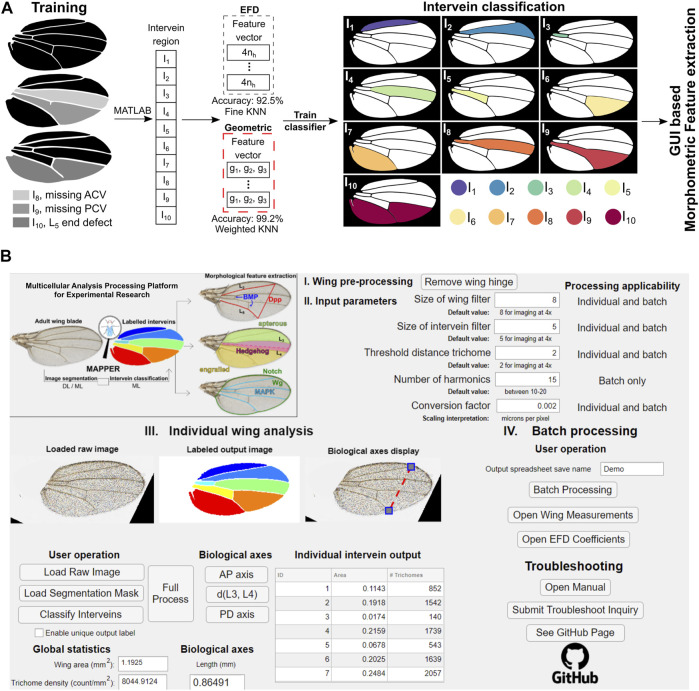
MAPPER automates classification and extraction of a high (>100) dimensional morphological feature set. **(A)** During training, individual intervein regions were manually labelled using the MATLAB’s image labeler app. EFD features along with the geometric features were extracted from the labelled intervein regions to train a machine learning (ML)-based classifier. This trained model then identifies the intervein regions based on the input binary mask and the associated features of each intervein region. Edge cases of anterior cross vein (ACV), posterior cross vein (PCV), and marginal L_5_ defects were included in the analysis. **(B)** Image of the MAPPER application interface. The MAPPER application supports both individual (III) and batch (IV) processing of image data sets based on input parameters (II) specific to a user’s imaging setup.

Segmentation masks generated either by U-Net or ILASTIK are imported into a custom MATLAB pipeline that performs erosion/dilation operations, smoothens the edges, and identifies continuous intervein regions. For training a ML-based intervein classifier, morphological features were first extracted for each manually labelled intervein (Figure 2A). EFD-based shape descriptors were first extracted for each intervein to train the classifier (Kuhl and Giardina, 1982). The key advantage of using such a framework is that EFDs produce a robust, translational and rotation invariant representation of the intervein shape (Kuhl and Giardina, 1982).

EFDs are determined by fitting a Fourier series to the periodic function obtained from the closed *Drosophila* intervein region contour (Supplementary Figure S3). The accuracy of an EFD fit varies with the number of harmonics used in the expansion. We fit EFDs to the seven intervein regions of a wildtype wing to estimate the appropriate number of coefficients required for an accurate representation of shape. The error between the actual contour and the shape approximated by the EFD decreased as the number of terms in the EFD increased (Supplementary Figure S3B). The first ten terms of the EFD were selected for representing the shape of each intervein.

In addition to the EFDs, we extracted basic geometrical properties of the intervein regions including: the ratio of an individual intervein area with respect to the area of the whole wing, the circularity of the region of interest (ROI), and the aspect ratio of each intervein region. Both extracted EFD coefficients and the geometric features of individual interveins were used to train separate models to classify interveins (Figure 2A). This prevents overfitting and selects the set of features that can be used best to classify the interveins. We found that a KNN-based classifier offers the best cross-validation accuracy of the eleven different support vector machine (SVM) and KNN classification methods tested (Supplementary Figure S4C). Overall, the KNN classifier reported an accuracy of about 92.5% when trained on EFD-based features and 99.2% when trained on the geometric features of each intervein (provided in the confusion matrix of Supplementary Figure S4D).

Based on this, we used an intervein classification scheme based on geometric features for classification of interveins to analyze images (Figure 2A). In summary, for any new segmentation mask, the geometric features described above are extracted from each intervein. The features are then passed into the trained intervein classification model that classifies and labels each intervein region.

The labelling of interveins is followed by a series of operations to extract morphological features from the wing blade (Supplementary File S1 Section S3). The approach also systematically extracts localized geometric features that can be used for phenomic analysis. One of the key features extracted using MAPPER are the EFD coefficients for the wing periphery (Supplementary File S1 Section S3D, Supplementary Figure S5). For this particular step, EFDs were not normalized against size for quantification of changes in area of the wing. To do so, we modified the original algorithm such that the EFDs produced are sensitive to size changes. This is accomplished by removing the normalization step (Thomas, 2020) where the EFD coefficients are normalized by the semi-major axis of the first ellipse (Supplementary File S1 Section S3). Altogether, coefficients of the Fourier series are included as additional features, each of them carrying a local shape property. These coefficients can not only be used for screening local shape changes within the wing, but also can be used to estimate an average shape for a particular genotype.

**FIGURE 3:**
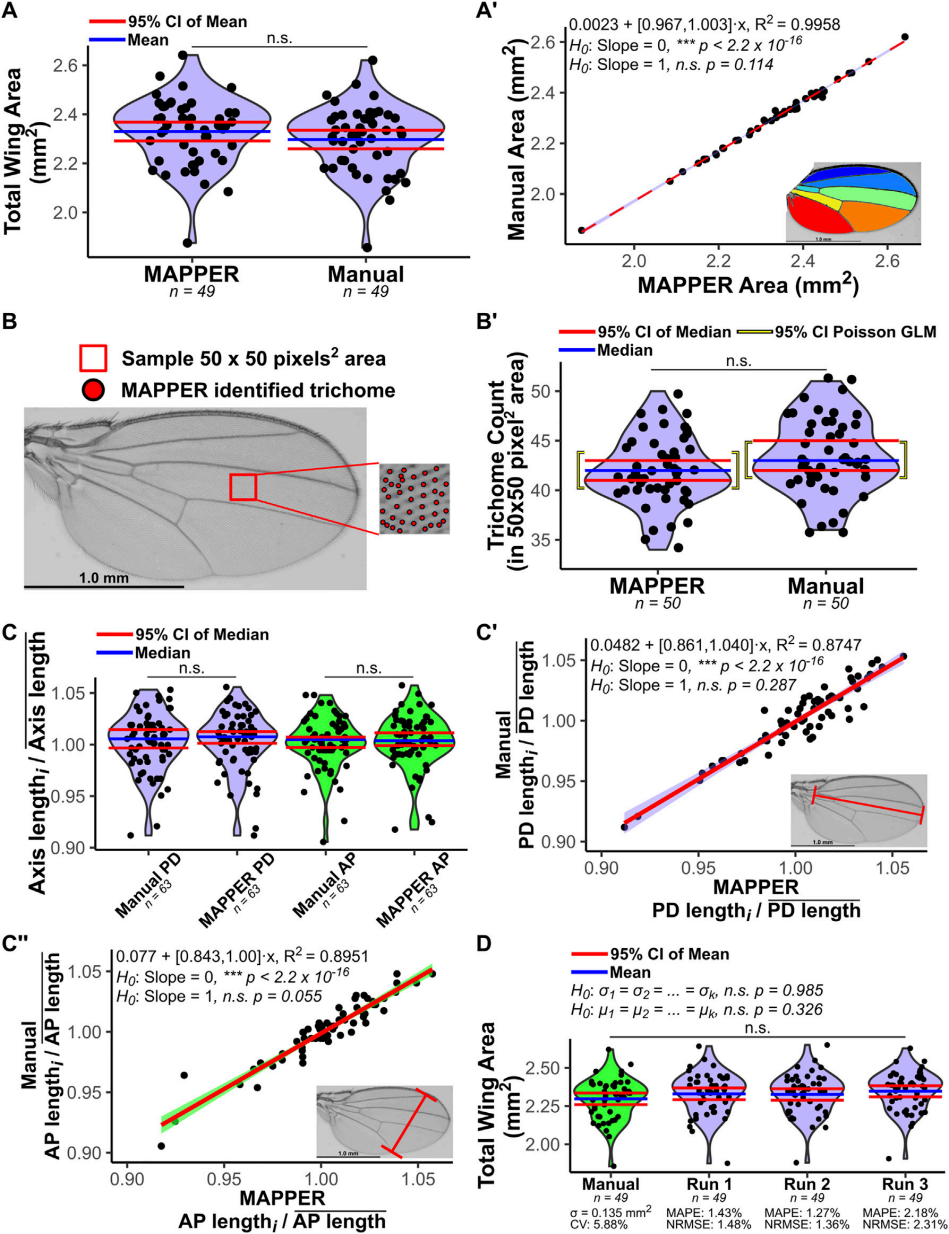
Validation of automated measurements generated by MAPPER. Manual measurements were taken and compared to MAPPER’s output. **(A)** Total wing area measurement distributions of automated and hand measurements are compared. An F-test (Snedecor and Cochran, 1989) compared the variances of the distributions (*p* = 0.928), and an unpaired T-test (Fisher, 1925) compared the means (*p* = 0.236). **(A′)** A linear regression fit of automated versus manual measurements (red dashed line corresponds to the fit, and light-blue bands correspond to the 95% CI of the fit). The slope parameter of the fit was not statistically different from a value of 1.00 (*p* = 0.114). A color-coded image of a MAPPER-processed wing is shown in the inset. **(B)** Trichome count was validated by observing a small 50 × 50 pixel^2^ area between the third and fourth longitudinal veins. Identified trichomes by MAPPER are shown as red circles overlaid onto the raw image. **(B’)** Trichomes were counted using MAPPER and manually. 95% CIs are based on order statistics (Harrell and Slaughter, 2021). A Poisson regression generalized linear model (GLM) was fit to the trichome counts with either MAPPER or manual measurements being a categorical explanatory variable. **(C)** The landmark region measurements of proximal-distal (PD) axis or anterior-posterior (AP) axis were manually measured and compared to MAPPER’s output. The axis length was scaled to the mean axis length of the group. Violin plot distributions compare MAPPER’s output to manual measurements. Scaled landmark region lengths via MAPPER are not statistically different from manual measurements for the PD axis (*p* = 0.802) or for the AP axis (*p* = 0.760) *via* Mann-Whitney U Test (Mann and Whitney, 1947; Nachar, 2008). **(C’,C’’)** A linear regression fit to automated versus manual measurements (red line) was fit to the points for the PD axis (C’) and AP axis (C’’) measurements. The 95% confidence bands of each fit are overlaid in light-blue for PD axis measurements and light-green for AP axis measurements. The slope parameters of each fit was not statistically different from a value of 1.00 (*p* = 0.287 for PD axis and *p* = 0.055 for AP axis). Inset: Raw wing image with a corresponding landmark measurement is shown as a red line. **(D)** MAPPER was run three independent instances on the same dataset (labelled runs 1–3). The resulting output measurements for total wing area were compared to manual measurements. Variances of each distribution were not statistically different (Bartlett’s test for homogeneity of variances (Snedecor and Cochran, 1989), *p* = 0.985). Means of each distribution were not statistically different (one-way ANOVA (Snedecor and Cochran, 1989; Chambers et al., 1992), *p* = 0.326). The mean absolute percentage errors (MAPE) of the independent MAPPER runs ranged between 1.27 and 2.18% when comparing MAPPER predicted values to true manual measurements (Makridakis et al., 1982; Bowerman, 2005). The root-mean-square errors normalized to the mean of the manual measured data (NRMSE) of the independent MAPPER runs ranged between 1.36 and 2.31% (Poli and Cirillo, 1993; Hyndman and Koehler, 2006). The coefficient of variation (CV) of the manual measurements serves as a proxy for the error that naturally occurs scaled to the mean when taking manual measurements (Everitt, 2002). Data in A, A’, B, B’, and D are female, and data in C, C’, and C’’ are male Samarkand strain wings. Data available from (Sonnenschein et al., 2015).

MAPPER also quantifies the AP and the PD axes lengths of the wing blade (Figures 3C,D). The labelled interveins are used to delineate the L_2_, L_3_, L_4_, and L_5_ veins and the cross veins (Supplementary Figure S6). Identification of veins is accompanied by quantification of landmark positions within the wing. Further, the number trichomes of each labelled intervein region (corresponding to cell number) and trichome density of the region are quantified as extracted features. Thus, MAPPER extracts a high-dimensional fingerprint of morphological and shape features.

### MAPPER’s Automated Measurements are Statistically Identical to Manual Measurements

We benchmarked MAPPER’s accuracy and precision in replicating manual measurements for 112 adult wing images (*n* = 49 females and 63 males) of *Drosophila melanogaster* from the Samarkand strain (Sonnenschein et al., 2015). To prevent artificially high coefficients of determination (R^2^) in linear regression models, male and female measurements were analyzed separately. Total wing area measurements for female wings from MAPPER’s automated output were compared to measurements taken manually in ImageJ software using the Polygon selection tool (Figure 3A). An F-test (Snedecor and Cochran, 1989) demonstrated the two variances of the distributions were not statistically different (*p* = 0.928) and an unpaired T-test (Fisher, 1925) demonstrated the two means of the distributions were not statistically different (*p* = 0.236). A linear regression model was fit to the manual measurements plotted against the automated measurements (R^2^ = 0.996) and the slope parameter of the fit was found to not be statistically different from a value of 1.00 (*p* = 0.114), indicting a one-to-one correspondence of manual and automated measurements (Figure 3A’).

We also validated MAPPER’s automated measurements compared to manual measurements for all individually labelled intervein regions (Figure 2A, Supplementary Figure S7, Supplementary Figure S8). MAPPER’s measurements were statistically identical to manual measurements for intervein regions 1 through 6 (*p* > 0.05). MAPPER slightly overestimated the area of intervein region 7 (Supplementary Figure S8), which may be attributed to how the erosion/dilation operations perform when handling the partial L_6_ vein (Blair, 2007). However, the slope parameter of the fit for this region had a 95% CI of (0.919, 0.985), which indicates that the difference between the automated and manual measurements was slight (no more than 0.081 mm^2^ per 1 mm^2^ increase in overall wing size). Even when slight variations between measurements were seen, overall, MAPPER consistently produces measurements statistically identical to manual measurements.

Further, we validated MAPPER’s accuracy in quantifying trichomes by analyzing a small 50 × 50 pixel^2^ area between the third and fourth longitudinal veins of the male wings (Figure 3B). Trichome numbers were counted manually and by using MAPPER (Figure 3B’). A Poisson regression generalized linear model (GLM) was fit to the trichome counts with either MAPPER or manual measurements being a categorical explanatory variable (Figure 3B’). The exponential of the fit parameter associated with MAPPER versus manual measurements has a 95% confidence interval (CI) of (0.974, 1.098), indicating there is no statistical difference whether trichome counts come from MAPPER or manual measurements (*p* = 0.271, Supplementary File S2).

Next, MAPPER’s automated output measurements were compared to manual measurements for male wings for measurements of PD and AP axes lengths. For each case, the axes length measurements were normalized to the mean axis length of their respective groups. Normalized landmark region lengths (Figure 3C) measured by MAPPER are not statistically different from manual measurements for the PD axis (*p* = 0.802) nor for the AP axis (*p* = 0.760) *via* Mann-Whitney U Test (Mann and Whitney, 1947; Nachar, 2008). Further, when fitting a linear regression model to manual measurements plotted against MAPPER’s automated measurements, the slope parameters of each fit were not found to be statistically different from 1.00 (*p* = 0.287 for PD axis and *p* = 0.055 for AP axis) indicating a one-to-one correspondence of manual and automated measurements for landmark axes lengths (Figures 3C’, C’’).

To test precision, MAPPER was run three independent instances on the same dataset starting with pre-processing of the raw wing data, ILASTIK pixel-classification training, and finally processing by MAPPER (Figure 3D). The resulting output measurements for total wing area for each independent run were compared to each other and manual measurements. Bartlett’s test for homogeneity of variances (Snedecor and Cochran, 1989) determined the variances of each distribution were not statistically different (*p* = 0.985). A one-way ANOVA (Snedecor and Cochran, 1989; Chambers et al., 1992) test determined the means of each distribution were not statistically different (*p* = 0.326). The mean absolute percentage errors (MAPE) of the independent MAPPER runs ranged between 1.27 and 2.18% (Figure 3D, Supplementary File S2) when comparing MAPPER predicted measurements to true manual measurements (Bowerman, 2005; Makridakis et al., 1982). The root-mean-square errors normalized to the mean of the manual measured data (NRMSE) of the independent MAPPER runs ranged between 1.36 and 2.31% (Figure 3D, Supplementary File S2) (Poli and Cirillo, 1993; Hyndman and Koehler, 2006). The coefficient of variation (CV) of the manual measurements can be used as a proxy for the amount of error that naturally occurs scaled to the mean when taking manual measurements (Everitt, 2002). Because RMSE is an estimator for the standard deviation of the distribution of the MAPPER predicted residuals, benchmarking MAPPER NRMSEs to the manual measurement CV value of 5.88% suggests MAPPER total wing measurements are within the range of naturally occurring error of taking manual measurements (Shmueli et al., 2017). Overall, MAPPER’s automated measurements are both accurate and precise in comparison to manual measurements for total wing area, intervein region areas, trichome counts, and landmark axes lengths.

### Benchmarking MAPPER performance

As a second validation step, we benchmarked MAPPER’s performance against a currently available wing analysis pipeline, FIJIWings, for wings of varying size. This was done through a systematic comparison of metrics, such as wing blade area and trichome density, for wings generated by genetically perturbing insulin signaling. Insulin and insulin-like growth factors regulate metabolic activity (Kurtzhals et al., 2000; Samani et al., 2007; Belfiore et al., 2009). Dysregulation of insulin signaling causes a variety of human diseases including diabetes, insulinoma, metabolic syndrome, ovary syndrome, and auto-immune disorders (Dunaif et al., 1989; Kahn et al., 2006; Wang et al., 2017). In *Drosophila,* the InsR homolog regulates cellular proliferation (Brogiolo et al., 2001). Loss of function of InsR in wing imaginal discs reduces final wing size (Chen et al., 1996).

**FIGURE 4:**
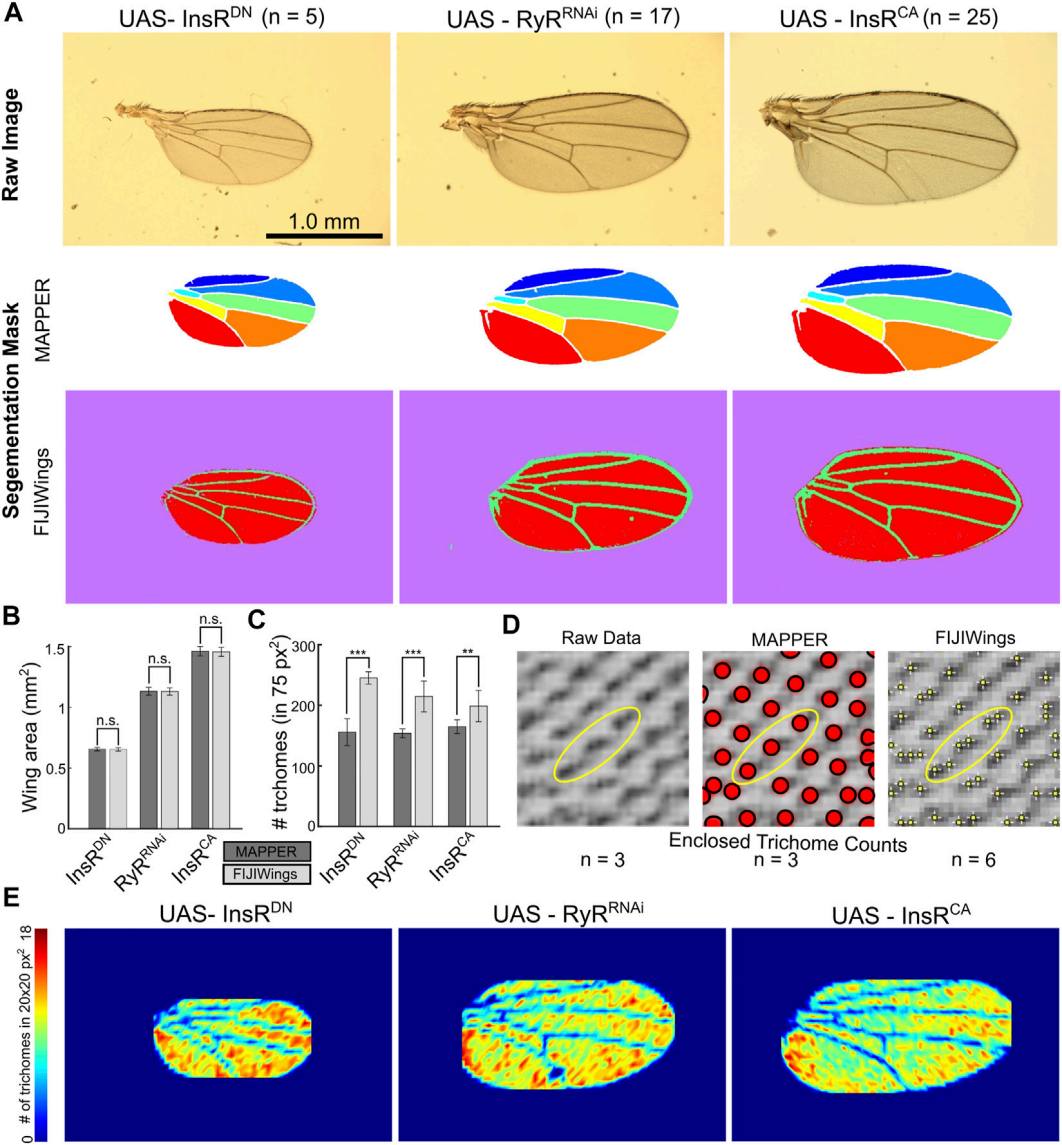
MAPPER provides precise segmentation and extraction of wing shape and trichome density. **(A)** MAPPER automates identification and labeling of the individual intervein components with high accuracy. InsR^DN^ is the dominant negative form of the insulin receptor, and InsR^CA^ is the constitutively active insulin receptor. RyR^RNAi^ is the GAL4-UAS knockdown of the ryanodine receptor, which is not known to be expressed in the wing disc. Full genotypes are the Nubbin-Gal4, UAS-Gcamp6f lines crossed to each of the indicated UAS lines. **(B,C)** Comparison of wing area and number of trichomes in a 75 × 75 pixel^2^ bounding box, respectively, as calculated by MAPPER and FIJIWings. Error bars indicate standard deviation in measurements. Statistical comparisons for wing area were performed via the unpaired T-test and F-test (*p >* 0.05, n. s.). Statistical comparisons for trichome counts were performed via the Mann-Whitney U Test (Mann and Whitney, 1947; Nachar, 2008) for nonparametric comparisons (*p* < 0.001 ***, *p <* 0.01 **). **(D)** Comparison of trichome location estimation between MAPPER and FIJIWings with respect to the raw image. **(E)** Heatmaps representing trichome density. The wing is binned into regions of 20 × 20 pixel^2^ areas. Number of trichomes are calculated using MAPPER in wing subregions.

As expected, quantification of wing size shows that activation of InsR signaling increases wing size. Conversely, suppression of InsR signaling reduces wing size (Brogiolo et al., 2001). The overall area of wings were comparable (*p* > 0.05 for unpaired T-tests and F-tests, Supplementary File S2) when they were measured through FIJIWings and MAPPER (Figures 4A,B). However, FIJIWings over-segmented tissues as regions containing marginal wing hairs were often misclassified as intervein regions (Figure 4A). This was not observed in any of the segmentation masks produced by MAPPER.

MAPPER and FIJIWings were then compared in their ability to quantify the number of trichomes in a 75 × 75 pixel^2^ area cropped from the seventh intervein region. This metric was chosen after validation of MAPPER trichome counts being statistically identical to manual counts (Figure 3B-B’). The median count of trichomes upon activation of InsR were higher than the RyR^RNAi^ control (*p* = 1.35 × 10^–03^). However, suppression of InsR did not have a significant change in the trichome counts (*p* = 0.845) (Figure 4C). When comparing trichome counts by MAPPER to those of FIJIWings, there was a significant difference in number of trichomes estimated by FIJIWings and MAPPER (Figures 4C,D). In particular, FIJIWings predicted a greater number of trichomes compared to MAPPER for downregulated insulin signaling (*p* = 7.94 × 10^–03^), for a control group (*p* = 2.15 × 10^–06^), and for upregulated insulin signaling (*p* = 5.59 × 10^–06^) (Figures 4C,D, Supplementary File S2). To visualize the discrepancies of trichome counts predicted through FIJIWings and MAPPER in a sample 75 × 75 pixel^2^ area, identified trichome locations were plotted (Figure 4D). FIJIWings showed an overestimation in predicted number of trichomes, as confirmed by the measured data (Figure 4C). Estimation of trichome density within a small region of the wing is not sufficient to quantify global changes in trichome density. To do so, MAPPER was next used to first estimate the location of trichomes within the intervein region. The overall wing domain was then binned into small subdomains of 20 × 20 pixel^2^ areas. The number of trichomes in those sub-domains were then used to create heat maps representative of local trichome density within the wing samples (Figure 4E, Supplementary Figure S9). This highlights that a suppression of InsR during wing development leads to an increase in trichome density, consistent with insulin’s role in regulating cell size. Taken together, these results highlight MAPPER’s ability to more accurately and precisely estimate both wing area and trichome counts compared to a previous pipeline.

## Results: Case Studies

### High-Dimensional Phenotypic Exploration of Sexual Dimorphism

**FIGURE 5:**
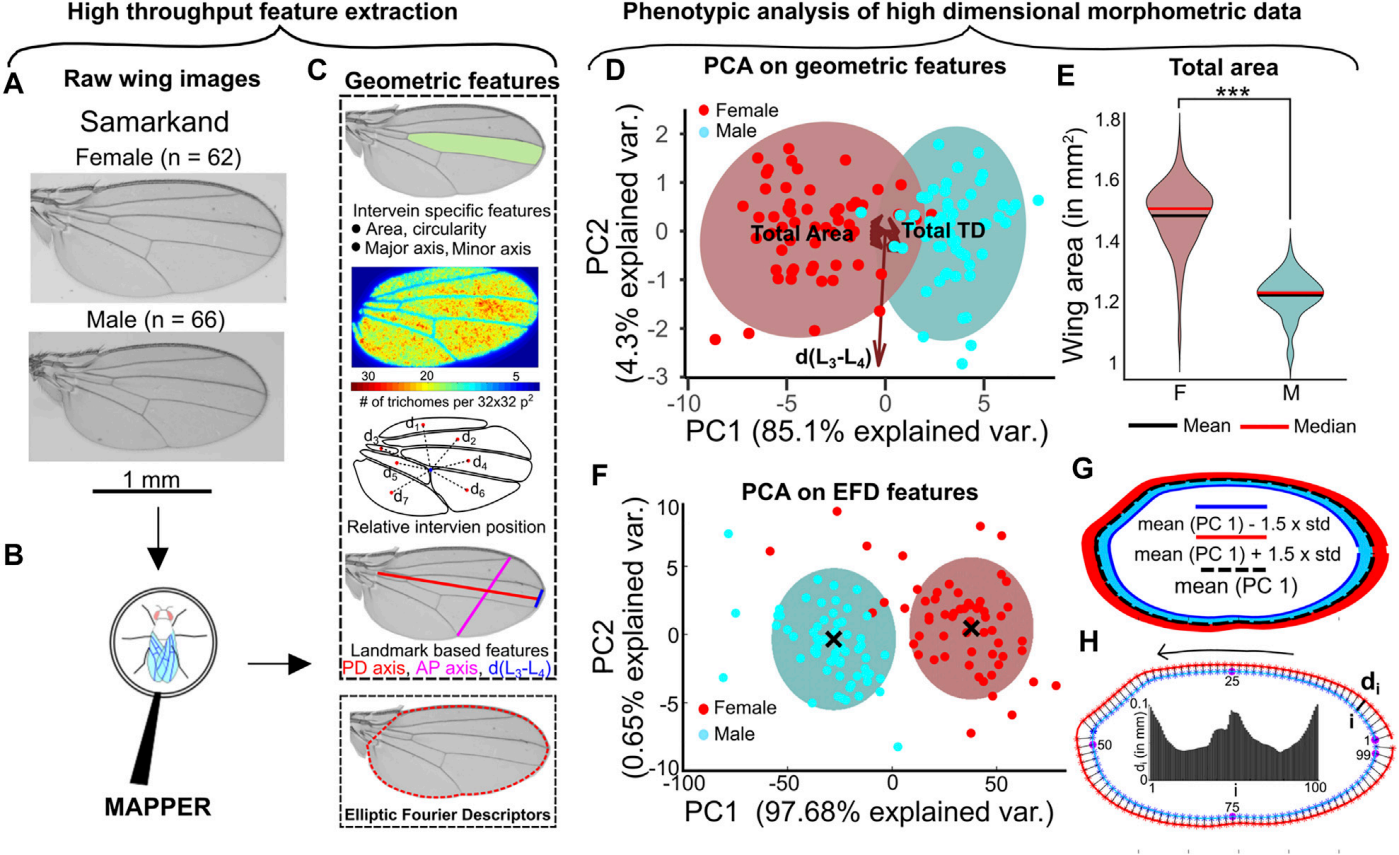
Representative statistical approaches for phenotypic analysis. **(A)** Male and female Samarkand wings (Sonnenschein et al., 2015), with sample sizes indicated, were analyzed and processed **(B)** to demonstrate MAPPER’s phenotypic profiling features. **(C)** Definition of the wide number of geometric/morphological features including extracted EFD coefficients fit to the wing margin. **(D)** PCA reveals the largest variance in the data observed in terms of overall wing area and trichome density. As expected, two distinct clusters are evident when plotting the first to PCs. **(E)** Violin plot showing the distribution of area of male and female wings. Solid red line indicates the median and solid black line indicates the mean of each population. **(F)** PCA on EFD revealed most of the variance in the data concentrated only in the first principal component. Two distinct clusters separate the male and female populations. **(G)** Standard deviation in the direction of PC1 was calculated for the entire population of wings. PC1 was varied by adding and subtracting 1.5 times the standard deviation along PC1. Reverse PCA was then used to obtain the desired EFD coefficients in which the contours were reconstructed. **(H)** EFD was used to construct mean wing shapes representing the male and female populations. 100 points were sampled from the male wing and their minimum distance from the female wing was calculated to quantify local size differences within the two populations. The variation of size is drawn as a bar graph where the x-axis is representative of the points sampled in male wing. The locations of points sampled are indicated in the plot.

The sex-based differences in overall size of adult *Drosophila melanogaster* wings is well documented (Testa and Dworkin, 2016). To further study this dimorphism and to investigate whether additional, more subtle differences are detectable, we processed 128 Samarkand strain wings with MAPPER (Figure 2B, Figures 5A,B) (Sonnenschein et al., 2015) to create a high-dimensional morphometric fingerprint of each sample (Figure 5C). Principal Component Analysis (PCA) (Wold et al., 1987) carried out on the geometric features revealed that the maximum variance (89.4%) within data was distributed majorly between the first two principal components (Figure 5D, Supplementary Figures S10A–C). Analysis of the loadings for the first principal component showed that overall wing blade area explains the majority of variance within the data. As expected, the area of the female wing was significantly larger than a male wing (*p* < 0.001) (Figure 5E, Supplementary Figure S10E). A plot of the first two principal components shows the two distinct clusters of male and female populations (Figure 5D).

To highlight the utility of EFDs for phenomic analysis of wing shape, EFD coefficients were separately analyzed from the other geometric features. PCA applied on the EFD coefficients revealed a total variance of about 97% distributed along PC1 alone (Figure 5F). The observed variance along PC1 is attributed to the known overall size differences between the male and female population of wings (Figure 5E). Clustering carried out on the first two principal components using Gaussian Mixture Models (Yang et al., 2012) (GMM) was also able to distinguish the male and female population of wings (Figure 5F). The mean shapes of each cluster can also be used to highlight local shape changes between the male and female populations. We used the mean contours of each population and measured variation peripheral growth along the normal direction (Figures 5G,H). There is more growth along the PD axis compared to the AP axis, which is necessary for maintaining a uniform scaling of these anatomical axes with overall size of wing blade (Supplementary Figure S11). This uniform scaling also confirms that the normalized length of the AP and PD axes are equal for both the male and female wings.

**FIGURE 6:**
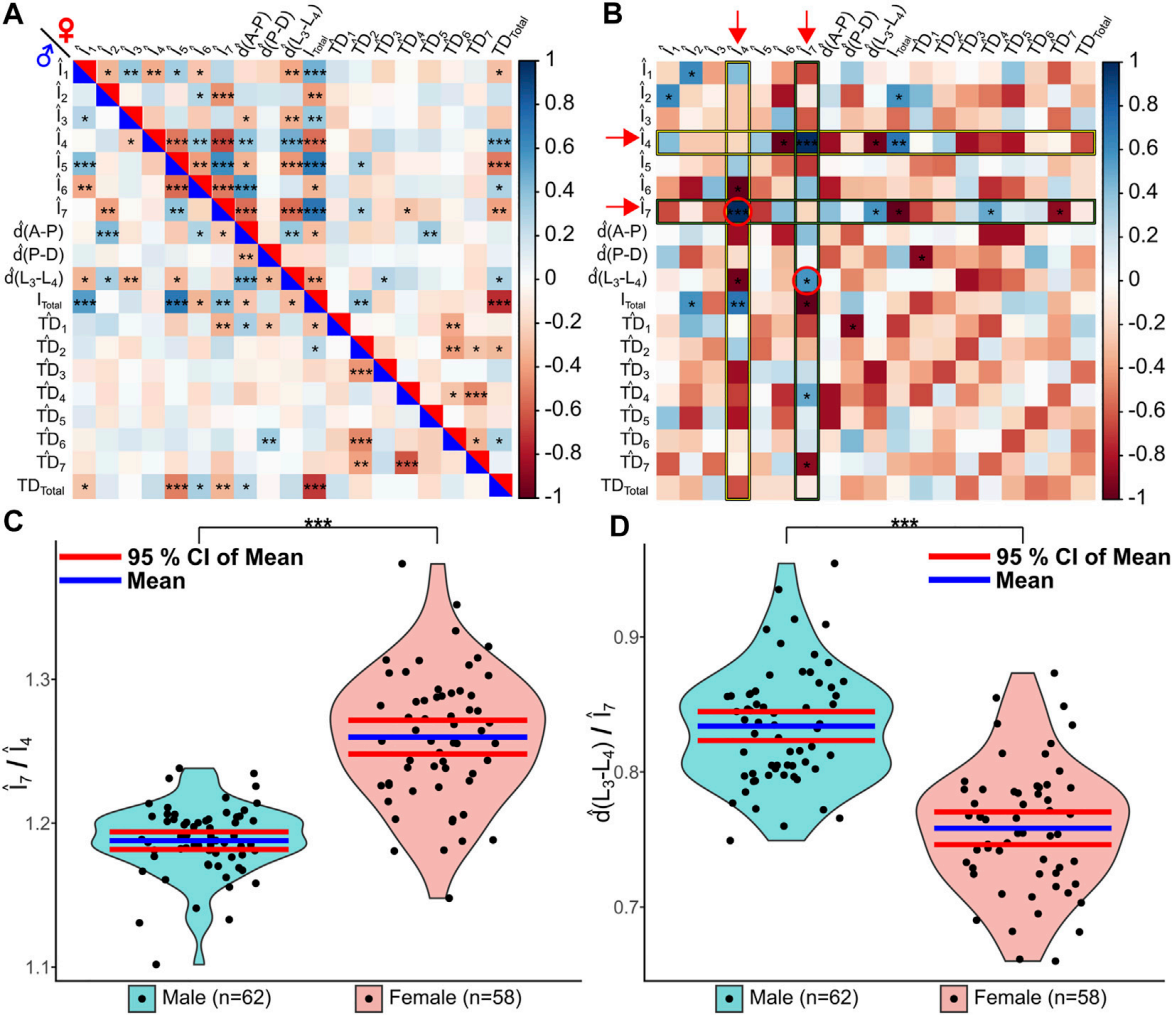
MAPPER identifies unique scaling relationships between wing populations. MAPPER output data of male and female Samarkand strain wings (Sonnenschein et al., 2015) were analyzed to identify potential scaling relationships between features. All geometric features were normalized to become unitless features, denoted by the hat symbol. **(A)** A correlation plot of the features for males (lower-left triangle) and females (upper-right triangle) is shown. Significance of correlation is denoted by asterisks with *p* < 0.05 *, *p* < 0.01 **, and *p* < 0.001 ***. **(B)** Fisher’s Z-transformation (Fisher, 1915; Fisher, 1921) was performed on the correlation coefficients to observe differences in underlying correlations between the two populations. Significance of correlation is denoted by asterisks with *p* < 0.05 *, *p* < 0.01 **, and *p* < 0.001 ***. Z-scores were scaled to be between -1 and 1 for plot simplicity. Red arrows indicate significant correlations plotted as violin plots on subsequent figure panels. **(C,D)** The underlying correlation differences between Î_7_ and Î_4_ (C) and between $\hat{d}$ (L_3_-L_4_) and Î_7_ (D) were found to be significant by Fisher’s Z-transformation between populations. Statistical tests were performed using the Mann-Whitney U Test (Mann and Whitney, 1947; Nachar, 2008) for nonparametric comparisons (*p* < 0.001 ***).

We further investigated potential scaling relationship differences between the two wing populations. We first normalized all geometric wing feature measurements produced by MAPPER. More information on normalization approaches are detailed in the methods section. Overall, each feature was normalized such that they were unitless to enable comparisons across male and female population despite known size differences. A correlation plot of the normalized features reveals that there are significant scaling relationships of wing features for both male and female populations (Figure 6A). We identified several underlying significant differences in scaling relationships between male and female wings after applying a Fisher’s Z-transformation (Fisher, 1915; Fisher, 1921) on correlation values. Of note, females have a relatively larger normalized intervein region 7 (the most posterior region) scaled to intervein region 4 (related to high levels of Hedgehog and Decapentaplegic signaling) (Figures 5B,C, *p* < 0.001). Males had a significantly larger normalized d(L_3_−L_4_) scaled to intervein region 7 (Figures 6B,D, *p* < 0.05). These results suggest the presence of underlying sex-specific differences in morphogen signaling in male and female wings during development (Surkova et al., 2021). Further investigation of the identified scaling differences from the Fisher’s Z-transformation would enable linking of the relationships to the governing genes that regulate wing morphogenesis. We explore these scaling relationships in more detail in a case study conducted on wings belonging to four different species of *Drosophila*.

### MAPPER Reveals Species-Specific Differences in Wing Size and Developmental Patterning

**FIGURE 7:**
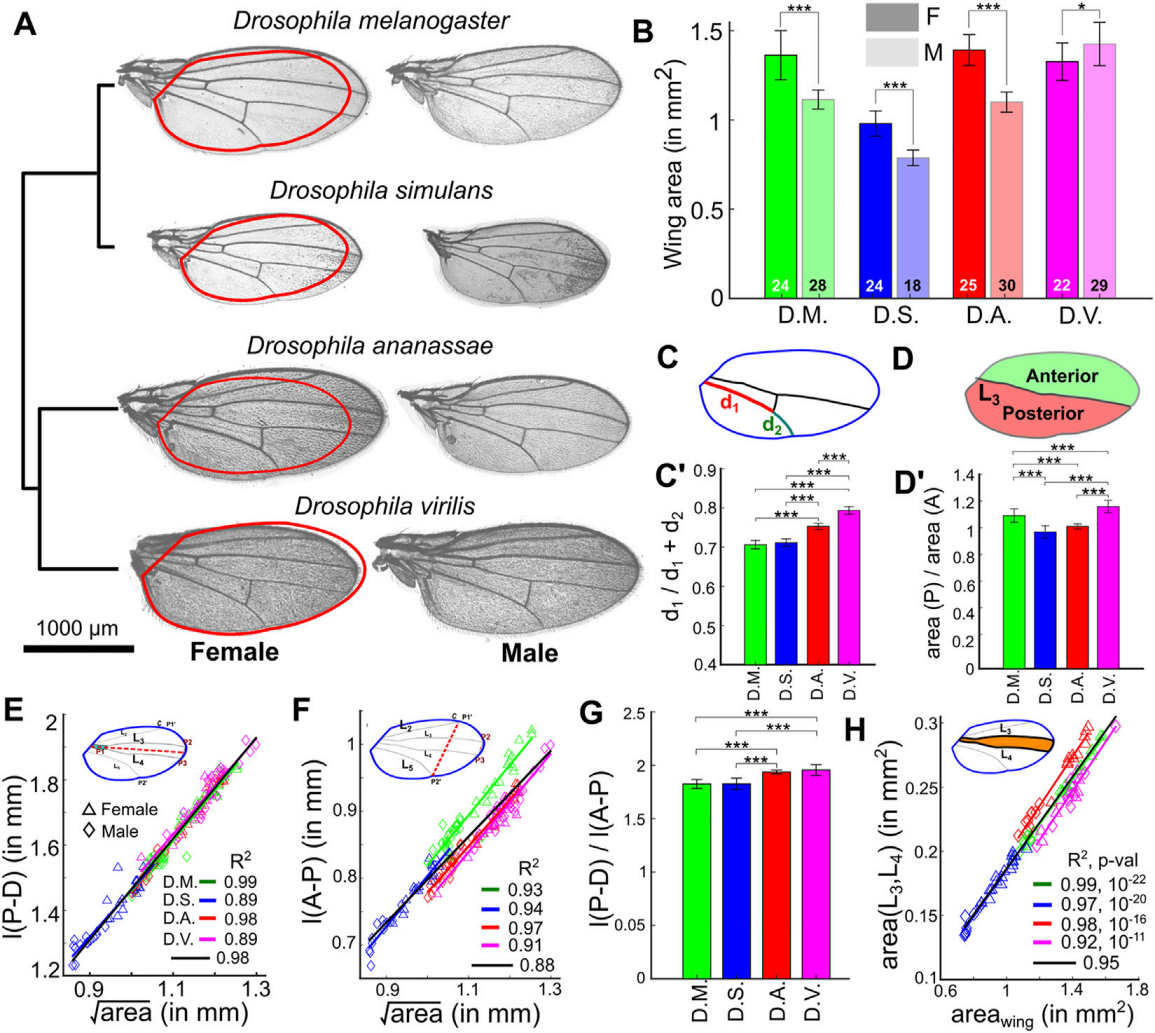
MAPPER identifies differences in wing size and scaling relationships across *Drosophila* species. **(A)** Representative male (right) and female (left) wings for four different species. Red contours on female wings represent the outline of the corresponding male wing. The dendrogram in the left is representative of hierarchical clustering based on different features extracted using the pipeline. **(B)** Quantification of wing areas by MAPPER for wings from different species and different sexes. **(C-C’)** Quantification of shift in posterior cross vein position in female wings (d_1_ is defined as the segment of L_5_ from the proximal end of the vein to posterior cross vein, d_2_ is defined as the segment of L_5_ from the posterior cross vein to the distal end of L_5_). **(D-D’)** Relative anterior (A) and posterior (P) areas in female wings. **(E)** Scaling relationships between the length of the proximal-distal [l (P-D)] axis and the overall wing blade area for the various species. Legends for different sexes have been included. Straight lines were fit to estimate the existence of scaling relationships for the four species. **(F)** Scaling relationships between the length of the anterior-posterior [l (A-P)] axis and the overall wing blade area for the four species. **(G)** The l (P-D) to l (A-P) ratio for females from different species. **(H)** Scaling relationships between the area of the intervein region between veins L_3_-L_4_ and the overall wing blade area for the four species. Straight lines were fit to estimate the existence of scaling relationships for the different species. (*p < 0.05, **p < 0.01, ***p < 0.001).

Next, we quantified morphometric phenotypes of wings for four species: *D. melanogaster*, *D. simulans* (a species in the *melanogaster* subgroup), *D. ananassae* (a species in the *melanogaster* group), and *D. virilis* (a species outside the *melanogaster* group) (Da Lage et al., 2007). Wing area was revealed to be larger in females when compared to males in *D. melanogaster, D. simulans*, and *D. ananassae* (Figure 7A). Interestingly, adult wings are larger in males than in females in *D. virilis* (Figures 7A,B). We found size-independent differences among species, especially in females (Supplementary Figure S12A). The relative location of the posterior cross vein that connects the longitudinal veins L_4_ and L_5_ is approximately the same for *D. melanogaster* and *D. simulans* but is located more distally in *D. ananassae* and *D. virilis* (Figure 7C-C’, Supplementary Figure S12B).

We also found species-specific differences in the relative areas of anterior (A) and posterior (P) regions of the wing (Figure 7D). In *D. simulans* and *D. ananassae* the A and P regions are approximately of the same size, but in *D. melanogaster* and *D. virilis* the P region is about 10 and 20% larger than the A region, respectively (*p* < 0.001) (Figure 7D’, Supplementary Figure S12C). Details about the tests for determining the statistical significance of these comparisons can be found in Supplementary File S1 Section S6, while individual *p*-values are listed in Supplementary Figure S13. It should be noted that the dataset processed for this analysis did not have sufficient resolution for accurate analysis of trichome density patterns.

### Not all Anatomical Axes are Equally Scaled Across Species

Along the PD axis, all species follow a similar linear scaling relationship with respect to the square root of the total wing area (Figure 7E), suggesting that there is strong selection in maintaining a proportional PD length across species. Along the AP axis, we also observe a linear scaling relationship for the AP length, but the slopes vary from species to species (Figure 7F). To further explore these results, we plotted the ratio of the PD and AP axes lengths and found that *D. ananassae* and *D. virilis* have a slight but significantly larger ratio than *D. simulans* and *D. melanogaster* (Figure 7G, Supplementary Figure S12D). Taken together, these data suggest that the relative length of the AP axis in *D. ananassae* and *D. virilis* is significantly shorter compared to that of *D. simulans* and *D. melanogaster*, suggesting that variation in AP axis correlates with the phylogenetic split between pairs of species (Figure 7A).

In *Drosophila*, the AP axis is patterned by two morphogens (Figure 1A): Hedgehog (Hh) and Decapentaplegic (Dpp) (Blair, 2007). Hh patterns the most central region (L_3_-L_4_ veins) (Vervoort et al., 1999; Mohler et al., 2000), whereas Dpp patterns the positions of L_2_ and L_5_ (Affolter and Basler, 2007; Restrepo et al., 2014). To pinpoint whether the changes we see along the AP axis could be attributed to any of these signaling pathways, we compared the L_3_-L_4_ intervein area in these species (Figure 7H). We found that these similarly scale in all four species, suggesting that it is unlikely that these differences are due to variations in the regulation of the Hh signaling pathway. Interestingly however, the areas comprising veins L_2_ and L_5_ with respect to the wing margin appear to scale differently across species (Supplementary Figure S12A), suggesting that Dpp signaling dynamics varies across species to regulate the proportions of these wings along the AP axis. As a prediction for future studies, these results are suggestive that Dpp transport and/or transduction is variable, while Hh is not, across species. In sum, MAPPER proved to be a powerful toolkit for generating new hypotheses about morphogenetic relationships across *Drosophila* species.

## Discussion

### Features and Strengths of MAPPER as a Robust Tool for *Drosophila* Wing Phenomics

Conventional image processing techniques are often unable to process images of model organism morphologies that have been generated with different imaging systems. For example, traditional image processing pipelines have difficulty analyzing images taken with multiple different lens objectives, lighting conditions, or rotational orientations. As such, these pipelines often fail at accurately processing images beyond the initial dataset for which it has been developed.

MAPPER supersedes previous pipelines by using a statistical learning framework, with the latest computer vision and ML approaches, to compartmentalize a wing accurately and precisely into different regions. In previously established pipelines, the number of morphometric features that are extracted are low-dimensional, making them unsuitable for detecting subtle quantitative changes that can be mapped back to differential gene regulation. A key feature of MAPPER is its hybrid, modular framework. The first component is a DL-based pixel classification module that segments individual regions of wings. The second module labels each intervein region according to its shape-based features. In conjunction, these individual pipelines allow MAPPER to generate a wide variety of geometrical and pattern-based features of wing images. The precise labelling of interveins allows for reconstruction of veins and automated extraction of landmark-based measurements, such as the AP and PD axes. In summary, the coupling of two modules with an integrated diverse class of functions, automates the systematic generation of high-dimensional geometric and pattern-based features for a large volume of wing image data.

### Implications of Insights Generated by MAPPER

The analysis of adult wings in different *Drosophila* species using MAPPER reveals two key observations. First, we noticed a reversal in sexual dimorphism when comparing species within the *melanogaster* group with *D. virilis*. Particularly, wings of *D. melanogaster*, *D. simulans*, and *D. ananassae* are larger in females than in males. However, in *D. virilis* the opposite phenotype is observed (Figures 7A,B). How wing size is differentially regulated in a sex-specific manner across species is unclear, but our data suggest that the dimorphism that makes female wings larger than male wings arose at some point in the divergence between the *melanogaster* and *virilis* groups. Second, the length of the wing PD axis across species and sexes shows a conserved scaling relationship with respect to wing size (Figure 7E), suggesting that while ecological and genetic changes may exert pressure on overall wing size, preserving a scaling relationship between length of the PD axis and total wing area in all species may be essential. In contrast, the AP axis in *D. ananassae* and *D. virilis* is smaller with respect to what would be predicted from the scaling relationship of *D. melanogaster* and *D. simulans* (Figure 7F). Since BMP/Dpp signaling is responsible for patterning and growth along this axis, we predict the variation in this pathway between species can explain the larger AP axis in *D. melanogaster* and *D. simulans.* Variation in Dpp pathway activities between species may also explain why the posterior cross vein is located more distally in *D. ananassae* and *D. virilis* compared to *D. melanogaster* and *D. simulans* (Figure 7C-C’). This is because the specification of the posterior cross vein depends on pupal BMP signaling driven by Dpp and Glass-bottom-boat (Gbb) ligands (Ray and Wharton, 2001).

### Current Limitations and Future Extensions

The new findings from these case studies demonstrate that, in the current age of big data phenomics, manual phenotypic characterization provides an incomplete characterization of phenotypic variation in samples. Here we present a novel, hybrid ML-based approach that was used to automate high throughput measurements of adult *Drosophila* wings. With the extensive research documenting gene expression profiles and genotypes in *Drosophila* wings, the phenomics data produced by MAPPER can be used to bridge quantitative sciences to genomics from analysis of phenomes induced by genetic perturbations (Pitchers et al., 2019). By performing a genome-wide association analysis linking features measured by MAPPER to genotypes, a gene regulatory network of genes associated with phenotypes can be established. Therefore, MAPPER has the capability to be used as a computational tool to identify genetic variations that contribute to gene-related diseases.

The image segmentation capabilities of MAPPER can be easily extended to any insect wing by using training datasets from different imaging sources and multiple insect species. A particular strength of MAPPER is its automated intervein classification module. A current limitation is that output measurements resulting from low-resolution or obscured image input, will produce inaccurate results or inaccurately labelled intervein regions. However, this is expected for any image-based ML approach where the output is largely dependent upon the quality of the input image. In the future, MAPPER can also be extended to perform phenotypic analysis at a whole organism level. Recently, there have been several attempts to extend depth of field and multi-view imaging of insects (Ströbel et al., 2018). The advancements in the field of DL-based smartphone imaging has allowed smartphones to be used for the acquisition of multiview datasets. Integration of algorithms such as Multi-View Deep Extreme Learning Machine (MVD-ELM) can easily be used for the task of 3D segmentation of specific organs (Xie et al., 2015; Ahmed et al., 2019). In summary, MAPPER rigorously fits form to functions for a broad range of applications that can range from comparative genomics, drug target discovery, and phenotypic screening.

## Materials and Methods

### Fly Culture, Wing Collection, and Imaging

Wing-specific GAL4 drivers were grown at 25°C. Virgins were collected twice a day from the bottles. Virgins were crossed with males that carry the indicated UAS-TRiP line constructs in a ratio of female:male of 10:3. Adult flies were harvested within 7 days of eclosure. Wings were removed and mounted on microscopy slides to obtain high resolution images. 360 wings were analyzed in different case studies for this paper. Wings were placed in ethanol, and approximately 15 wings were mounted on each slide in Permount medium (Fisher Scientific, SP15) using standard procedures. For the benchmark experiments related to InsR, slides were batch-imaged using an EVOS microscope at ×4 magnification. For the case study involving wings from four *Drosophila* species, the wings were incubated overnight in 70% ethanol. Wings of different *Drosophila* species were imaged using a Nikon Eclipse Ci-S microscope using a Jenoptik ProgRes® monochromatic camera and the ProgRes® Capture Pro 2.9 software.

### Description of Computational Platform

MAPPER is available in the form of a MATLAB-based GUI for both individual and batch analysis of wings. The design of MAPPER also allows users with preliminary knowledge of MATLAB to integrate their custom functions estimating any new desired geometric feature. Details about the design, use and MAPPER application are provided in Supplementary File S1 Section S1–S4. The code repository, instructions to run the MAPPER application, available segmentation modules, trained U-Net model, and the data used to produce figures, can all be found on MAPPER’s dedicated GitHub Page here https://multicellularsystemslab.github.io/MAPPER/ (https://multicellularsystemslab.github.io/MAPPER/). Additionally, an in-depth user manual and guide for the MAPPER application can be downloaded here.

### Additional Notes on Statistical Analysis

For Figure 6, all intervein region measurements were normalized to their respective total wing area (I_Total_) to become unitless features (Î_i_ for *j* = 1 to 7 regions). Trichome densities (TDs) were calculated by dividing trichome counts in an intervein region by the intervein area of the region. These TDs were then normalized to their respective total trichome density (TD_Total_) to become unitless features ( $\hat{TD}$ _i_ for *i* = 1 to 7 regions). Landmark features were normalized to their respective square root of total wing area to become unitless features ( $\hat{d})$ where P-D is the distance of the proximal-distal axis, A-P is the distance of the anterior-posterior axis, and L_3_-L_4_ is the distance between the L_3_ and L_4_ veins. To assess statistically significant differences in the scaling relationships between the two populations, Fisher’s Z-transformation (Fisher, 1915; Fisher, 1921) was performed on the correlation coefficients (Figure 6B). This transformation effectively transforms the correlation coefficients into normally distributed values with which statistical tests can be performed to compare the Z-scores between two groups. For Figure 7, all *p*-values corresponding to comparisons made can be found in Supplementary Figure S13 with details on the statistical tests being explained in Supplementary File S1 Section S5.

**Table:** 

Fly lines	Source	Stock #	Genotype
MS1096-GAL4, UAS Dcr2	BDRC	25,706	w (1,118) P[w (+mW.hs) = GawB]B x (MS1096); P[w (+mC) = UAS-Dcr-2.D]2
Nub-GAL4, UAS-GCaMP6f	Brodskiy et al. (2019)	N/A	nub-GAL4,UAS-GCaMP6f/CyO
UAS-Rya-r44F RNAi	BDRC	31,540	y (1) v (1); P[y (+t7.7) v (+t1.8) = TRiP.JF01100 ]attP2
UAS-Insulin Receptor (dominant negative)	BDRC	8,248	y (1) w (1,118); P[w (+mC) = UAS-InR.del]2
UAS-Insulin Receptor (constitutively active)	BDRC	8,252	y (1) w (1,118); P[w (+mC) = UAS-InR.K1409A]2

**Table:** 

Fly lines	Source	Stock #
*D.ananassae*	*Drosophila* Species Stock Center	14,024–0,371.00
	University of California, San Diego	
*D. simulans*	*Drosophila* Species Stock Center	14,021–0,251.261
	University of California, San Diego	
*D. melanogaster*	Obtained from the lab of Dr. Fanis Missirlis, Cinvestav, Mexco	NA
*D. virilis*	*Drosophila* Species Stock Center. University of California, San Diego	15,010–1,051.87

## Data Availability

The datasets presented in this study can be found in online repositories. The names of the repository/repositories and accession number(s) can be found in the article/Supplementary Material.
