# Supplementary figures and images for: MAPPER: An Open-Source, High-Dimensional Image Analysis Pipeline Unmasks Differential Regulation of Drosophila Wing Features
Source: Front Genet. 2022 Apr 11;13:869719. doi: 10.3389/fgene.2022.869719 (PMC9035675; doi:10.3389/fgene.2022.869719)

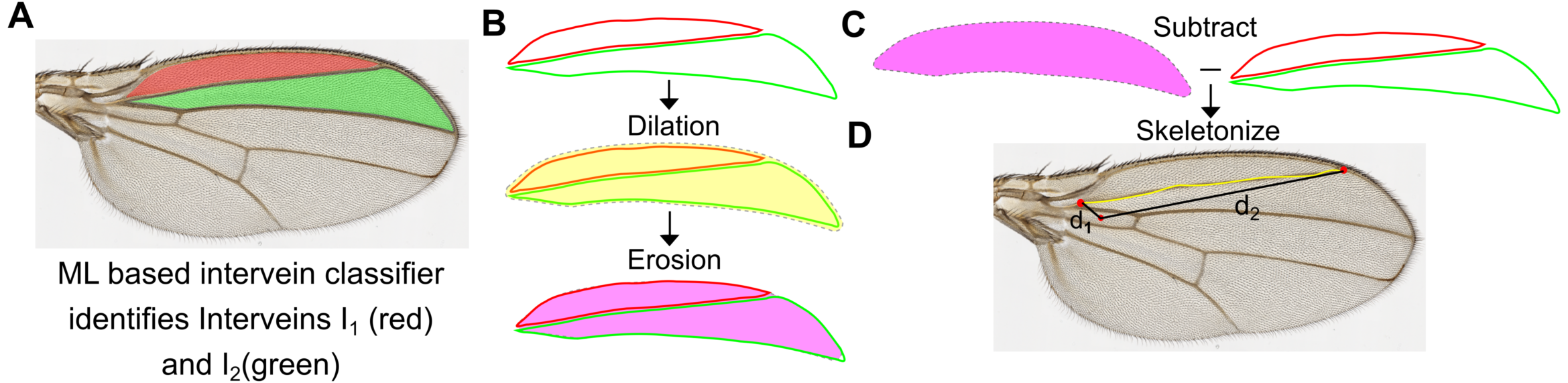

Supplement: Supplementary file 3 [file Image6.TIF]

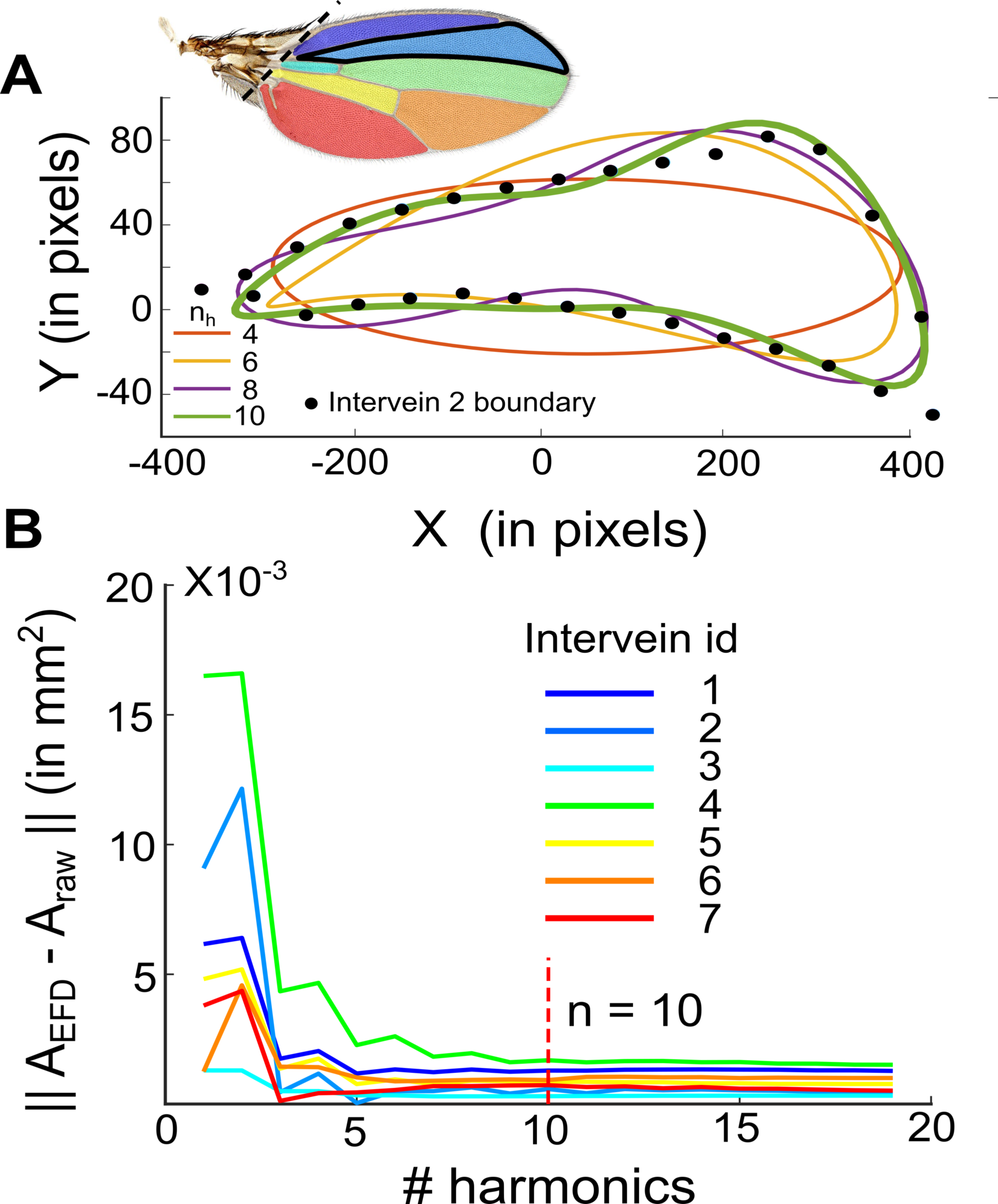

Supplement: Supplementary file 5 [file Image3.TIF]

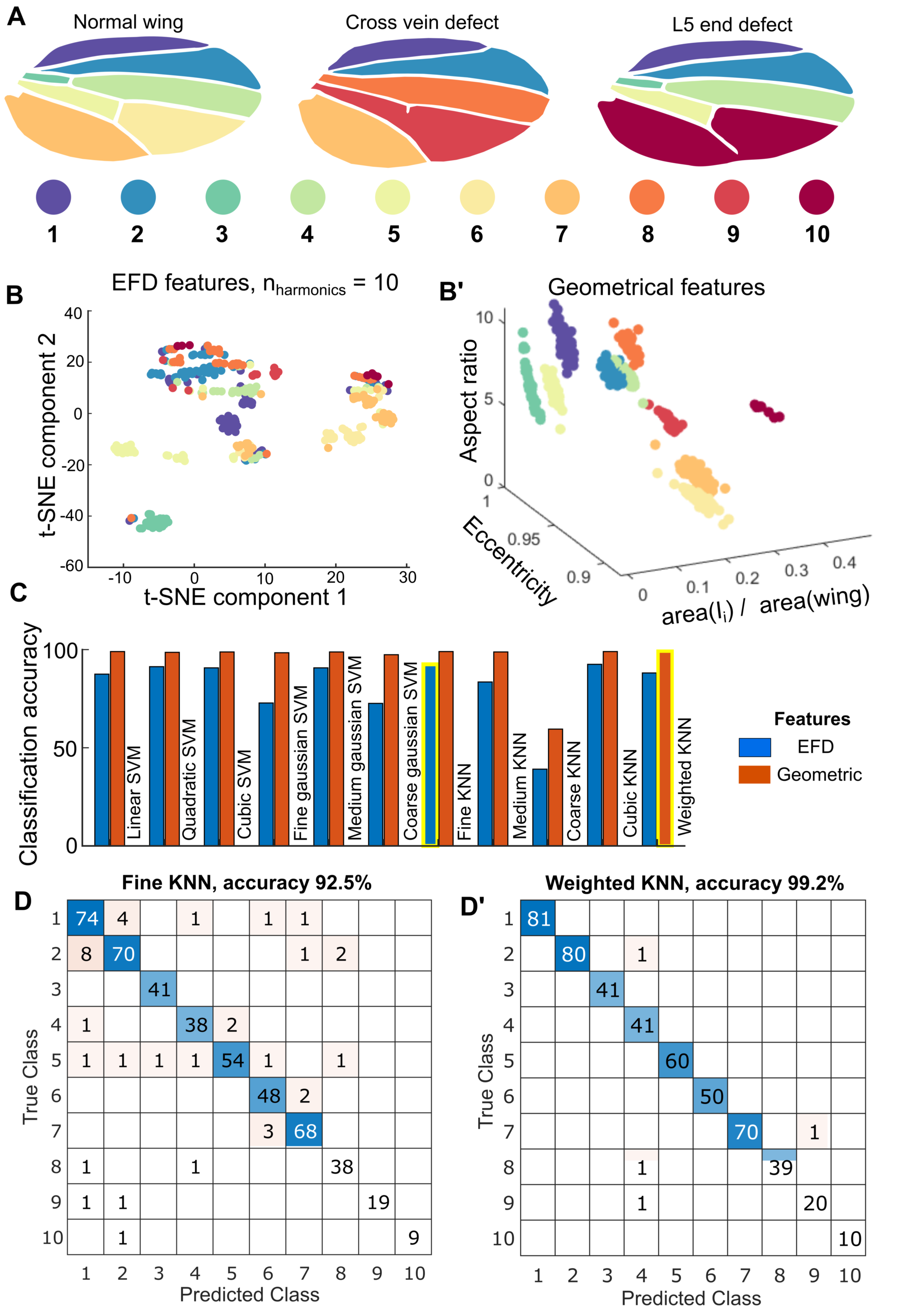

Supplement: Supplementary file 6 [file Image4.TIF]

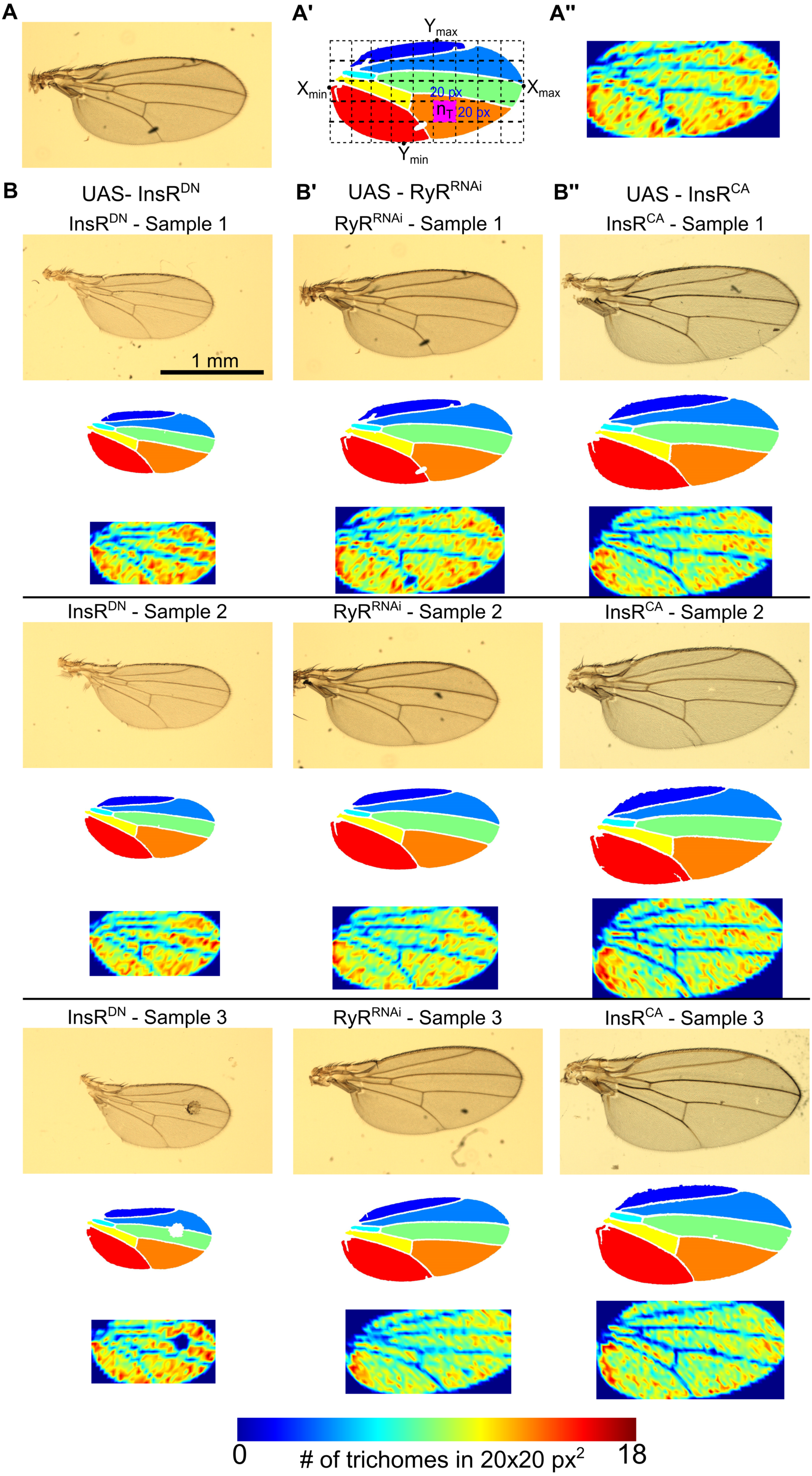

Supplement: Supplementary file 7 [file Image9.TIF]

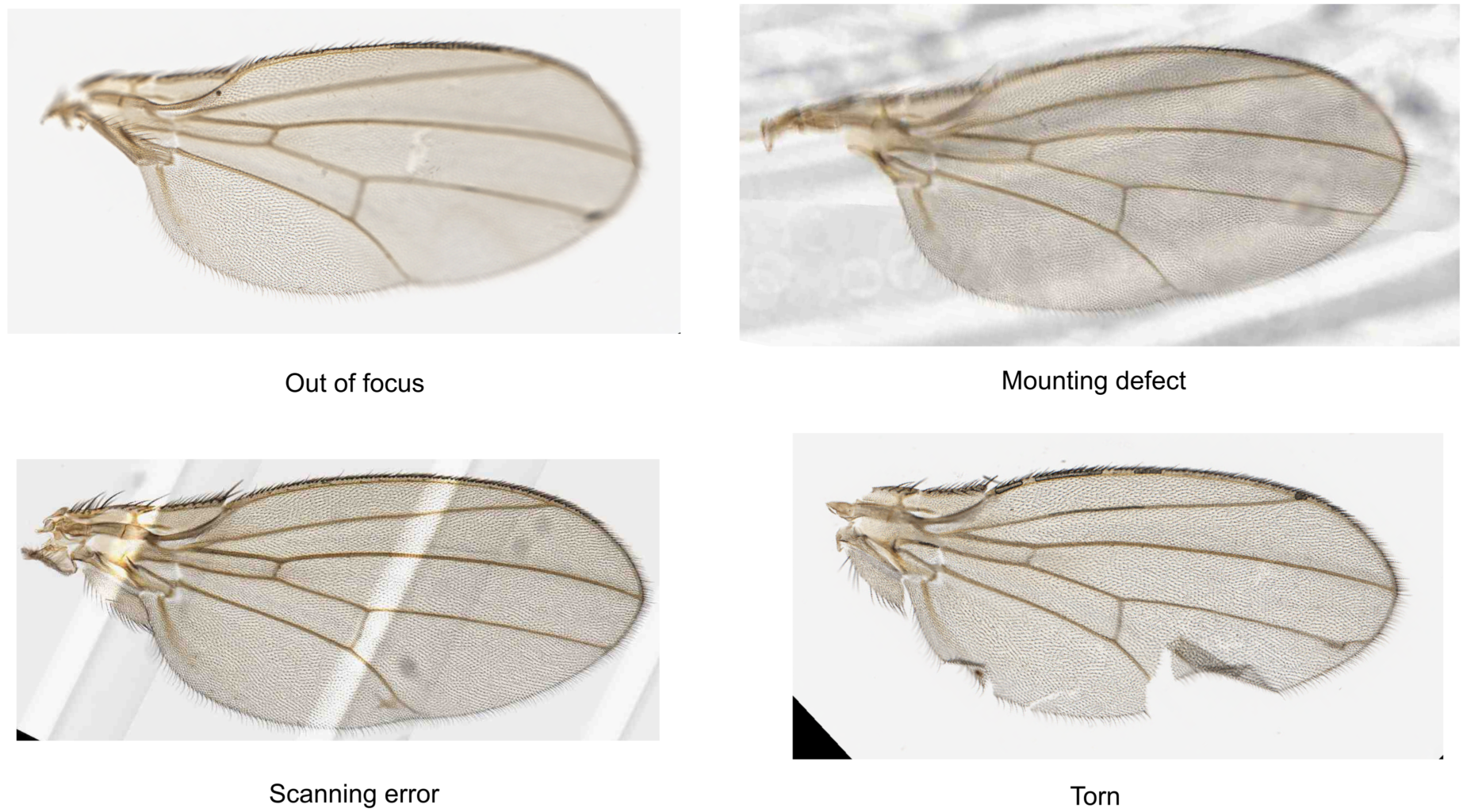

Supplement: Supplementary file 8 [file Image2.TIF]

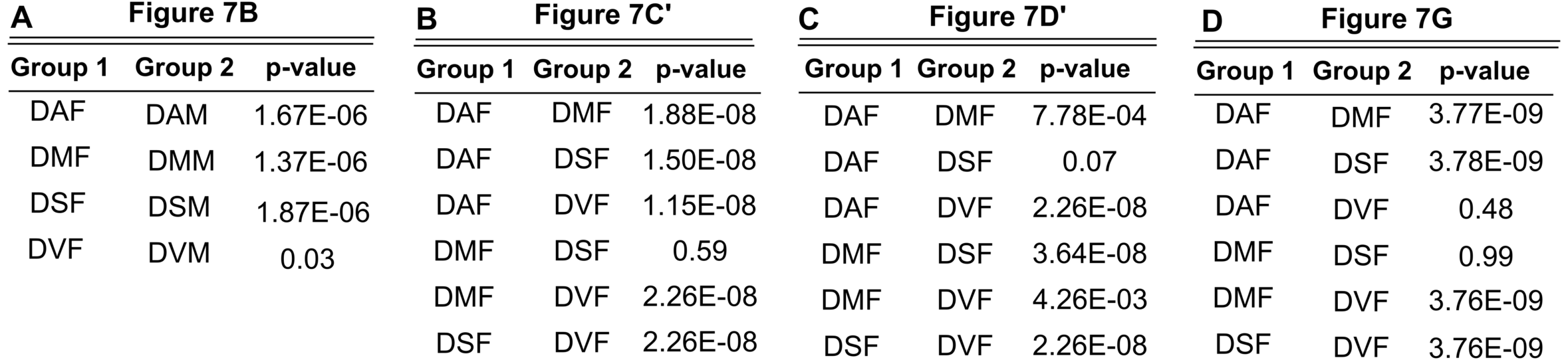

Supplement: Supplementary file 10 [file Image13.TIF]

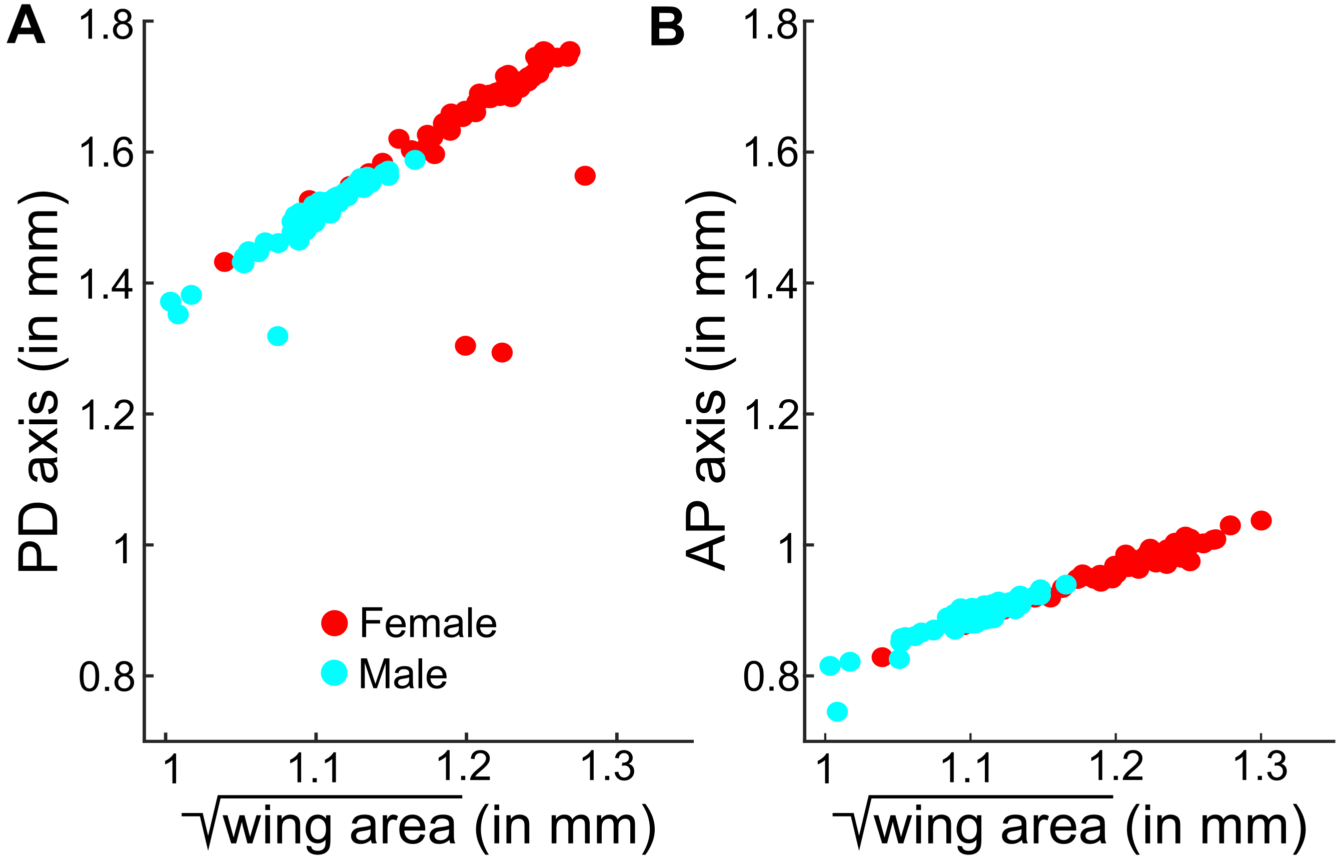

Supplement: Supplementary file 11 [file Image11.TIF]

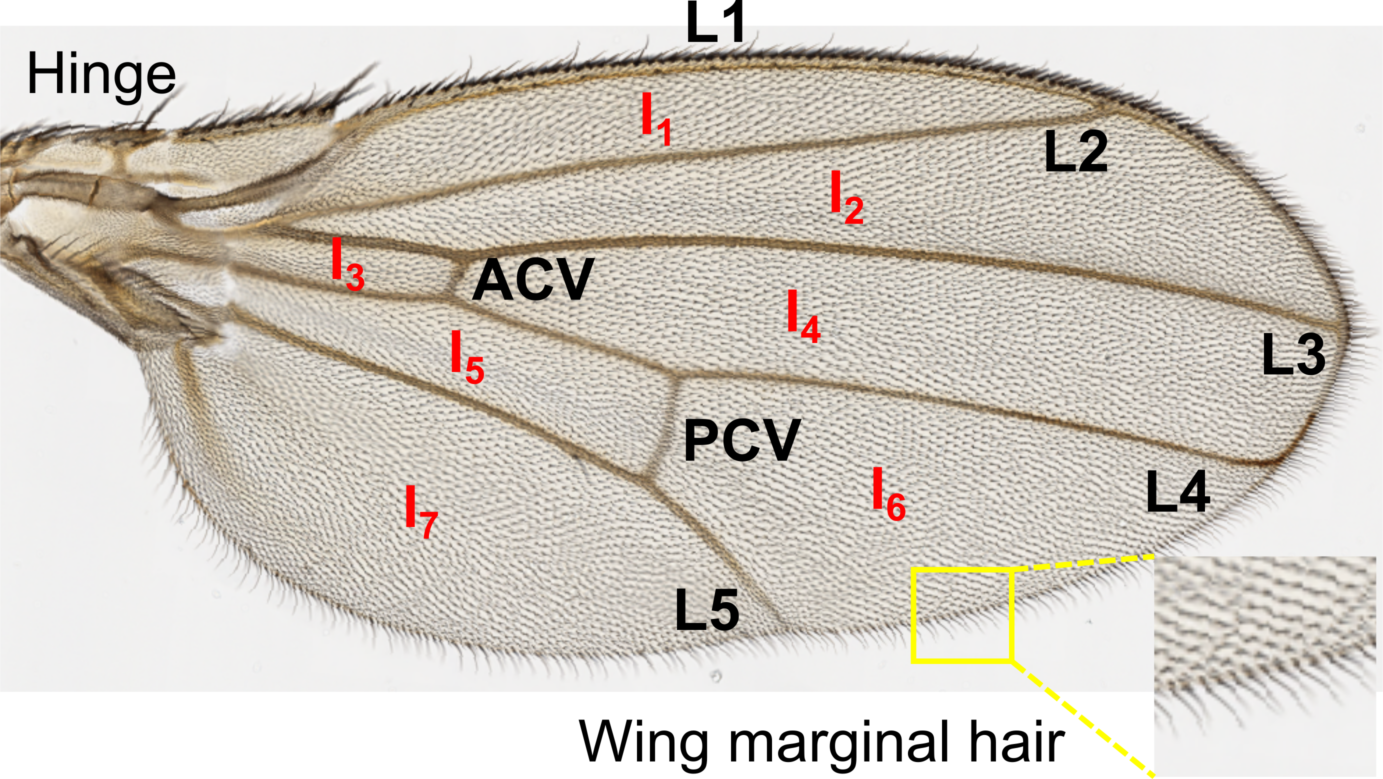

Supplement: Supplementary file 13 [file Image1.TIF]

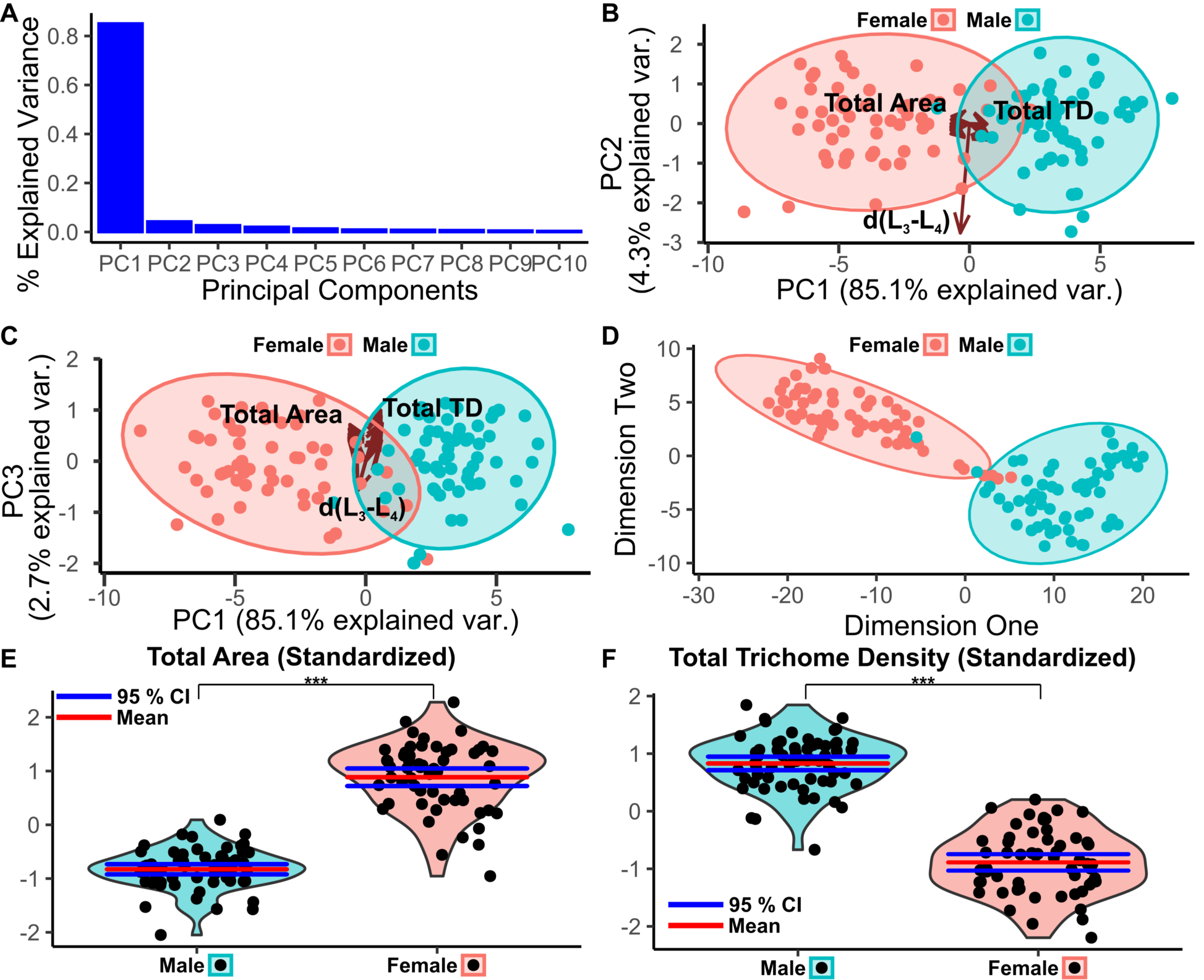

Supplement: Supplementary file 15 [file Image10.TIF]

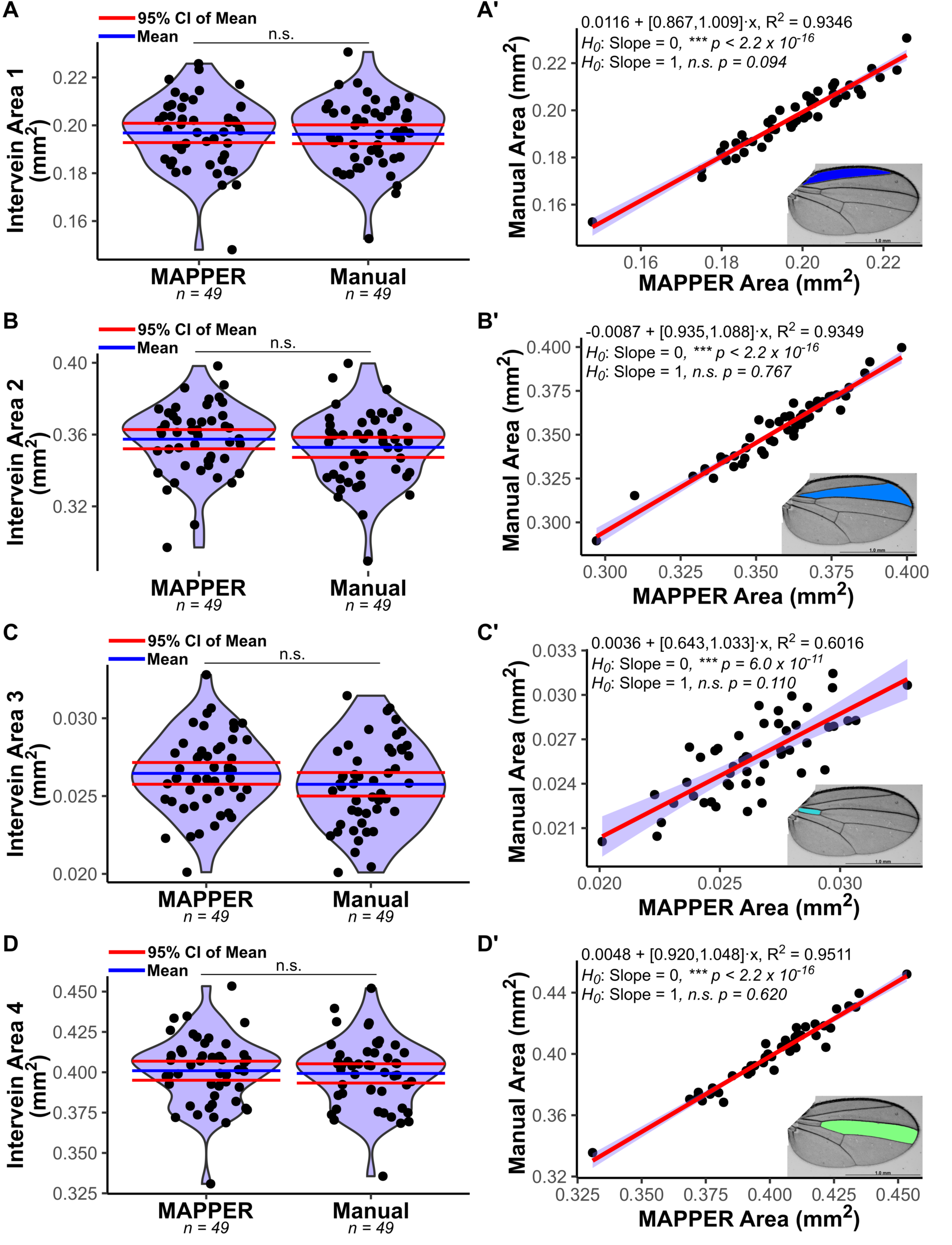

Supplement: Supplementary file 16 [file Image7.TIF]

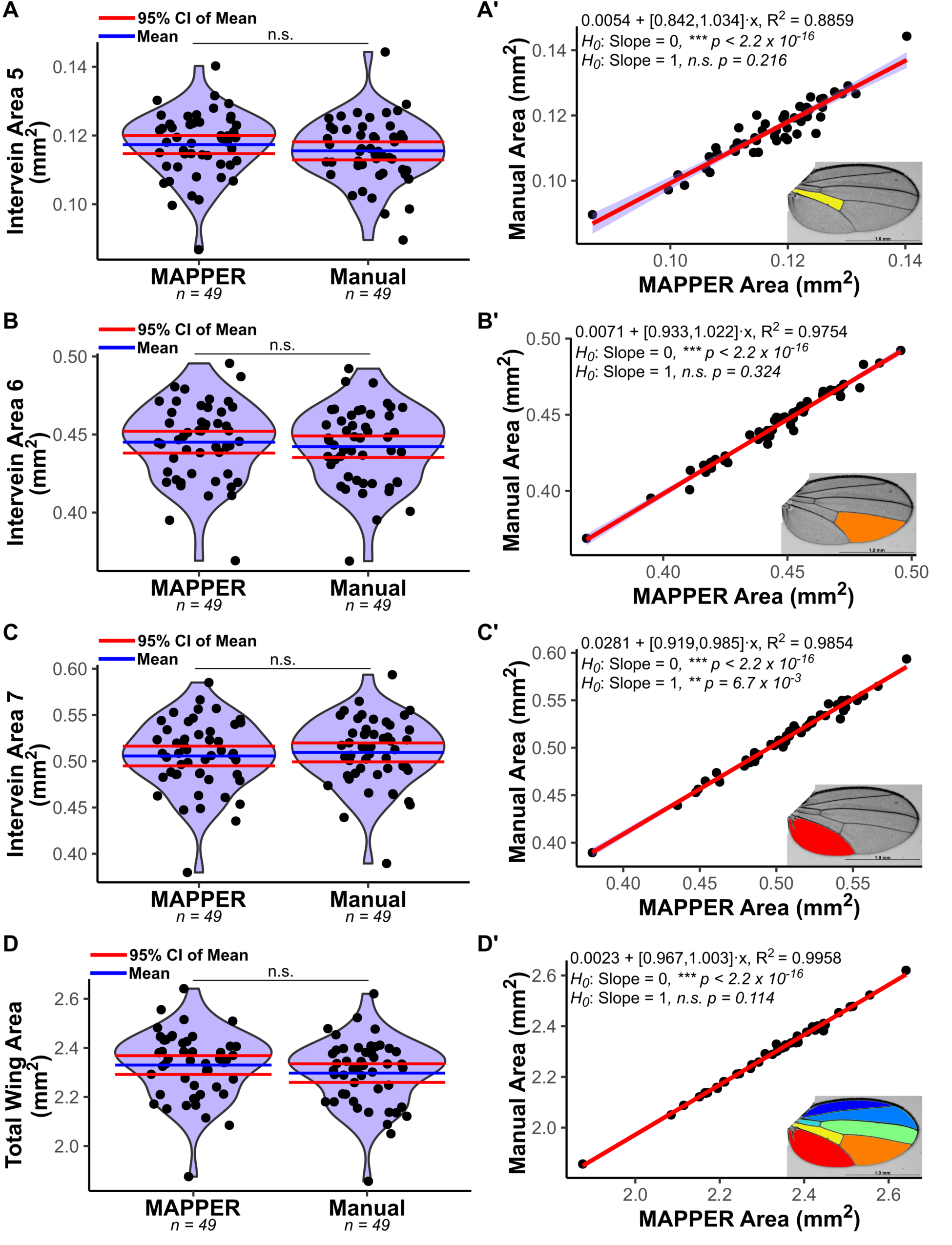

Supplement: Supplementary file 20 [file Image8.TIF]

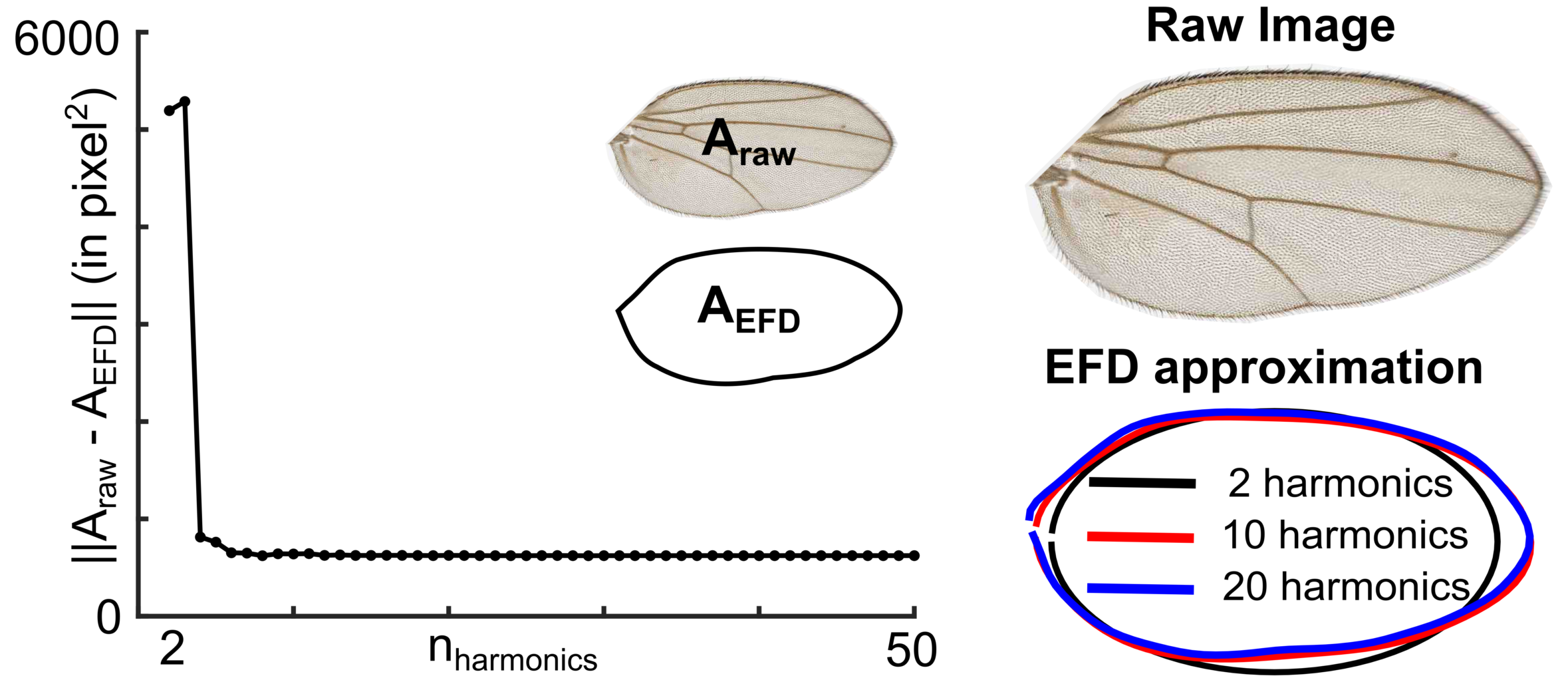

Supplement: Supplementary file 22 [file Image5.TIF]

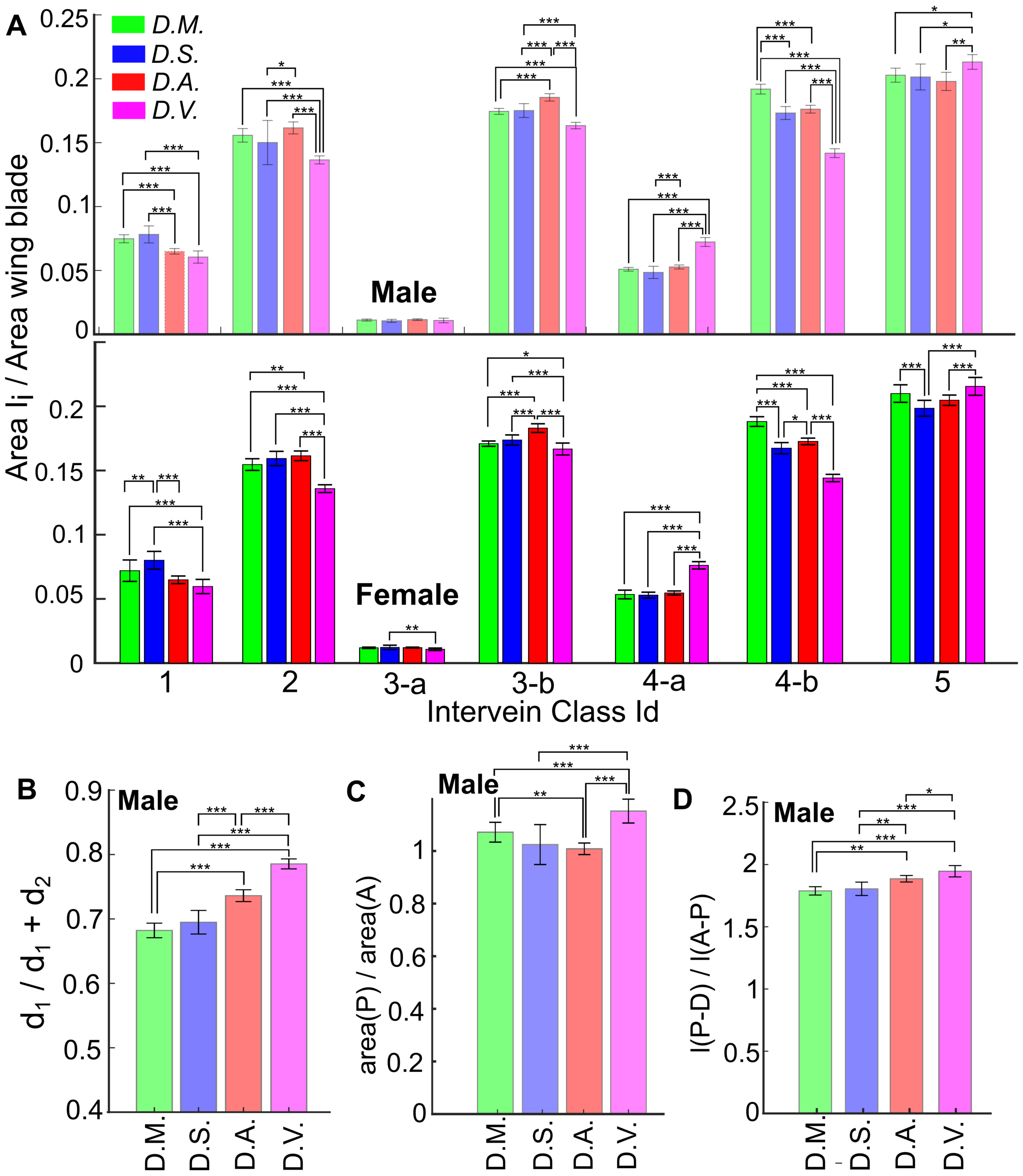

Supplement: Supplementary file 23 [file Image12.TIF]
