# Supplementary material for: MAPPER: An Open-Source, High-Dimensional Image Analysis Pipeline Unmasks Differential Regulation of Drosophila Wing Features
Source: Front Genet. 2022 Apr 11;13:869719. doi: 10.3389/fgene.2022.869719 (PMC9035675; doi:10.3389/fgene.2022.869719)
Supplement: Supplementary file 4 [file Presentation1.pdf]

MAPPER: An open-source, high-dimensional image analysis pipeline unmasks differential regulation of *Drosophila* wing features

Nilay Kumar[1,†], Francisco J. Huizar[1,†], Keity J. Farfán-Pira[2], Pavel A. Brodskiy[1], Dharsan K. Soundarrajan[1], Marcos Nahmad[2], Jeremiah J. Zartman[1,*]

[1]Department of Chemical and Biomolecular Engineering, University of Notre Dame, Notre Dame, IN 46556, USA

[2]Department of Physiology, Biophysics, and Neurosciences, Center for Research and Advanced Studies of the National Polytechnical Institute (Cinvestav), Mexico City, Mexico 07360.

[†]These authors contributed equally to this work.

*Correspondence: jzartman@nd.edu, (+1) 574-631-0455

Supplementary Information

Table of contents

S1 Deep learning-based image segmentation

- S1A U-Net training details: MAPPER utilizes U-Net for training
- S1B Deployment of a trained deep learning model on raw wing image data

S2 Training the intervein classifier

- S2A Generation of training data
- S2B Feature extraction for training
- S2C Training a machine learning-based intervein classifier

S3 Morphological feature extraction using the labelled interveins

- S3A Intervein geometric features
- S3B Trichome density of interveins
- S3C Landmark position-based features
  - S3C-I Length of proximal-distal axis
  - S3C-II Length of anterior-posterior axis
  - S3C-III Distance between veins L_3 and L_4
- S3D Elliptic Fourier Descriptors (EFD)

S4 App design and use

S5 Statistical analysis of *Drosophila melanogaster* Samarkand wings

S6 Tests for measuring statistical significance

S7 Code and data availability

S8 Supplementary figures

S9 References

S1 Deep learning based image segmentation

S1A. U-Net training details. MAPPER utilizes U-Net for training. U-Net (1) was trained using ground truth labels from 1000 raw wing images. The ground truth was generated using ILASTIK by first training a pixel classifier to identify the four pixel classes: vein, intervein, wing marginal hair, and background. The segmentation masks generated using ILASTIK were then used to train the U-Net. The output layer of a pre-trained U-Net that was earlier trained for biomedical image segmentation (2) was then modified to detect multiple classes. A cross entropy cost function was used (3). Training was carried out in PyTorch, and the implementation was done on GPUs. The raw images used for the training were imaged using an Aperio (Leica) slide scanner at 5X resolution.

S1B. Deployment of a trained deep learning model on raw wing image data. The trained model can be found in the linked GitHub repository.

S2 Training the intervein classifier

S2A. Generation of training data. MATLAB's Image labeler app was used to generate ground truth labels for training an intervein classifier. Adult wing images were imported into the application, and the polygon tool was used to define each intervein regions. A total of ten different classes were created that included the seven intervein regions in a normal adult wing blade, along with the cases of cross vein defects where either the anterior or posterior cross vein of the wings are missing (Figure S4A). A class where the L_5 vein fails to meet the margin of the wing blade, named as an L_5 end defect, has also been included. A total of 81 wing images belonging to five different classes present in the Supplementary S2 Training Resources folder were used for generating a total of 496 different objects belonging to the 10 intervein classes. The images used for labeling were marked and saved in the "trainingDataInterveinClassifier" subfolder. The five different classes and their folder names are:

- NormalPixelLabelDat
- ACVPixelLabelData
- PCVPixelLabelData
- ACVPCVPixelLabelData
- ACVEnddefectPixelLabelData.

S2B. Feature extraction for training. Two different kinds of feature extraction methodologies were followed while extracting features from different intervein components within the wing. The first one comprises basic geometric features. These include normalized intervein areas, eccentricity, and the aspect ratio of each intervein component. The feature vector for all the training data can be extracted by running the "ClassificationInterveinTraining.m" file. The feature vector is stored as an array where each row is a sample, and the three different columns contain the features. The labels for these are stored in the array named "ResponseTraining". Another methodology is when we used Elliptic Fourier Descriptor-based shape features (4). The number of features in this case depends on the number of harmonics to define an EFD model for the interveins. In our studies, we used a total of 15 EFD harmonics that resulted in a feature vector of size 496x(15x4).

S2C. Training a machine learning-based intervein classifier. The features extracted from the training data are imported to the MATLAB's classification learner app. All the models available were trained on the training data with a ten-fold cross-validation. Based on the geometric features, a weighted KNN performed the best, while based on the EFD features a Fine KNN was seen to have the most accurate classification (Figure S4C, Figure S4D). Both the models were saved for future use in intervein classification. Details on how we further choose the best model for our intervein classification has been described in the main text.

S3 Morphological feature extraction using the labelled interveins

S3A. Intervein geometric features. The machine learning-based intervein classification pipeline classifies and labels each intervein region. Then, MATLAB's regionprops tool extracts geometric features of each intervein. Geometric features like area, aspect ratio and circularity have been extracted and stored in the table containing wing features.

S3B. Trichome density of interveins. Trichomes are hairlike structures that are present in the *Drosophila* wing blade. The location where the base of the trichome originates is of darker intensity compared to the neighboring pixel. For each pixel present in the intervein region, we checked if it was a local minima in the defined window size containing the set of neighboring pixels. The window size was chosen as a circular patch of radius equaling half the length of a trichome. The same has been defined as a user input parameter as the length of hair in pixels varies with resolution of imaging. Once the location of trichomes is defined, we display the local trichome density using 2D histograms.

S3C. Landmark position-based features.

S3C-I. Length of proximal-distal axis. The calculation of length of proximal-distal axis requires definition of veins L_3 and L_4. Vein L_3 is reconstructed through morphological operations on I_2, I_3 and I_4 in MATLAB. Definitions of the interveins can be found in Figure S1. Interveins I_3 and I_4 are joined first by using dilating the boundary pixels and then by eroding by the same amount the joined components to form intervein $I_{3,4}$. In a similar way I_2 and $I_{3,4}$ are joined together to form $I_{2,3,4}$. I_2 and $I_{3,4}$ are then subtracted from $I_{2,3,4}$ to obtain the reconstruction of vein L_3. The same has been shown in Figure S6. We use a similar methodology to define vein L_4 as well. The veins are then skeletonized, and the vein endpoints are then extracted by locating pixels in the skeleton having maximum distance between them. The oriental of the wing allows identification of the proximal and distal ends of these veins. The average position of the proximal ends and distal ends is then

used to get the proximal-distal axis. Euclidean distance between the two points defines the length. (See Figure S6 for more details on how segmentation of veins are constructed).

S3C-II. Length of anterior-posterior axis. Specification of vein L_2 and L_4 is required to define the anterior-posterior axis of the wing blade. We follow a similar strategy as described above to define veins L_1 (I_1 and I_2) and L_4 (I_5, I_6, and I_7). The distal ends of veins L_2 and L_4 are then used to define the anterior-posterior axis of the wing.

S3C-III. Distance between veins L_3 and L_4. Distal ends of veins L_3 and L_4 are used to estimate the distance between veins L_3 and L_4.

S3D. Elliptic Fourier descriptors. EFDs have been used for a wide number of studies related to plant/leaf evolution and shape analysis of organs (4). We used Elliptic Fourier Descriptors (EFD) as an alternative for a robust translational and rotational invariant representation of wing shape. EFDs are found by fitting a Fourier series to the periodic function obtained from the closed *Drosophila* wing peripheral contour. The coefficients of the Fourier series act as features as each of them carry a local shape property. The original EFD description is a scale, translation, and rotation invariant shape descriptor (4). We modified the original algorithm, so it is sensitive to size changes. Details about this implementation can be found in the design section of the main text. To find the appropriate number of terms for representing the closed contour of the *Drosophila* wing blade, we varied the number of harmonics and measured the errors between the EFD reconstruction of the wing and the actual boundary points (Figure S5). Boundary points have been extracted from the U-Net/ILASTIK generated segmentation mask. With an increase in the number of terms, due to overfitting, the error decreases and saturates to 0. A further manual inspection showed that 20 harmonics are sufficient for representing any wing blade. In summary, EFDs allow us to measure specific local changes within the wing corresponding to a genetic or a pharmacological perturbation.

S4 App design and use

A full documentation and user manual of MAPPER is located on [GitHub here](). Within the user manual are guidelines for file formats, folder organization, image pre-processing, input parameter specifications, user operations, troubleshooting inquiries, and understanding MAPPER's output.

S5 Statistical Analysis of Samarkand strain *Drosophila* wings

We processed 128 adult wing images of *Drosophila melanogaster* from the Samarkand strain (Figure 5, Figure S10) (5). In this validation test, MAPPER was used to highlight the shape and size differences between the male and female populations. Geometric features and EFD features were separately analyzed to highlight the application of EFD in estimating local shape changes in wing. Principal Component Analysis (PCA) (6) carried out on the geometric features revealed that the maximum variance within data was distributed majorly between the first two principal components (89.4%) (Figure 5D, Figure S10A, S10B). PCA also revealed that the total area of the wing and total trichome density had maximum loading towards Principal Component 1 (PC1) with total wing area having a negative PC1 loading towards the female population, and total trichome density having a positive PC1 loading towards the male population (Figure S10B, S10C). Interestingly, the distance between longitudinal veins L_3 and L_4, d(L_3-L_4), did not correlate with other wing features for either population (Figure S10B). Further, d(L_3-L_4) did not have positive or negative loading for PC1, which explained most of the variance in the data. This is contrary to the other biological axes measurements for the AP and PD lengths that had negative PC1 loading towards the female population. The differences between males and females were further characterized by plotting the known result that wings of a *Drosophila* female are greater in size than the male wing (Figure 5E, Figure S10E). PCA analysis and comparison of the standardized total wing areas confirmed this ($p < 0.001$). Conversely, males had a larger total standardized trichome density (Figure S10F, $p < 0.001$) that is revealed by direct comparison and PCA analysis.

Taken together, this suggests that high-dimensional data provided by MAPPER can reveal unique features that are disparate between populations using PCA analysis. Clustering carried out using the first two principal components revealed the presence of two distinct clusters representing the male and female populations (Figure 5D). Further, performing t-distributed stochastic neighbor embedding (t-SNE) analysis revealed similar clustering distinctions between male and female populations (Figure S10D). High-dimensional analyses, such as PCA and tSNE, allow for a systematic screening of phenotypic changes between two different populations, and can even be extended to multiple populations.

A similar approach was followed in analysis of the EFD-based features. PCA was applied on the EFD coefficients extracted for each wing. In this case, a total variance of about 97% was captured in PC1 alone (Figure 5F). This indicates that the variation was mainly distributed in one direction of linear combinations of the EFD coefficients. Thus, the high-dimensional output of MAPPER coupled with PCA analysis can reveal wing shape features that are distinct between two populations. To investigate the importance of PC1 on wing geometry, we implemented reverse PCA on the principal components. Reverse PCA is carried out by adding the mean vector of the features to the matrix product of PCA projections (scores) and the transpose of the eigenvectors (Equation 1). This process enables mapping of the influence of principal components back to the original data.

$$PCA_{Reconstructed} = PCA_{Scores} \cdot Eigenvectors^{T} + \mathrm{X}_{\mathrm{features}} \quad (1)$$

In our analysis, the first eleven principal components were used to reconstruct the EFD coefficients via reverse PCA. Since we are interested in understanding the importance of PC1, which explained most of the variance in the data, the standard deviation along this PC was calculated. Mean scores of the first 11 PCs were then used to reconstruct a representative wing for the male and female populations (Figure 5G). 1.5 times the calculated standard deviation

along PC1 was then added and subtracted to the representative wing to observe the effect of PC1 on wing shape. Reverse PCA was then used to approximate the EFD coefficients with varying PC1. Reverse EFD was then carried out to estimate the contour of the wing blade. The reconstructed contours highlight that the major differences between the wings is mainly because of overall change in the wing blade area (Figure 5H). However, the reconstructed contour can be used as the mean representation of the samples. This approach is useful when analyzing subtle changes in shapes resulting from dysregulations in genetic pathways. These subtle differences are further characterized by clustering using Gaussian Mixture Models (7) (GMM) where the presence of two distinct clusters is revealed (Figure 5F). Both of these high-dimensional plots reveal that there are unique and identifiable distinctions between male and female populations in regard to wing shape.

S6 Tests for measuring statistical significance

One way analysis of variance (ANOVA) was first used to test the hypothesis if the means of the groups compared were equal or not (8). However, ANOVA alone cannot be used to comment on statistical significance of comparisons between any two subgroups. Therefore, we further used a multiple group comparison test for the statistics generated by ANOVA. Tukey's honestly significant difference procedure was used for this task to identify differences in means among all subgroups (9). Bonferoni-Holm correction was then applied on the generated *p*-values from the previous test in order to adjust for Type I error in statistical testing (10). All the steps were carried out using MATLAB. Additionally, a Bartlett test was used to compare the variances between any of populations.

S7 Code and data availability

The application, its source code, and all data spreadsheets used for the analysis can be found on the application's GitHub page here.

S8 Supplementary figures

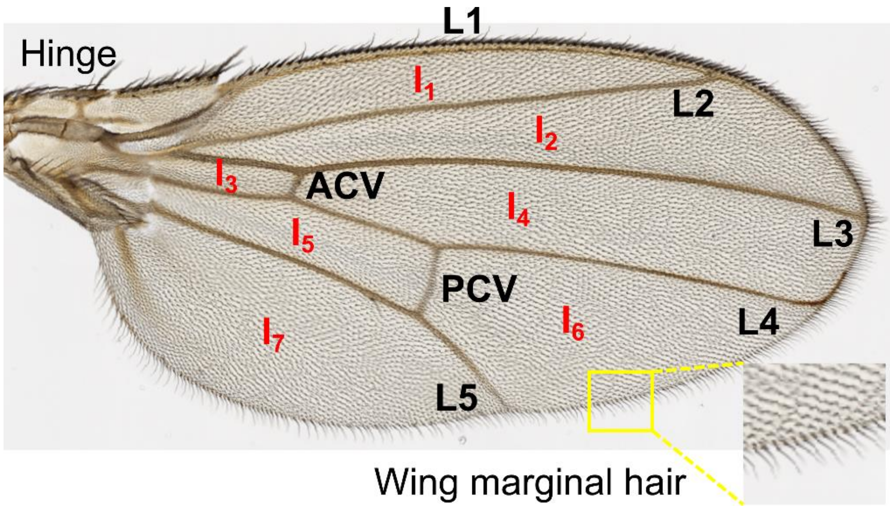

Figure S1. Components of adult *Drosophila* wing blade. The adult *Drosophila* wildtype wing has distinct morphological features: longitudinal veins L_1-L_5, anterior cross vein (ACV), and the posterior cross vein (PCV), that we rely on to study the effect of specific genetic perturbation on morphogenesis. Veins also enclose seven specific intervein regions (I_1 - I_7). The periphery of the wing is surrounded by marginal hairs. The intervein regions and veins within the periphery are also patterned with small hair like structures, called trichomes. The wing is attached to the fly body through a hinge.

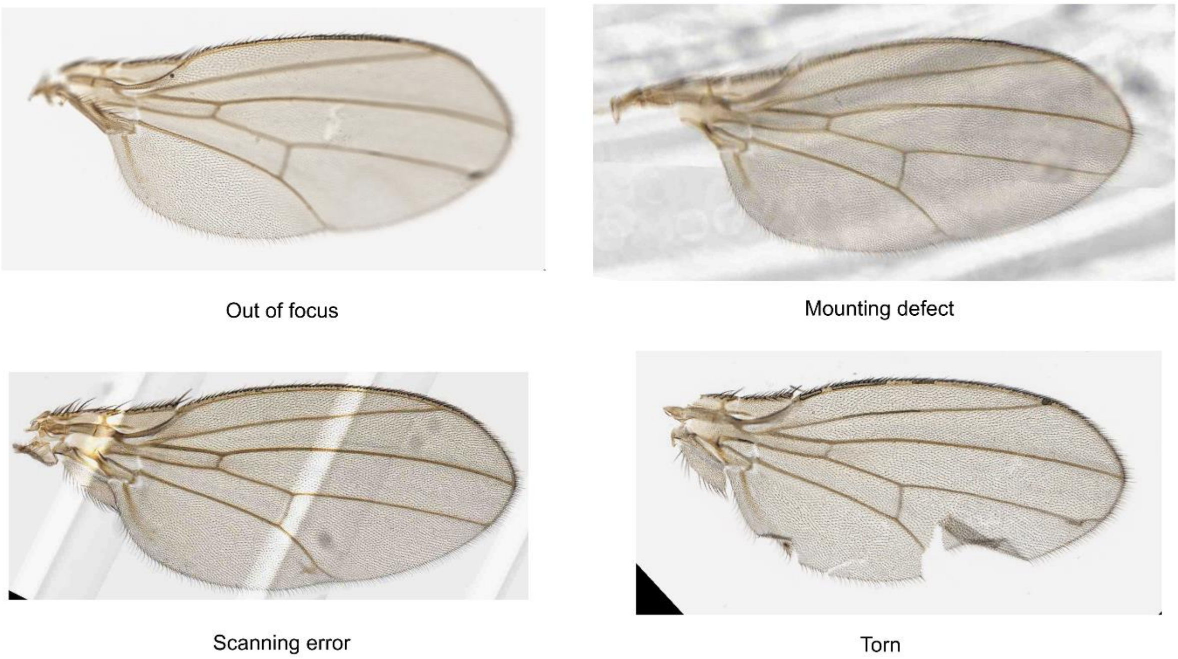

Figure S2. Defective wing images that are excluded from the pipeline. Shown here are examples of wing images that contain various image artifacts or sample preparation defects. The MAPPER pipeline is able to recognize these defects and remove the corresponding images from the analysis.

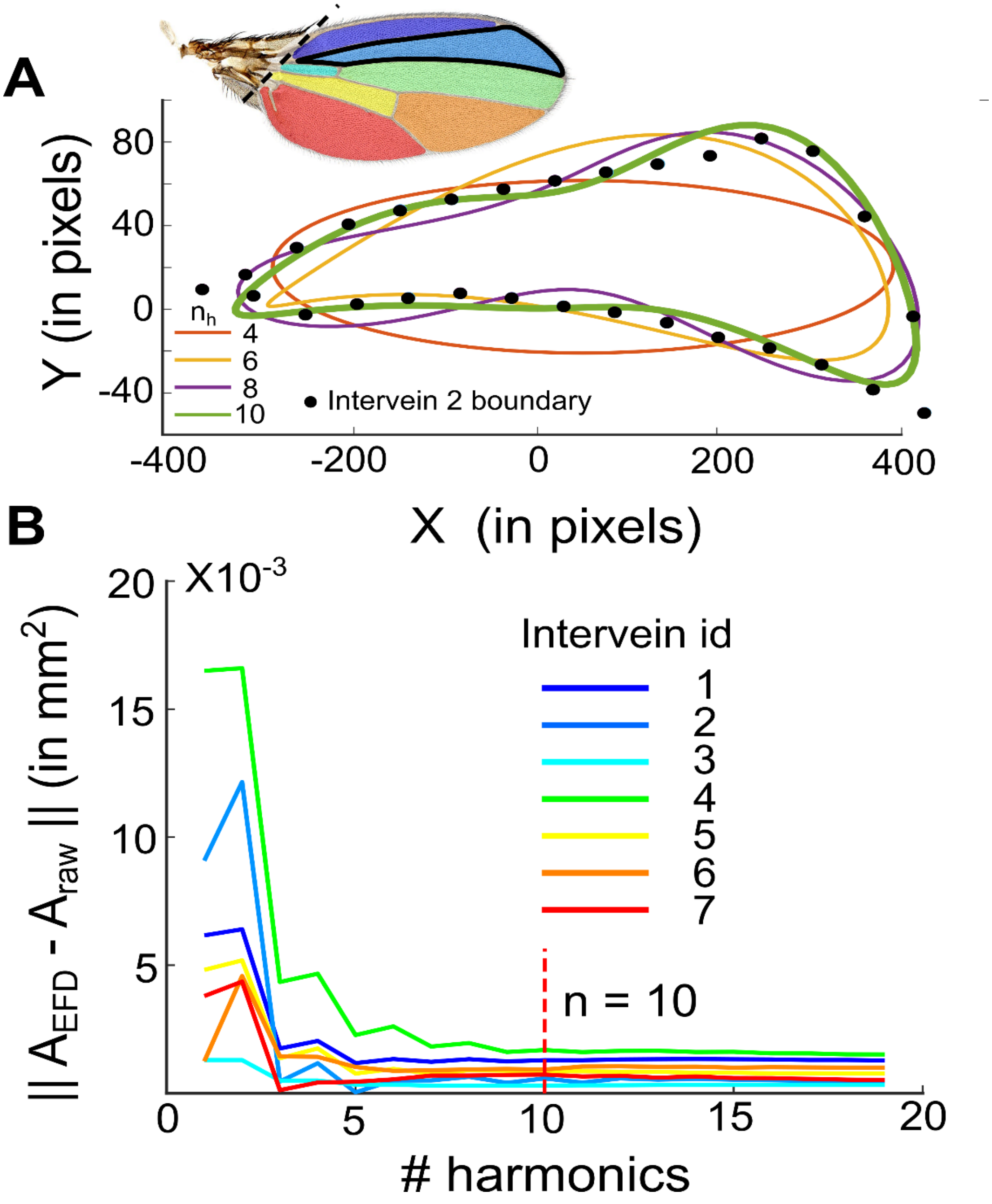

Figure S3. A total of ten terms in EFD description is sufficient to define the geometry of an intervein. **(A)** Variation of number of terms in the elliptic Fourier descriptors (EFDs) on accuracy of fit for specifying the intervein enclosed between veins L_2 and L_3 (I_2). As the number of terms increases, the EFD fit approaches the true intervein shape. **(B)** Variation in error with an increase in number of terms in the EFD description for each intervein. The error is approximated by calculating the absolute difference between area enclosed by the EFD fit and actual intervein area.

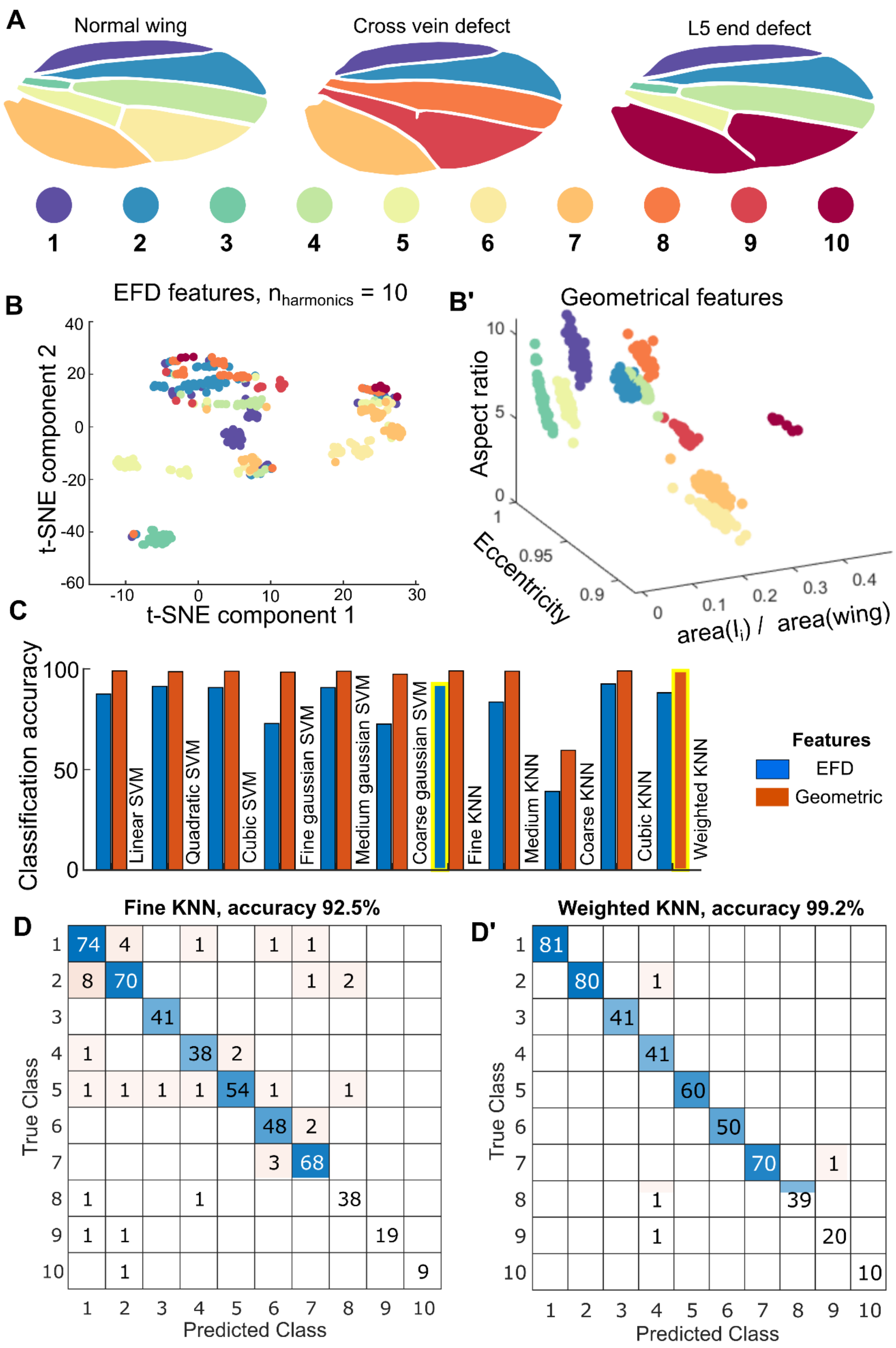

Figure S4. KNN trained on geometric features performs best for intervein classification. (A) Definition of each intervein region used while creating the training data for the purpose of intervein classification. Vein

defects led to additional intervein classification regions. **(B)** t-SNE representation of EFD features extracted from each individual intervein region from a batch of wing images used for generating the training dataset. Clear clustering is present in the different intervein regions. **(B')** Geometric features such as normalized intervein area, eccentricity, and aspect ratio are also used for training an alternate intervein classifier. **(C)** Performance of different support vector machines (SVMs) and k-nearest neighbor algorithms (KNNs) present in MATLABs classification learner application based on the two different sets of features used for the purpose of training. Weighted KNN performed the best compared to other methods. **(D-D')** Confusion matrices for best models based on the two different sets of features used. (D) uses the EFD-based features while (D') uses the geometrical features of individual intervein.

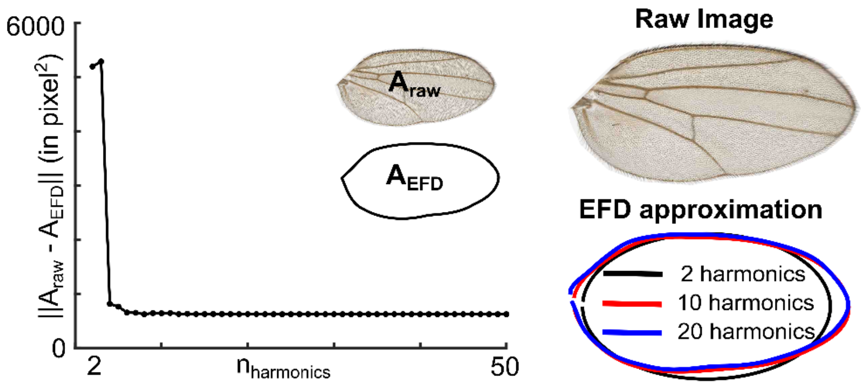

Figure S5. EFD description of the global wing shape. Elliptic Fourier Descriptor (EFD) coefficients are determined to fit the perimeter of the adult wing blade. Left-hand side graph shows the variation of error with an increase in the number of harmonics, or the terms used in EFD approximation. Error is approximated by calculating the absolute differences between the actual area of the wing and the area enclosed by an EFD fit. Right hand side panel shows the actual representation of the effect of increasing the number of harmonics on overall fit.

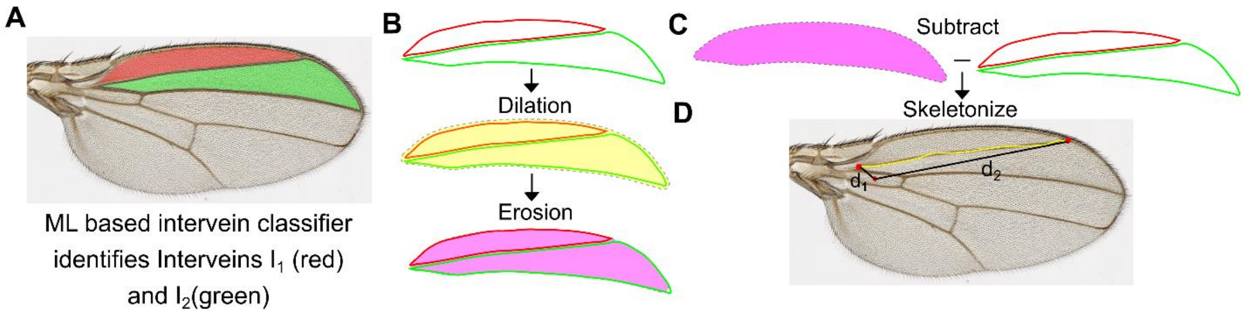

Figure S6. Methodology for extracting proximal and distal ends of longitudinal vein L_2. (A) The intervein classification pipeline identifies intervein region I_1 and I_2. **(B)** Binary masks for intervein regions I_1 and I_2 are dilated in order to fuse them together. A disc structuring element with radius equaling vein thickness was used for this. The dilated image is then eroded by the same structuring element. **(C)** The binary masks for I_1 and I_2 are then subtracted from the binary mask obtained after dilation and erosion, and the resulting mask is then skeletonized to obtain the definition of vein L_2. The distance of the ends of the vein are calculated from the center of intervein region I_3 to estimate the proximal and distal ends of vein (Figure S1A).

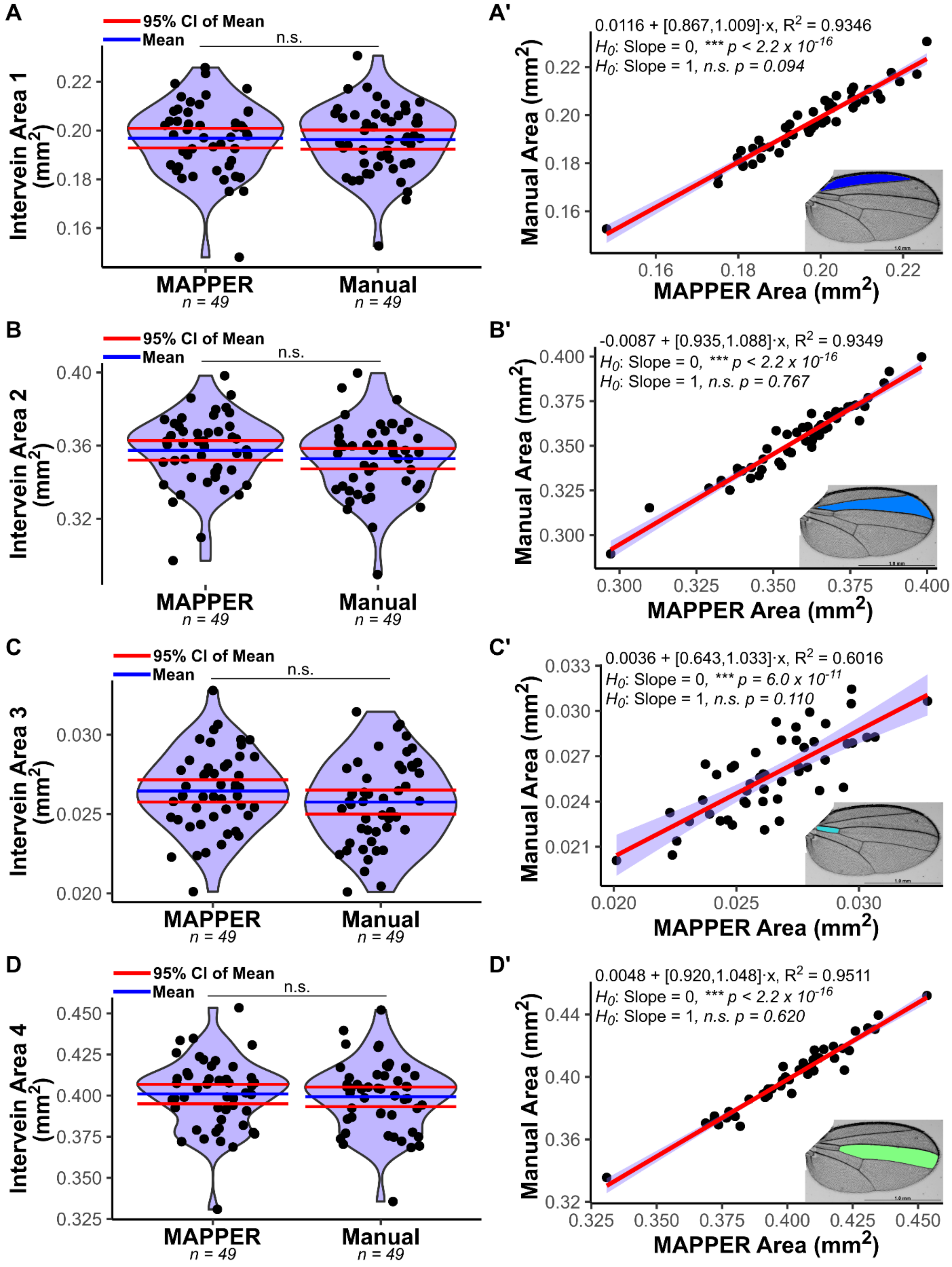

Figure S7. MAPPER automated measurements have a one-to-one correspondence with manual hand measurements for intervein regions 1—4. To validate MAPPER's automated measurements, manual measurements were manually taken and compared to MAPPER's output for four intervein regions

areas of the wing: 1) Area between the 1st and 2nd longitudinal veins, 2) Area between the 2nd and 3rd longitudinal veins, 3) Area between the 3rd and 4th longitudinal veins on the proximal side of the anterior cross vein (ACV), and 4) Area between the 3rd and 4th longitudinal veins on the distal side of the ACV. Data for all panels were generated from female Samarkand species *Drosophila* from publicly available images (5). In each pair of panels, denoted by a letter character and the same apostrophized letter character, are violin distribution plots of the automated and hand measurements (left) and linear regression fits of automated versus manual measurements (right). Fit linear regression models are overlaid as red lines and light-blue bands correspond to the 95% CI of the fit. Inset *Drosophila* wings with color-coded regions depict the corresponding region being measured. F-tests (11) were performed to compare variances of the distributions, and unpaired T-tests (12) were performed to compare means of the distributions. For all F-tests and t-tests *p*-values above 0.05, it is indicative of statistically identical variances and means, respectively. **(A)** Intervein area 1 manual and automated measurements were compared. F-test (p = 0.836) and an unpaired T-test (p = 0.846). **(A')** The slope parameter of the fit was found to not be statistically different from a value of 1.00 (p = 0.094). **(B)** Intervein area 2 manual and automated measurements were compared. An F-test (p = 0.756) and an unpaired T-test (p = 0.254). **(B')** The slope parameter of the fit was found to not be statistically different from a value of 1.00 (p = 0.767). **(C)** Intervein area 3 manual and automated measurements were compared. An F-test (p = 0.594) and an unpaired T-test (p = 0.184). **(C')** The slope parameter of the fit was found to not be statistically different from a value of 1.00 (p = 0.110). **(D)** Intervein area 4 manual and automated measurements were compared. An F-test (p = 0.952) and an unpaired T-test (p = 0.695). **(D')** The slope parameter of the fit was found to not be statistically different from a value of 1.00 (p = 0.620).

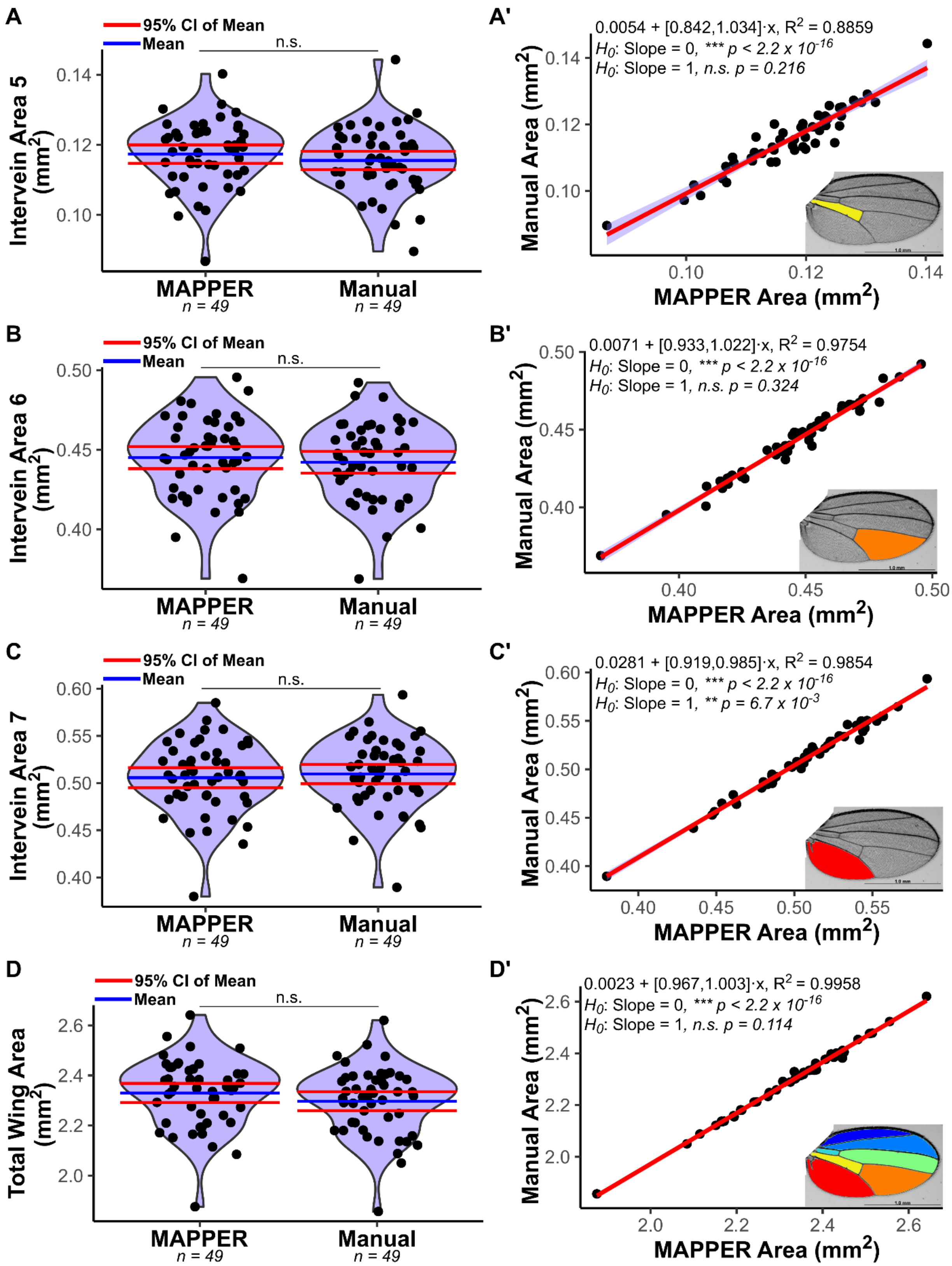

Figure S8. MAPPER automated measurements have a one-to-one correspondence with manual hand measurements for intervein regions 5—7 and total wing area. To validate MAPPER's automated

measurements, manual measurements were manually taken and compared to MAPPER's output for three intervein regions areas of the wing and the total wing area: 5) Area between the 4th and 5th longitudinal veins on the proximal side of the anterior cross vein (ACV), 6) Area between the 4th and 5th longitudinal veins on the distal side of the ACV, 7) Area between the 5th longitudinal vein and wing periphery. Data for all panels were generated from female Samarkand species *Drosophila* from publicly available images (5). In each pair of panels, denoted by a letter character and the same apostrophized letter character, are violin distribution plots of the automated and hand measurements (left) and linear regression fits of automated versus manual measurements (right). Fit linear regression models are overlaid as red lines and light-blue bands correspond to the 95% CI of the fit. Inset *Drosophila* wings with color-coded regions depict the corresponding region being measured. F-tests (11) were performed to compare variances of the distributions, and unpaired T-tests (12) were performed to compare means of the distributions. For all F-tests and t-tests *p*-values above 0.05, it is indicative of statistically identical variances and means, respectively. **(A)** Intervein area 5 manual and automated measurements were compared. An F-test (p = 0.983) and an unpaired T-test (p = 0.342). **(A')** The slope parameter of the fit was found to not be statistically different from a value of 1.00 (p = 0.216). **(B)** Intervein area 6 manual and automated measurements were compared. An F-test (p = 0.943) and an unpaired T-test (p = 0.561). **(B')** The slope parameter of the fit was found to not be statistically different from a value of 1.00 (p = 0.324). **(C)** Intervein area 7 manual and automated measurements were compared. An F-test (p = 0.773) and an unpaired T-test (p = 0.607). **(C')** The slope parameter of the fit was found to be statistically different from a value of 1.00 ($p = 6.7 \times 10^{-3}$). The 95% CI of the slope parameter is [0.919, 0.985]. **(D)** Total wing area manual and automated measurements were compared. An F-test (p = 0.928) and an unpaired T-test (p = 0.236). **(D')** The slope parameter of the fit was found to not be statistically different from a value of 1.00 (p = 0.114).

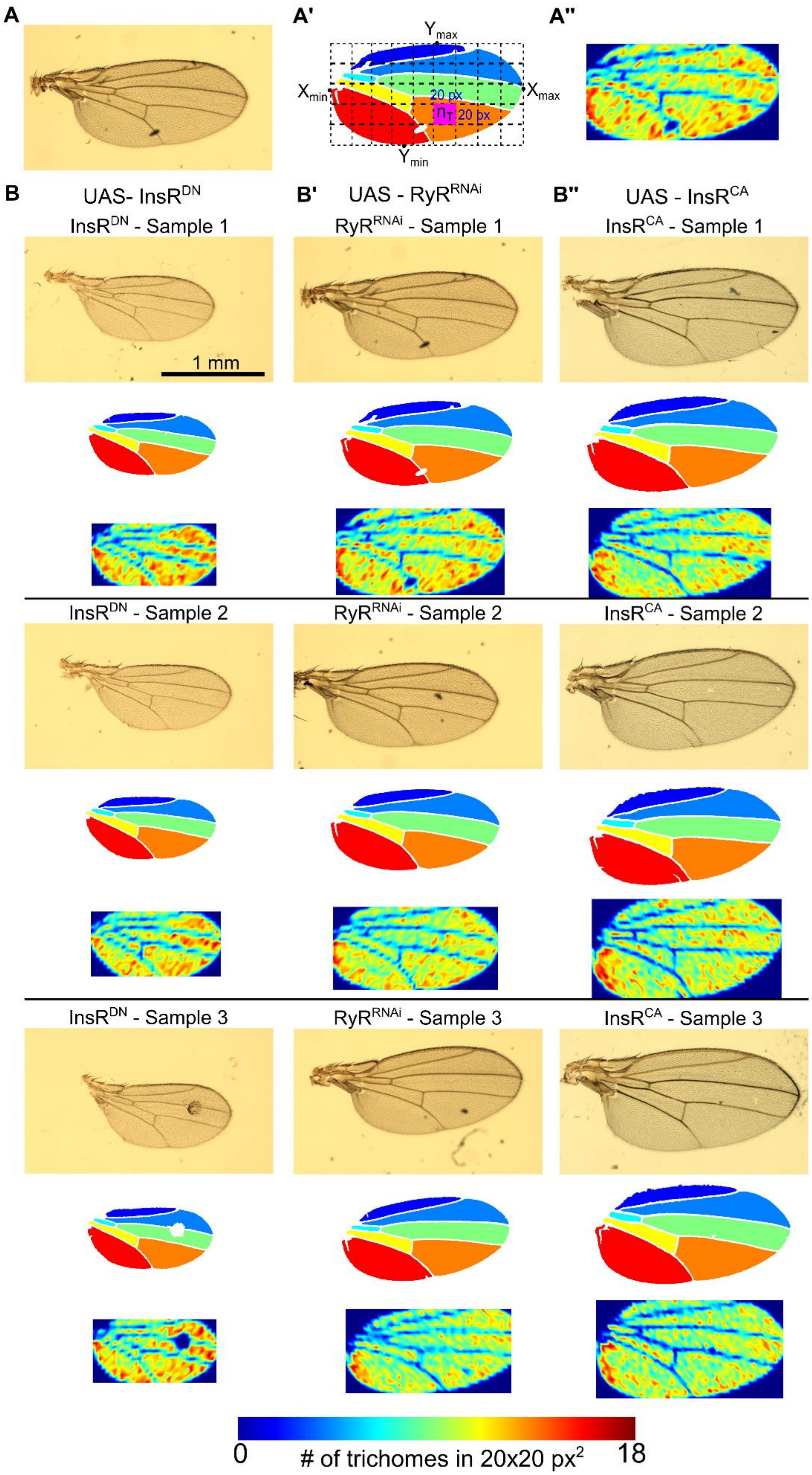
A
A'
Y_{max}
X_{min}
X_{max}
Y_{min}
20 px
n_T
20 px
A''
B
UAS- $InsR^{DN}$
B'
UAS - RyR^{RNAi}
B''
UAS - $InsR^{CA}$
$InsR^{DN}$ - Sample 1
RyR^{RNAi} - Sample 1
$InsR^{CA}$ - Sample 1
1 mm
$InsR^{DN}$ - Sample 2
RyR^{RNAi} - Sample 2
$InsR^{CA}$ - Sample 2
$InsR^{DN}$ - Sample 3
RyR^{RNAi} - Sample 3
$InsR^{CA}$ - Sample 3
0
# of trichomes in 20x20 px^2
18

309

Figure S9. Trichome density is regulated by Insulin signaling. (A-A'') MAPPER is first used to estimate the location of trichomes within each individual labelled intervein region. The extreme coordinates of the intervein regions are then used to draw a bounding box around the wing. The bounding box is next binned into subregions of size 20 x 20 $pixel^2$ area. The number of trichomes in each subregion is then estimated and the "densityplot" function (13) is used to construct a heatmap highlighting the local density of trichomes within the wing. **(B-B")** Three representative wing samples from perturbed Insulin signaling pathways were selected. Along with the raw image, MAPPER generated colored, labelled intervein regions along with the trichome density heatmaps. Genotypes corresponding to each perturbation are described at the top of figure. The color bar describes the number of trichomes in 20 x 20 $pixel^2$ area subregions of the wing.

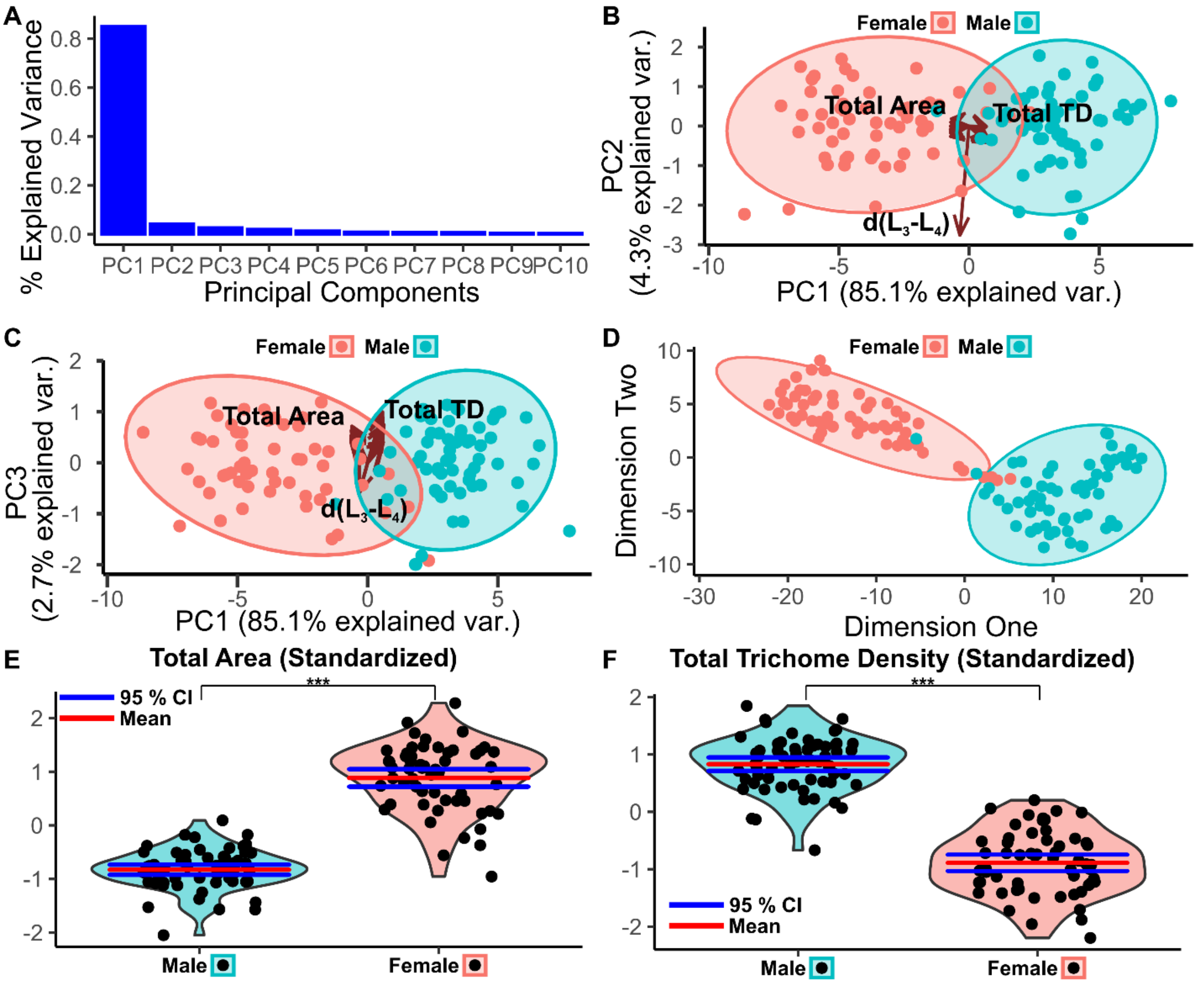

Figure S10. MAPPER provides high-dimensional analysis capabilities to identify unique morphometric features. *Drosophila* adult wings from Samarkand strain males and females were analyzed using MAPPER's batch processing capabilities (5). **(A, B, C)** Principal component analysis (PCA) on centered and scaled wing features revealed most of the variance in the data is explained by PC1, with distinct clustering across PC1 between males and females. Arrows represent projection of eigenvectors for each feature, and ellipses are 95% confidence intervals (CI) of the data populations. **(D)** t-SNE analysis on wing features revealed distinct clustering between males and females. **(E, F)** Violin plots of standardized wing features identified in PC analysis. means (red) and 95% CIs (blue) means (red) are overlaid for comparison. (*** *p*-value < 0.001 via Mann-Whitney U Test).

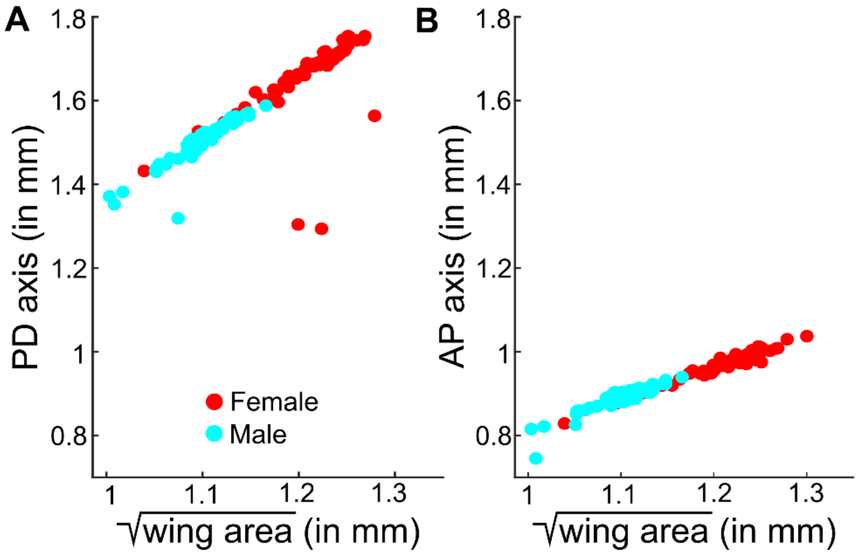

Figure S11. AP axis and PD axes scale uniformly with respect to area of the wing. Analysis was carried out for a population of male and female wings belonging to *Drosophila melanogaster* Samarkand strain. The points are color coded according to sex where red indicates female and blue the male population of wings. **(A, B)** Variation of proximal-distal axis and anterior-posterior axis with respect to square root of area of wing blade.

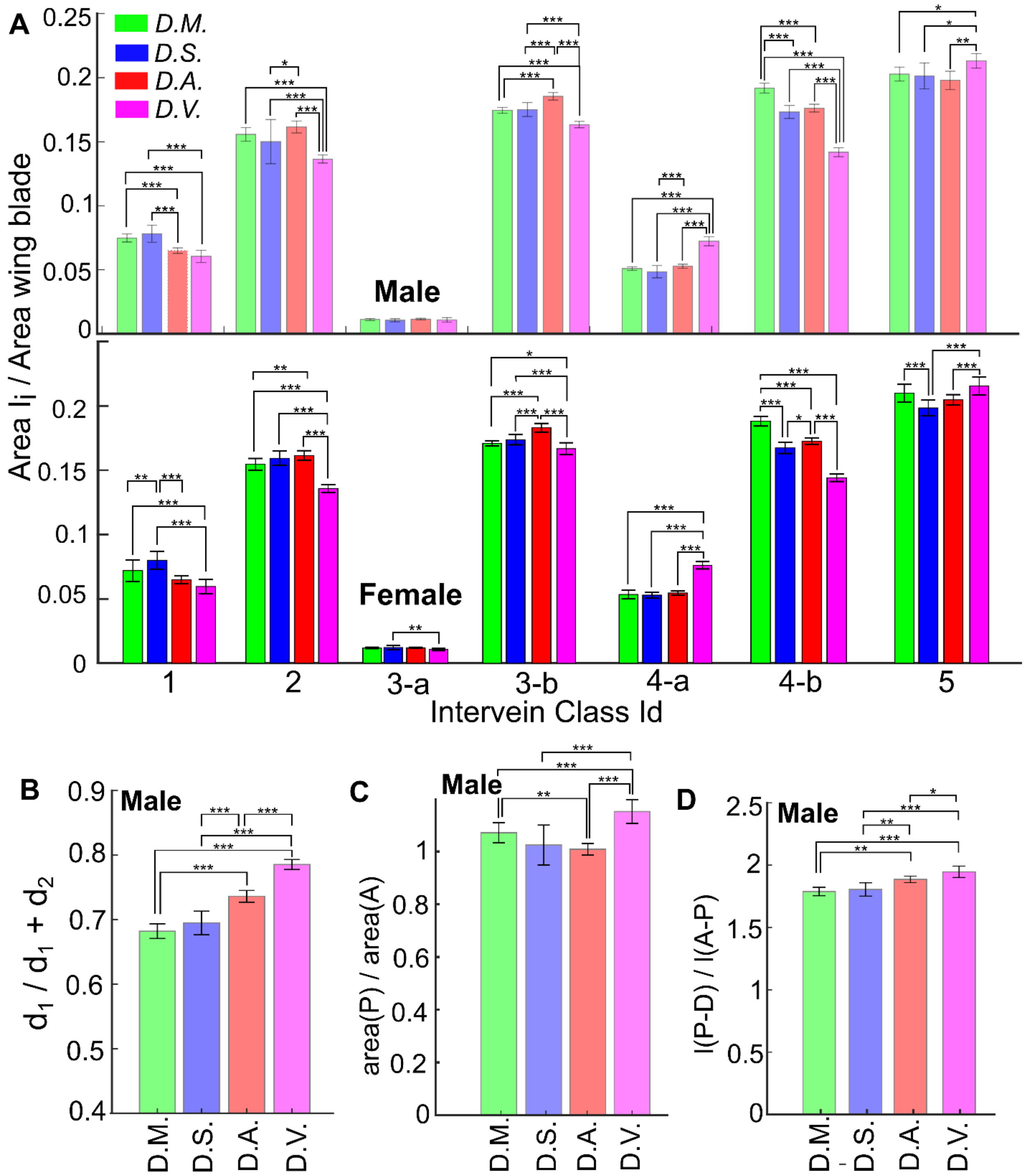

Figure S12. Differences in intervein areas and landmark positions in different *Drosophila* species identified by MAPPER. (A) Ratio of individual intervein areas normalized to the overall wing blade area. Each subgroup of the bar plot indicates the quantification of the ratio for four different species indicated in the legends. The analysis has been carried out for the seven distinct intervein regions, whose labels are indicated in the x-axis label at the bottom of the panel. Top and bottom rows indicate analysis done for the male and the female sexes of each individual species respectively. **(B)** Quantification of shift in posterior cross vein position in male wings (L_1 is defined as the segment of L_5 from the proximal to posterior cross

vein, L_2 is defined as the segment of L_5 from the posterior cross vein to the end of L_5). **(C)** Relative anterior to posterior areas in female wings for each species. **(D)** The ratio of the length of the proximal- distal axis and the anterior-posterior axis for females from different species. Error bars are representative of standard deviation. (*** p-value < 0.001, ** p-value < 0.01, * p-value < 0.05)

A Figure 7B

Group 1	Group 2	p-value
DAF	DAM	1.67E-06
DMF	DMM	1.37E-06
DSF	DSM	1.87E-06
DVF	DVM	0.03

B Figure 7C'

Group 1	Group 2	p-value
DAF	DMF	1.88E-08
DAF	DSF	1.50E-08
DAF	DVF	1.15E-08
DMF	DSF	0.59
DMF	DVF	2.26E-08
DSF	DVF	2.26E-08

C Figure 7D'

Group 1	Group 2	p-value
DAF	DMF	7.78E-04
DAF	DSF	0.07
DAF	DVF	2.26E-08
DMF	DSF	3.64E-08
DMF	DVF	4.26E-03
DSF	DVF	2.26E-08

D Figure 7G

Group 1	Group 2	p-value
DAF	DMF	3.77E-09
DAF	DSF	3.78E-09
DAF	DVF	0.48
DMF	DSF	0.99
DMF	DVF	3.76E-09
DSF	DVF	3.76E-09

Figure S13. Statistical testing for differences in wing features of different *Drosophila* species. Tables containing *p*-values from a Tukey honestly significant difference statistical test performed to check statistical significance between wing features of different *Drosophila* species. The heading of the panels indicate the main text figure panel the table is describing (Figure 7). Further details about the statistical tests can be found in section SI5.

S9 References

1. Falk T, Mai D, Bensch R, Çiçek Ö, Abdulkadir A, Marrakchi Y, et al. U-Net: deep learning for cell counting, detection, and morphometry. Nat Methods. 2019 Jan;16(1):67–70.

2. Ronneberger O, Fischer P, Brox T. U-Net: Convolutional Networks for Biomedical Image Segmentation. In: Navab N, Hornegger J, Wells WM, Frangi AF, editors. Medical Image Computing and Computer-Assisted Intervention – MICCAI 2015. Cham: Springer International Publishing; 2015. p. 234–41. (Lecture Notes in Computer Science).

3. Goodfellow I, Bengio Y, Courville A. Deep Learning [Internet]. MIT Press; 2016 [cited 2020 Dec 15]. Available from: https://www.deeplearningbook.org/

4. Kuhl FP, Giardina CR. Elliptic Fourier features of a closed contour. Comput Graph Image Process. 1982 Mar 1;18(3):236–58.

5. Sonnenschein A, VanderZee D, Pitchers WR, Chari S, Dworkin I. An image database of Drosophila melanogaster wings for phenomic and biometric analysis. GigaScience [Internet]. 2015 Dec 1 [cited 2020 Dec 1];4(1). Available from: https://academic.oup.com/gigascience/article/4/1/s13742-015-0065-6/2707551

6. Wold S, Esbensen K, Geladi P. Principal component analysis. Chemom Intell Lab Syst. 1987 Aug 1;2(1):37–52.

7. Yang M-S, Lai C-Y, Lin C-Y. A robust EM clustering algorithm for Gaussian mixture models. Pattern Recognit. 2012 Nov 1;45(11):3950–61.

8. Fisher RA. Statistical Methods for Research Workers. In: Kotz S, Johnson NL, editors. Breakthroughs in Statistics: Methodology and Distribution [Internet]. New York, NY: Springer; 1992 [cited 2020 Dec 4]. p. 66–70. (Springer Series in Statistics). Available from: https://doi.org/10.1007/978-1-4612-4380-9_6

9. Tukey JW. Comparing Individual Means in the Analysis of Variance. Biometrics. 1949;5(2):99–114.

10. Holm S. A Simple Sequentially Rejective Multiple Test Procedure. Scand J Stat. 1979;6(2):65–70.

11. Snedecor GW, Cochran WG. Statistical Methods, 8th Edition | Wiley [Internet]. eight edition. Iowa state University press; 1989 [cited 2021 Dec 16]. Available from: https://www.wiley.com/en-us/Statistical+Methods%2C+8th+Edition-p-9780813815619

12. Fisher RA. Applications of Student's distribution New tables for testing the significance of observations Expansion of student's integral in powers of n-1. Metron. 1925;5:90–104.

13. He C. densityplot(x,y,varargin) [Internet]. MATLAB Central File Exchange. [cited 2022 Jan 8]. Available from: https://www.mathworks.com/matlabcentral/fileexchange/65166-densityplot-x-y-varargin
