# Supplementary material for: MAPPER: An Open-Source, High-Dimensional Image Analysis Pipeline Unmasks Differential Regulation of Drosophila Wing Features
Source: Front Genet. 2022 Apr 11;13:869719. doi: 10.3389/fgene.2022.869719 (PMC9035675; doi:10.3389/fgene.2022.869719)

MAPPER: An open-source, high-dimensional image analysis pipeline unmasks differential regulation of *Drosophila* wing features

Supplementary File 2

Notebook Author: Francisco Huizar

Manuscript Authorship:
Nilay Kumar[1,+], Francisco J. Huizar[1,+], Keity J. Farfan-Pira[2], Pavel A. Brodskiy[1], Dharsan K. Soundarrajan[1], Marcos Nahmad[2], Jeremiah J. Zartman[1,*]

[1]Department of Chemical and BIomolecular Engineering, University of Notre Dame, Notre Dame, IN, 46556, USA
[2] Department of Physiology, Biophysics, and Neurosciences, Center for Research and Advanced Studies of the National Polytechnical Institute (Cinvestav), Mexico City, Mexico 07360

[+]These authors contributed equally to this work.

[*]Correspondence: jzartman@nd.edu (mailto:jzartman@nd.edu), (+1) 574-631-0455

R setup and necessary packages for this code

```
rm(list=ls()) # Clear workspace
par(mfrow=c(1,1)) # Setup plot parameters
par(ps = 12, font.lab = 1) # Plot parameters
set.seed(1) # set random seed generator for reproducibility
knitr::opts_chunk$set(dev = c("svg"),
                      dpi = 600,
                      cache = TRUE,
                      comment = NA,
                      message=FALSE,
                      warning=FALSE,
                      error=FALSE)
library(tidyverse)
library(MASS)
library(reshape2)
library(pracma)
library(rcompanion)
library(BBmisc)
library(car)
```

Function for 95% median CI

Below is a function that calculates the exact confidence interval for a median from the DescTools (https://cran.r-project.org/web/packages/DescTools/index.html) package for descriptive statistics. The confidence interval is a non-parametric interval for the median using order statistics to deal directly with error on the median. More information can be found here (http://www.stat.umn.edu/geyer/old03/5102/notes/rank.pdf) and here (https://hbiostat.org/doc/bbr.pdf). Code has been adapted from a StackExchange discussion here (https://stats.stackexchange.com/questions/186957/is-there-a-reliable-nonparametric-confidence-interval-for-the-mean-of-a-skewed-d).

```
cimed <- function(x, alpha=0.05, na.rm=FALSE) {
  if(na.rm) x <- x[! is.na(x)]
  n <- length(x)
  k <- qbinom(p=alpha / 2, size=n, prob=0.5, lower.tail=TRUE)
  ## Actual CL: 1 - 2 * pbinom(k - 1, size=n, prob=0.5) >= 1 - alpha
  sort(x)[c(k, n - k + 1)]
}
```

Function for 95% mean CI

Below is a function that calculates the confidence interval for a mean.

```
cimean <- function(x, alpha=0.05, na.rm=FALSE) {
  if(na.rm) x <- x[! is.na(x)]
  lowerBound = mean(x) - qnorm(1-alpha/2)*std_err(x)
  upperBound = mean(x) + qnorm(1-alpha/2)*std_err(x)
  return(c(lowerBound, upperBound))
}
```

Function to check if we can use the unpaired T-test

Create a function "canUseUnpairedTTest" to first check if data sets indicate distributions are not significantly different from normal using Shapiro-Wilk's. Explation (http://www.sthda.com/english/wiki/normality-test-in-r). Then check to see if data sets do not differ in their variances significantly. Explanation (http://www.sthda.com/english/wiki/f-test-compare-two-variances-in-r). It will perform an unpaired T-test if all normality and variability criteria are met, else perform Mann-Whitney U Test. Explanation (https://www.rdocumentation.org/packages/stats/versions/3.6.2/topics/wilcox.test)

```
canUseUnpairedTTest = function(df1, df2, alpha = 0.05){
  p1 = shapiro.test(df1)$p
  p2 = shapiro.test(df2)$p
  p3 = var.test(df1, df2, alternative = "two.sided")$p.value
  if (p1 > alpha & p2 > alpha & p3 > alpha) {
    print("Unpaired T-test is viable")
    t.test(df1, df2, var.equal = TRUE, conf.level = 1-alpha)
  } else {
    print("Unpaired T-test not viable")
    print(wilcox.test(df1, df2,
                      correct = FALSE,
                      alternative = "two.sided", paired = FALSE, exact=TRUE, conf.level = 1-alph
a))
  }
}
```

Function to add linear fit to plot

The function below adds a simple linear regression fit line to a plot with the coefficients and R^2 value.

```
lm_eqn <- function(x, y, df){
    m <- lm(y ~ x, df);
    eq <- substitute(italic(y) == a + b %.% italic(x)*","~~italic(r)^2~"="~r2,
         list(a = format(unname(coef(m)[1]), digits = 3),
              b = format(unname(coef(m)[2]), digits = 3),
             r2 = format(summary(m)$r.squared, digits = 4)))
    as.character(as.expression(eq));
}
```

Load the wing measurement data

Load in the data from the publicly available Samarkand species *Drosophila*. Source (https://doi.org/10.1186/s13742-015-0065-6)

```
trichomeValidation_df = read.csv("MAPPER_Trichome_Validation.csv", header=T, sep=",") %>% tibble
()
maleValidation_df = read.csv("MAPPER_Male_Validation.csv", header=T, sep=",") %>% tibble()
femaleValidation_df = read.csv("MAPPER_Female_Validation.csv", header=T, sep=",") %>% tibble()
```

Observe the data

Female trichome counts for automated and manual measurements

```
trichomeValidation_df
```

Filename<br><chr>	auto<br><int>	manual<br><int>
'samw_F_L_oly_4X_12.TIF'	38	39
'samw_F_L_oly_4X_13.TIF'	34	36
'samw_F_L_oly_4X_14.TIF'	45	47
'samw_F_L_oly_4X_16.TIF'	44	47
'samw_F_L_oly_4X_17.TIF'	46	49
'samw_F_L_oly_4X_18.TIF'	39	40
'samw_F_L_oly_4X_19.TIF'	44	47
'samw_F_L_oly_4X_20.TIF'	39	41
'samw_F_L_oly_4X_21.TIF'	40	40
'samw_F_L_oly_4X_22.TIF'	37	38

1-10 of 50 rows Previous **1** 2 3 4 5 Next

Male landmark region axes lengths for automated and manual measurements

```
maleValidation_df
```

fileName<br><chr>	Manual_Area<br><dbl>	Manual_...<br><dbl>	Manual_...<br><dbl>	Auto_Area<br><dbl>	Auto_PD<br><dbl>	Auto_AP<br><dbl>
samw_M_L_oly_4X_2	1.0294454	1.0070772	1.0200023	1.0222945	1.0169666	1.0185184
samw_M_L_oly_4X_3	1.0235861	0.9996394	1.0123714	1.0161583	1.0033147	1.0218755
samw_M_L_oly_4X_4	0.8345379	0.9118734	0.9055382	0.8308800	0.9117930	0.9176063
samw_M_L_oly_4X_6	0.9854569	0.9951767	0.9945658	0.9739237	0.9992731	0.9949929
samw_M_L_oly_4X_7	1.0327744	1.0041021	1.0149150	1.0247857	1.0186712	1.0113763
samw_M_L_oly_4X_9	1.0137764	0.9951767	1.0098277	1.0136164	1.0122733	1.0093106
samw_M_L_oly_4X_10	0.9977524	0.9847638	0.9945658	0.9986542	1.0076023	0.9997926
samw_M_L_oly_4X_12	1.0606501	1.0115398	1.0149150	1.0625281	1.0352939	1.0226275

fileName	Manual_Area	Manual_…	Manual_…	Auto_Area	Auto_PD	Auto_AP
<chr>	<dbl>	<dbl>	<dbl>	<dbl>	<dbl>	<dbl>
samw_M_L_oly_4X_13	0.9801303	0.9713758	0.9742167	0.9767741	0.9860877	0.9905826
samw_M_L_oly_4X_14	1.0089381	0.9907140	0.9996531	1.0055828	1.0055429	0.9906366

1-10 of 63 rows Previous 1 2 3 4 5 6 7 Next

Female intervein regions for automated and manual measurements

```
femaleValidation_df
```

Filename	ml1	ml2	ml3	ml4	ml5	ml6	ml7	
<chr>	<dbl>	<dbl>	<dbl>	<dbl>	<dbl>	<dbl>	<dbl>	
samw_F_L_oly_4X_12	0.203088	0.371724	0.027880	0.412724	0.125244	0.452380	0.521792	2.37
samw_F_L_oly_4X_13	0.200012	0.360328	0.025788	0.405160	0.122764	0.439664	0.523044	2.33
samw_F_L_oly_4X_14	0.186132	0.340744	0.026380	0.392228	0.111560	0.439944	0.492428	2.23
samw_F_L_oly_4X_16	0.184328	0.359520	0.025168	0.394276	0.115384	0.438752	0.503520	2.28
samw_F_L_oly_4X_17	0.178748	0.325172	0.025784	0.373728	0.108416	0.415796	0.465756	2.13
samw_F_L_oly_4X_18	0.192828	0.355896	0.029924	0.398212	0.119648	0.446324	0.515820	2.31
samw_F_L_oly_4X_19	0.206124	0.361736	0.025992	0.404040	0.120228	0.455676	0.549796	2.39
samw_F_L_oly_4X_20	0.206388	0.385132	0.030484	0.431304	0.127232	0.483060	0.554732	2.47
samw_F_L_oly_4X_21	0.179348	0.331792	0.026472	0.375560	0.116208	0.419692	0.485508	2.18
samw_F_L_oly_4X_22	0.194844	0.358464	0.028932	0.405612	0.120100	0.448308	0.506488	2.32

1-10 of 49 rows | 1-9 of 17 columns Previous 1 2 3 4 5 Next

Trichome validation plots

Violin Plot

Plotted is the distribution of the trichome counts of 50x50 pixel areas done manually and compared to MAPPER's automated output. Blue line indicates the median and the red lines correspond to the 95% CI of the median.

```
#svg("MAPPERValidationPlots/trichomeValidationViolin.svg", family = "arial", width=3, height=2)

ggplot(melt(trichomeValidation_df),
       aes(x=factor(variable),
           y=value)) +
  geom_violin(scale="width", alpha=0.50, fill="lightslateblue") +
  geom_jitter(width=0.25) +
  stat_summary(fun="median", geom="crossbar", size=0.5, fill="blue", color = "blue") +
  stat_summary(fun="cimed", geom="crossbar", size=0.5, fill="red", color = "red") +
  #stat_summary(fun="mean", geom="crossbar", size=0.5, fill="blue", color = "blue") +
  #stat_summary(fun="cimean", geom="crossbar", size=0.5, fill="red", color = "red") +
  theme(axis.text.x = element_text(angle = 45, vjust = 0.6),
        axis.title.x=element_blank()) +
  labs(x=expression(paste("")), y=expression(paste("50 x 50 pixel area trichome count"))) +
  theme(panel.grid.major = element_blank(), panel.grid.minor = element_blank(),
panel.background = element_blank(), axis.line = element_line(colour = "black")) +
  ylim(c(33,52))
```

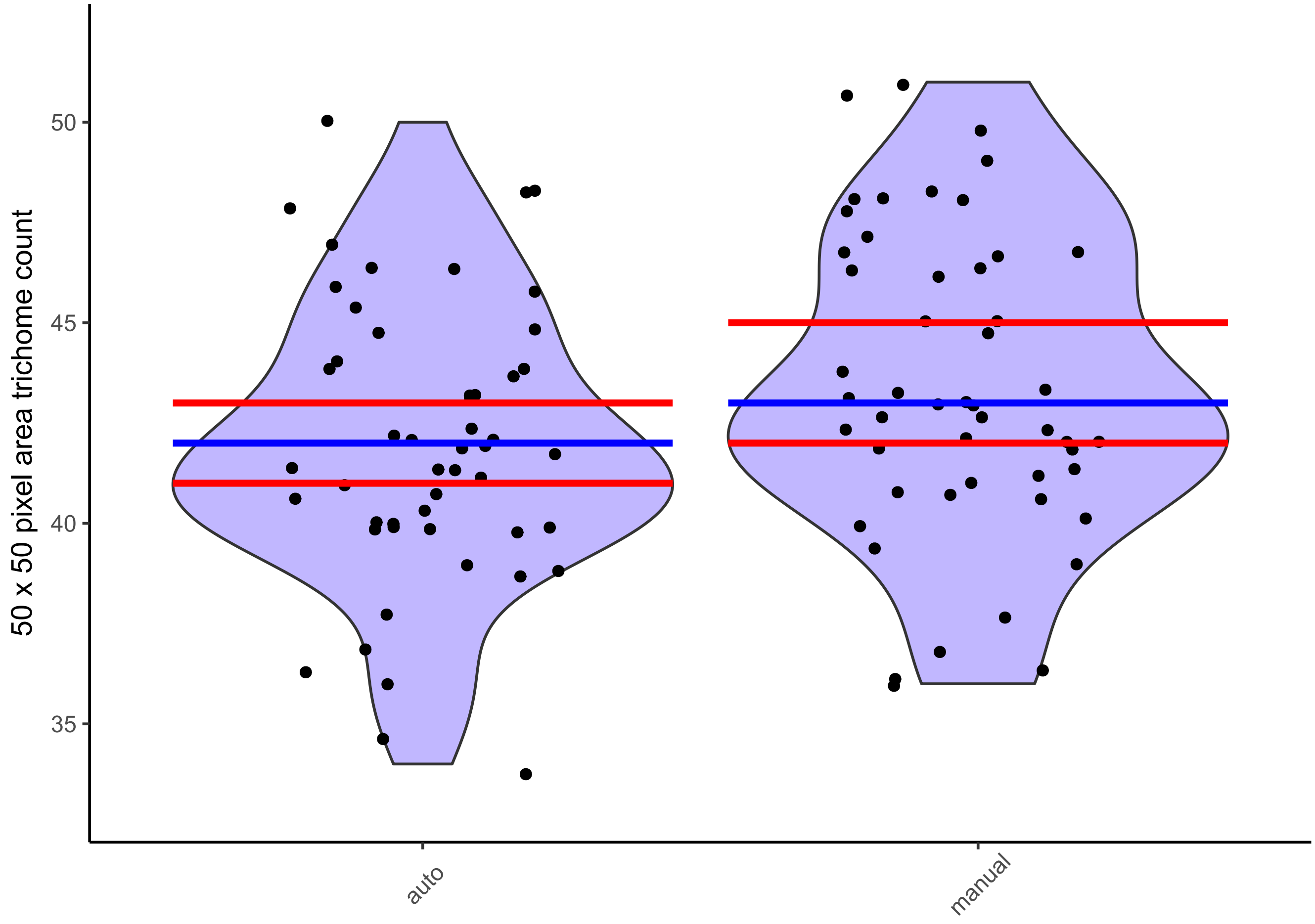

Generalized linear model

Because the dataset is discrete, we cannot fit a simple linear regression model and must use a generalized linear model.

Poisson regression model

Because we deal with count data (*i.e.*, number of trichomes in a 50x50 pixel area), we will fit a Poisson GLM. Below is a sumary of the fit of trichome counts as a function of a categorical variable that specifies whether or not the data came from MAPPER's automated output or manual hand measurements.

```
glmData = melt(trichomeValidation_df)
trichomeGLM = glm(glmData$value ~ as.factor(glmData$variable), family=poisson())
trichomeGLM %>% summary()
```

```


Call:
glm(formula = glmData$value ~ as.factor(glmData$variable), family = poisson())

Deviance Residuals:
     Min        1Q    Median        3Q       Max
-1.27412  -0.30804  -0.06384   0.40599   1.20124

Coefficients:
                                  Estimate Std. Error z value Pr(>|z|)
(Intercept)                        3.73719    0.02183 171.219   <2e-16 ***
as.factor(glmData$variable)manual  0.03373    0.03061   1.102    0.271
---
Signif. codes:  0 '***' 0.001 '**' 0.01 '*' 0.05 '.' 0.1 ' ' 1

(Dispersion parameter for poisson family taken to be 1)

    Null deviance: 32.192  on 99  degrees of freedom
Residual deviance: 30.978  on 98  degrees of freedom
AIC: 594.2

Number of Fisher Scoring iterations: 3
```

95% CIs of parameter estimates

Let's take the parameter estimates and generate 95% confidence intervals for the parameter fits.

```
cov.m1 <- vcov(trichomeGLM)
std.err <- sqrt(diag(cov.m1))
r.est <- cbind(Estimate= coef(trichomeGLM), "Standard Error" = std.err,
"Pr(>|z|)" = 2 * pnorm(abs(coef(trichomeGLM)/std.err), lower.tail=FALSE),
LL = coef(trichomeGLM) - 1.96 * std.err,
UL = coef(trichomeGLM) + 1.96 * std.err)
rownames(r.est) = c("b0", "b1")
r.est
```

```
     Estimate Standard Error  Pr(>|z|)          LL         UL
b0 3.73719331     0.02182692 0.0000000  3.69441254 3.77997409
b1 0.03372685     0.03061092 0.2705524 -0.02627055 0.09372425
```

Save and store variables to generate a plot.

```
Estimate= coef(trichomeGLM)
LL = coef(trichomeGLM) - 1.96 * std.err
UL = coef(trichomeGLM) + 1.96 * std.err

names(Estimate) = c("b0","b1")
names(LL) = c("b0", "b1")
names(UL) = c("b0", "b1")

print(c("Lower bound:", exp(LL)))
```

```
                                     b0                  b1
     "Lower bound:"  "40.2219370203658" "0.974071514640009"
```

```
print(c("Estimate:", exp(Estimate)))
```

```
                                   b0                 b1
       "Estimate:" "41.9800000006943" "1.03430204860508"
```

```
print(c("Upper bound:", exp(UL)))
```

```
                                   b0                 b1
    "Upper bound:" "43.8149062578952" "1.09825686478886"
```

Poisson Plot

The second fit parameter (associated with whether a categorical variable X_1 is "manual" or "automated") has a 95% confidence interval of [0.974, 1.098]. Because this parameter confidence interval contains a value of 1, we can infer that there is not statistical difference in trichome counts whether it is done manually or automated. This is further noted by $p = 0.271$ on the GLM output from above for the fit parameter. Below I take the confidence intervals and generate predicted values for the Poisson GLM given our original dataset. I then plot the upper and lower confidence intervals of the Poisson GLM predictions. Because the confidence bands overlap, it helps us visualize no statistical difference between trichome counts done manually vs. automated.

```
testX = as.factor(glmData$variable) %>% as.integer()-1
yPred = (exp(Estimate[1])+exp(Estimate[2])*testX)
yLL = (exp(LL[1])+exp(LL[2])*testX)
yUL = (exp(UL[1])+exp(UL[2])*testX)
```

```
#svg("MAPPERValidationPlots/trichomeValidationPoisson.svg", family = "arial", width=3, height=2)

poissondf = tibble(predicted = yPred, lowerB = yLL, upperB = yUL, variable = as.factor(glmData$v
ariable))
ggplot(poissondf,
       aes(x=variable,
           y=predicted)) +
  geom_errorbar(aes(ymin = yLL, ymax = yUL), fill="lightslateblue") +
  theme(axis.text.x = element_text(angle = 45, vjust = 0.6),
        axis.title.x=element_blank()) +
  labs(x=expression(paste("")), y=expression(paste("50 x 50 pixel area trichome count"))) +
  theme(panel.grid.major = element_blank(), panel.grid.minor = element_blank(),
panel.background = element_blank(), axis.line = element_line(colour = "black")) +
  ylim(c(33,52))
```

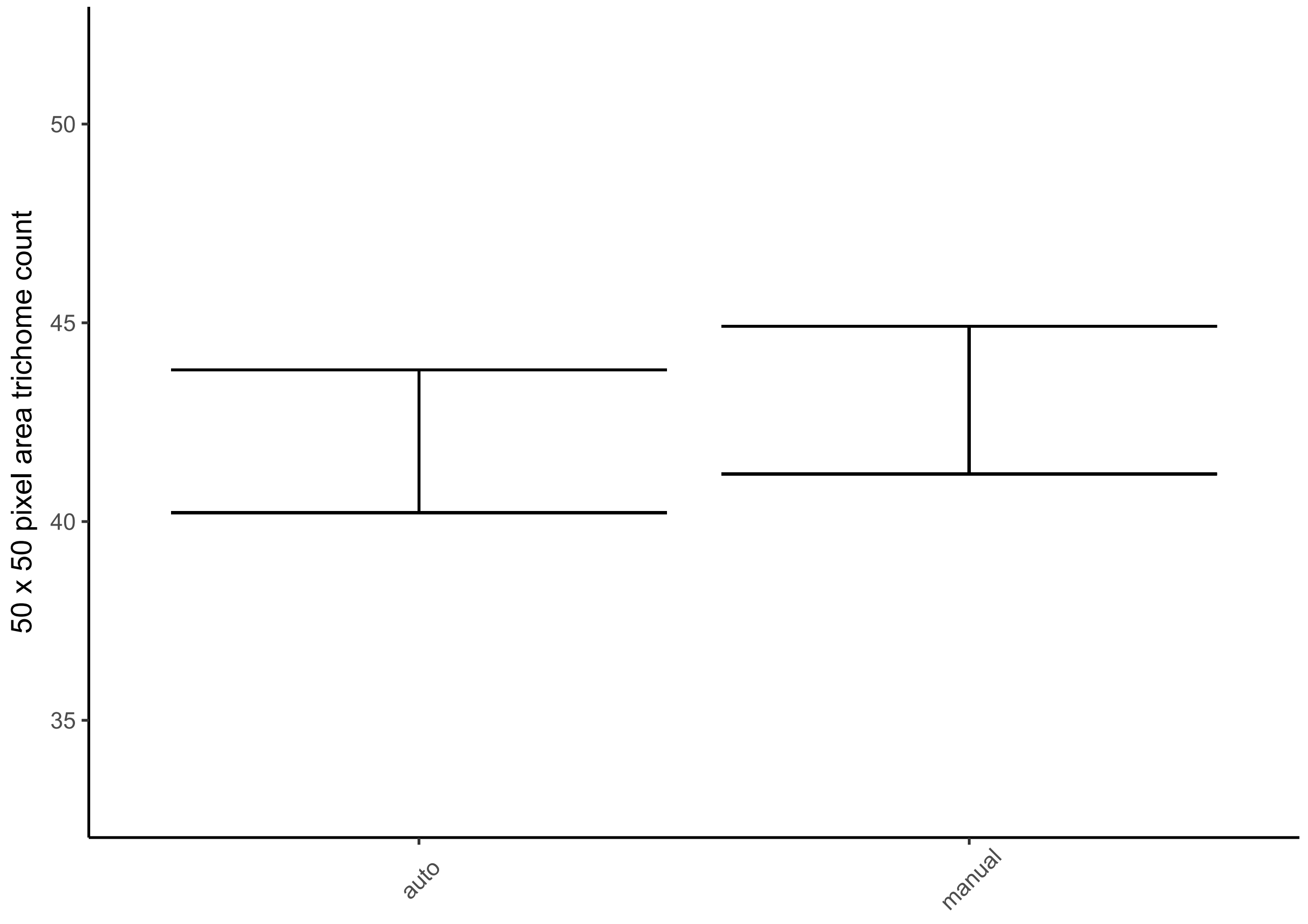

Male dataset validation plots

Check if we should use the unpaired T-test of Mann-Whitney U-Test for statistical comparisons. For all male comparisons of MAPPER automated vs. manual measurements, there is no statistical differences. All violin plot distributions are comparable (with medians plotted as a blue line and 95% CIs plotted as red lines). Medians are used instead of means because the comparisons made do not pass the normality and variance assumptions for unpaired T-tests to be used.

Total wing areas (Mann-Whitney U)

Male total wing areas MAPPER vs. manual are not statistically different.

```
canUseUnpairedTTest(maleValidation_df$Manual_Area, maleValidation_df$Auto_Area)
```

```
[1] "Unpaired T-test not viable"

    Wilcoxon rank sum test

data:  df1 and df2
W = 1984, p-value = 0.9981
alternative hypothesis: true location shift is not equal to 0
```

PD Axis length (Mann-Whitney U)

Male PD Axis measurements for MAPPER vs. manual are not statistically different.

```
canUseUnpairedTTest(maleValidation_df$Manual_PD, maleValidation_df$Auto_PD)
```

```
[1] "Unpaired T-test not viable"

    Wilcoxon rank sum test

data:  df1 and df2
W = 1933, p-value = 0.8016
alternative hypothesis: true location shift is not equal to 0
```

AP Axis length (Mann-Whitney U)

Male AP Axis measurements for MAPPER vs. manual are not statistically different.

```
canUseUnpairedTTest(maleValidation_df$Manual_AP, maleValidation_df$Auto_AP)
```

```
[1] "Unpaired T-test not viable"

    Wilcoxon rank sum test

data:  df1 and df2
W = 1922, p-value = 0.7604
alternative hypothesis: true location shift is not equal to 0
```

Generate Actual Plots

Total Area

Plotted is the distribution of the total wing areas done manually and compared to MAPPER's automated output. Blue line indicates the median with the red lines being the 95% CI of the median.

```
#svg("MAPPERValidationPlots/maleTotalArea.svg", family = "arial", width=3, height=2)

ggplot(melt(maleValidation_df) %>% dplyr::filter(variable=="Auto_Area" | variable =="Manual_Are
a"),
       aes(x=factor(variable),
           y=value)) +
  geom_violin(scale="width", alpha=0.50, fill="lightslateblue") +
  geom_jitter(width=0.25) +
  stat_summary(fun="median", geom="crossbar", size=0.5, fill="blue", color = "blue") +
  stat_summary(fun="cimed", geom="crossbar", size=0.5, fill="red", color = "red") +
  #stat_summary(fun="mean", geom="crossbar", size=0.5, fill="blue", color = "blue") +
  #stat_summary(fun="cimean", geom="crossbar", size=0.5, fill="red", color = "red") +
  theme(axis.text.x = element_text(angle = 45, vjust = 0.6),
        axis.title.y=element_blank()) +
  labs(x=expression(paste(""))) +
  theme(panel.grid.major = element_blank(), panel.grid.minor = element_blank(),
panel.background = element_blank(), axis.line = element_line(colour = "black"))
```

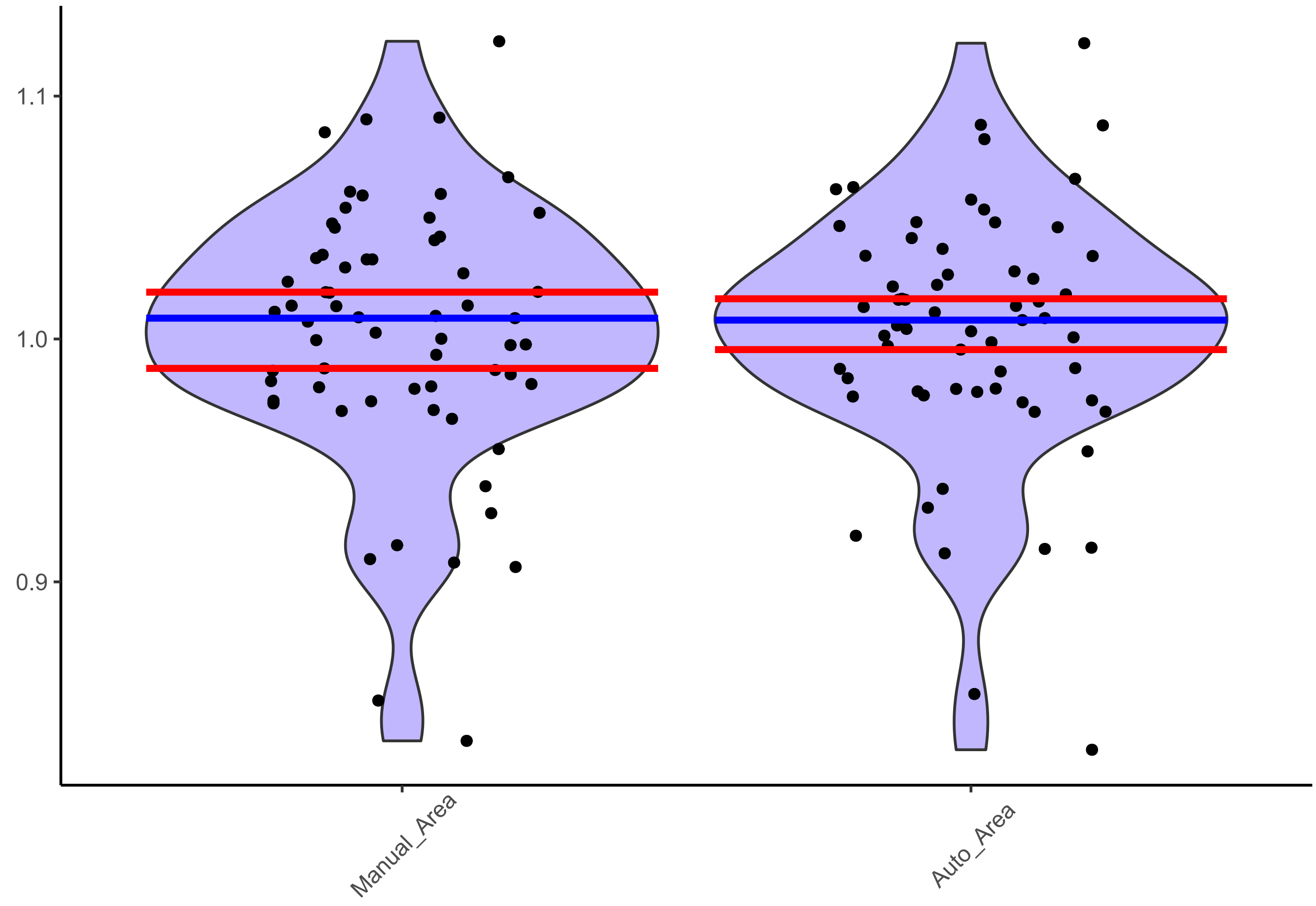

Plotted below is a simple linear fit for MAPPER vs. automated measurements. There is strong linear correlation between the two with nearly perfect fit.

```
#svg("MAPPERValidationPlots/maleTotalAreaLM.svg", family = "arial", width=3, height=2)

lmx = maleValidation_df$Auto_Area
lmy = maleValidation_df$Manual_Area
model_lm = lm(lmy~lmx)

ggplot(maleValidation_df,
       aes(x=Auto_Area,
           y=Manual_Area)) +
  geom_point() +
  geom_smooth(method=lm, color="red", fill="lightslateblue", se=TRUE) +
  geom_text(x = 0.9, y = 1.1, label = lm_eqn(maleValidation_df$Auto_Area, maleValidation_df$Manu
al_Area, maleValidation_df), parse = TRUE) +
  labs(x=expression(paste("MAPPER Area")), y=expression(paste("Manual Area"))) +
  theme(panel.grid.major = element_blank(), panel.grid.minor = element_blank(),
panel.background = element_blank(), axis.line = element_line(colour = "black"))
```

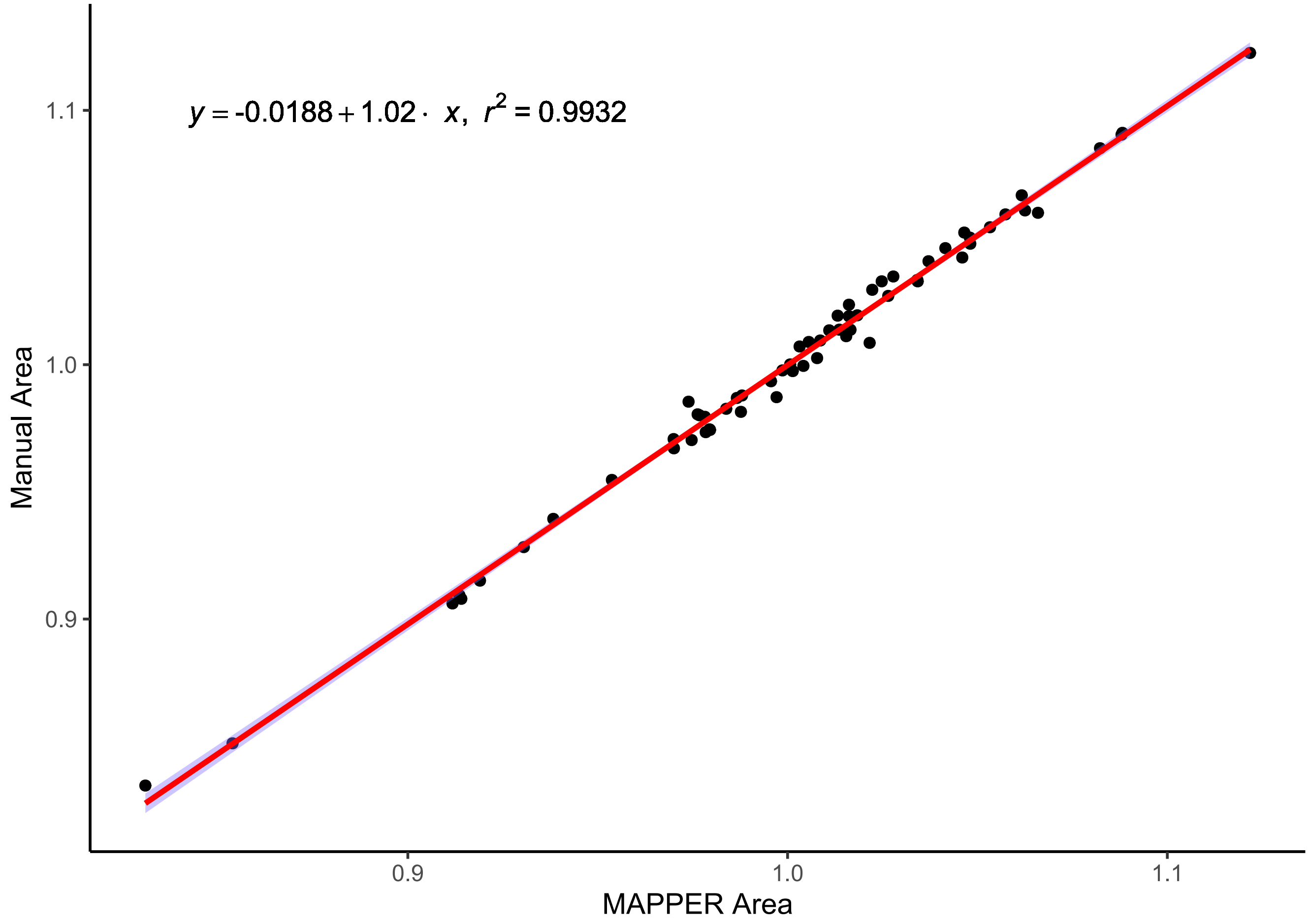

Below is the model summary for the linear fit.

```
lm(maleValidation_df$Manual_Area ~ maleValidation_df$Auto_Area) %>% summary()
```

```
Call:
lm(formula = maleValidation_df$Manual_Area ~ maleValidation_df$Auto_Area)

Residuals:
       Min         1Q     Median         3Q        Max 
-0.0132716 -0.0029308  0.0001853  0.0026206  0.0121771 

Coefficients:
                            Estimate Std. Error t value Pr(>|t|)    
(Intercept)                 -0.01879    0.01083  -1.736   0.0876 .  
maleValidation_df$Auto_Area  1.01864    0.01078  94.455   <2e-16 ***
---
Signif. codes:  0 '***' 0.001 '**' 0.01 '*' 0.05 '.' 0.1 ' ' 1

Residual standard error: 0.004461 on 61 degrees of freedom
Multiple R-squared:  0.9932,    Adjusted R-squared:  0.9931 
F-statistic:  8922 on 1 and 61 DF,  p-value: < 2.2e-16
```

Below are the slope parameter bounds.

```
parameter_value = model_lm$coefficients[2]
model_sum = model_lm %>% summary()
parameter_SE = model_sum$coefficients[ , 2][2]
LB_param = parameter_value - qnorm(0.975)*parameter_SE
UB_param = parameter_value + qnorm(0.975)*parameter_SE
cbind(LB_param, UB_param)
```

```
     LB_param UB_param
lmx 0.9974994 1.039773
```

Below is the significance test of the slope parameter for the null hypothesis $H_0 = 1.0$.

```
2*pt((-abs((parameter_value-1.0)/parameter_SE)),model_lm$df.residual)
```

```
       lmx 
0.08903059 
```

PD axis length

Plotted is the distribution of the PD axis length (scaled to the mean PD axis length of the group) done manually and compared to MAPPER's automated output. Blue line indicates the median with the red lines being the 95% CI of the median.

```
#svg("MAPPERValidationPlots/malePDAxisViolin.svg", family = "arial", width=3, height=2)

ggplot(melt(maleValidation_df) %>% dplyr::filter(variable=="Auto_PD" | variable =="Manual_PD"),
       aes(x=factor(variable),
           y=value)) +
  geom_violin(scale="width", alpha=0.50, fill="lightslateblue") +
  geom_jitter(width=0.25) +
  stat_summary(fun="median", geom="crossbar", size=0.5, fill="blue", color = "blue") +
  stat_summary(fun="cimed", geom="crossbar", size=0.5, fill="red", color = "red") +
  #stat_summary(fun="mean", geom="crossbar", size=0.5, fill="blue", color = "blue") +
  #stat_summary(fun="cimean", geom="crossbar", size=0.5, fill="red", color = "red") +
  theme(axis.text.x = element_text(angle = 45, vjust = 0.6),
        axis.title.y=element_blank()) +
  labs(x=expression(paste(""))) +
  theme(panel.grid.major = element_blank(), panel.grid.minor = element_blank(),
panel.background = element_blank(), axis.line = element_line(colour = "black"))
```

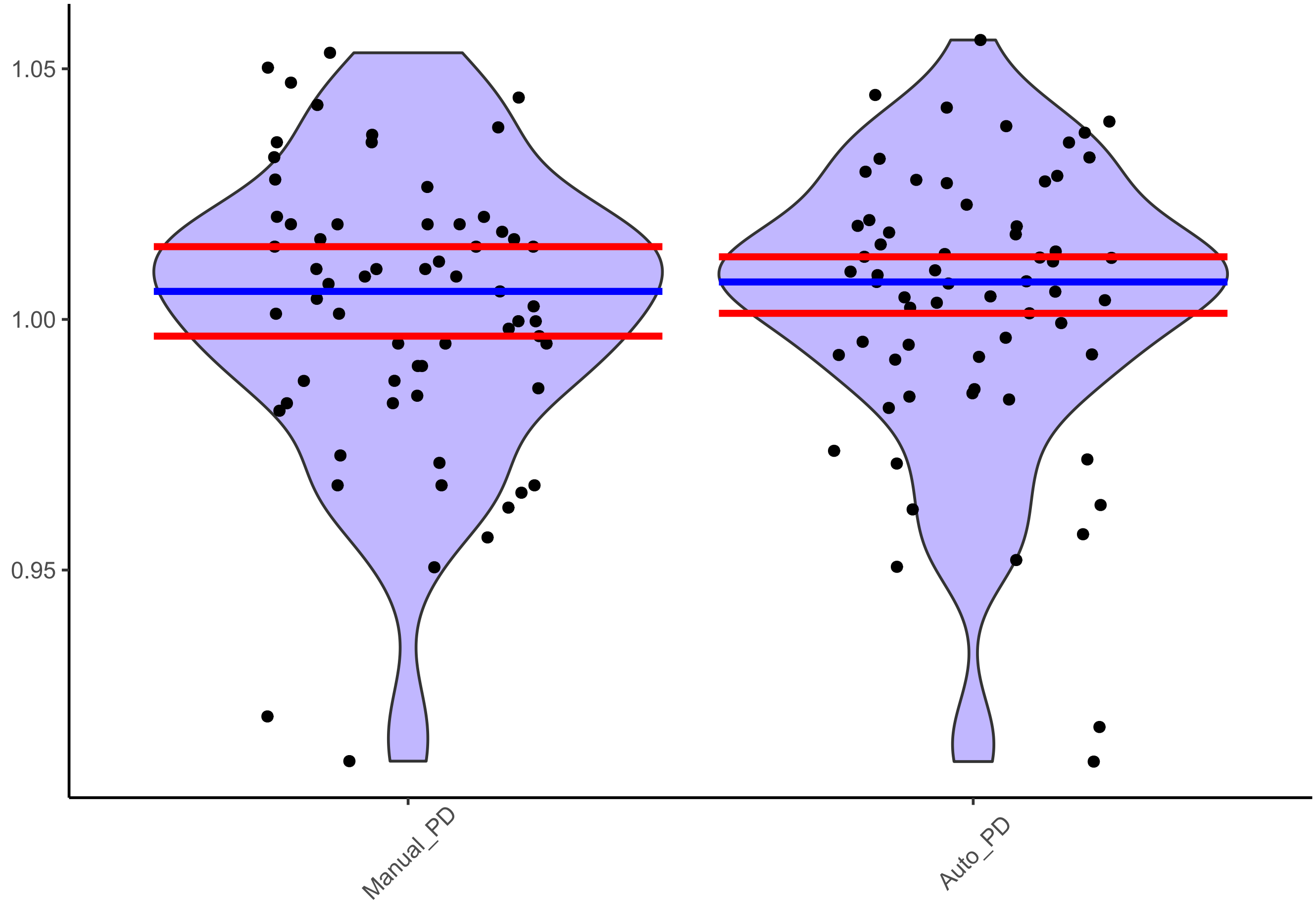

Plotted below is a simple linear fit for MAPPER vs. automated measurements. There is some linear correlation between the two.

```
#svg("MAPPERValidationPlots/malePDAxisLM.svg", family = "arial", width=3, height=2)

lmx = maleValidation_df$Auto_PD
lmy = maleValidation_df$Manual_PD
model_lm = lm(lmy~lmx)

ggplot(maleValidation_df,
       aes(x=Auto_PD,
           y=Manual_PD)) +
  geom_point() +
  geom_smooth(method=lm, color="red", fill="lightslateblue", se=TRUE) +
  geom_text(x = 0.96, y = 1.05, label = lm_eqn(maleValidation_df$Auto_PD, maleValidation_df$Manu
al_PD, maleValidation_df), parse = TRUE) +
  labs(x=expression(paste("MAPPER PD Length")), y=expression(paste("Manual PD Length"))) +
  theme(panel.grid.major = element_blank(), panel.grid.minor = element_blank(),
panel.background = element_blank(), axis.line = element_line(colour = "black"))
```

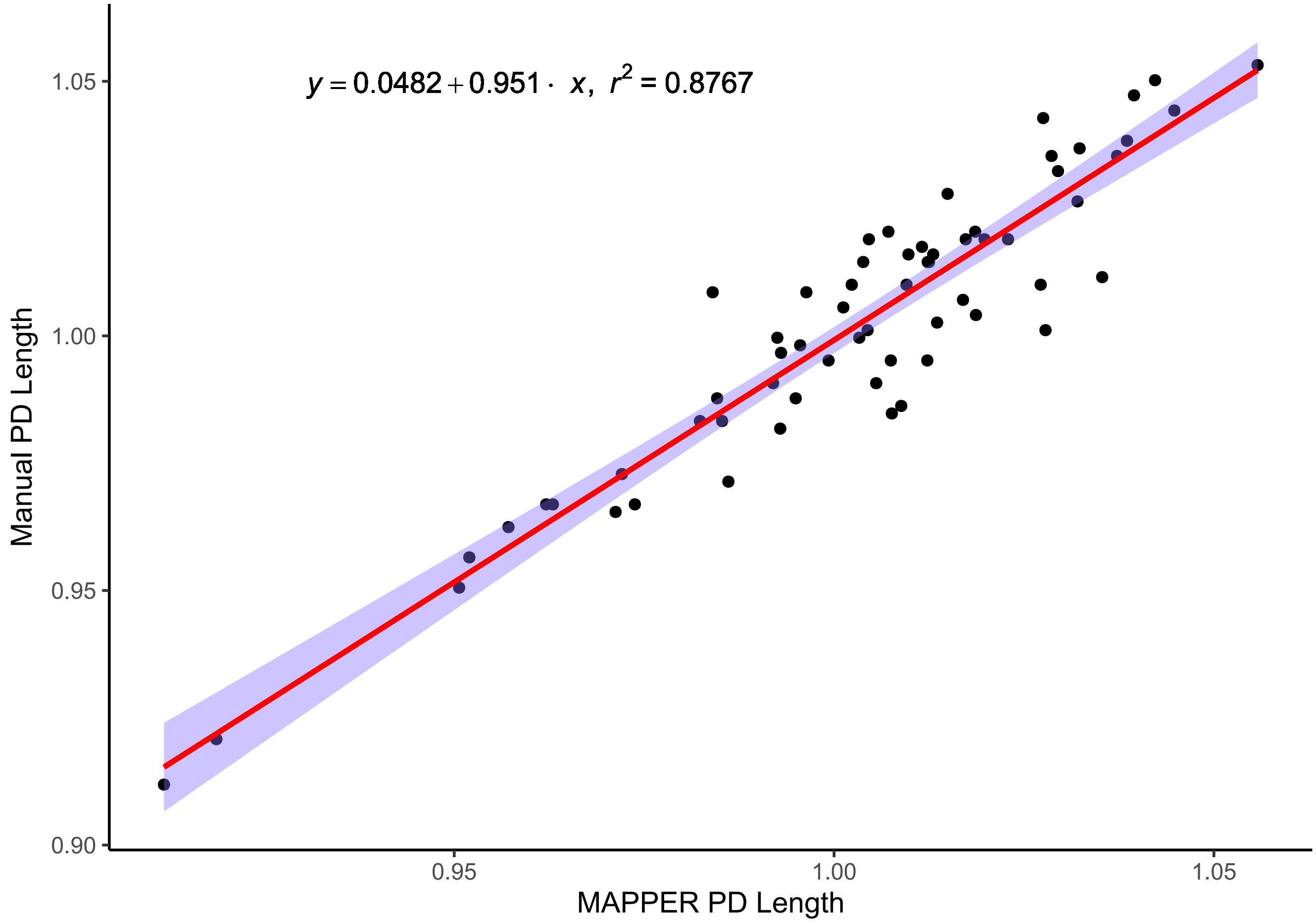

Below is the model summary for the linear fit.

```
lm(maleValidation_df$Manual_PD ~ maleValidation_df$Auto_PD) %>% summary()
```

```

Call:
lm(formula = maleValidation_df$Manual_PD ~ maleValidation_df$Auto_PD)

Residuals:
      Min        1Q    Median        3Q       Max 
-0.024523 -0.004917  0.001798  0.005201  0.024580 

Coefficients:
                          Estimate Std. Error t value Pr(>|t|)    
(Intercept)                0.04819    0.04581   1.052    0.297    
maleValidation_df$Auto_PD  0.95099    0.04566  20.828   <2e-16 ***
---
Signif. codes:  0 '***' 0.001 '**' 0.01 '*' 0.05 '.' 0.1 ' ' 1

Residual standard error: 0.01016 on 61 degrees of freedom
Multiple R-squared:  0.8767,    Adjusted R-squared:  0.8747 
F-statistic: 433.8 on 1 and 61 DF,  p-value: < 2.2e-16
```

Below are the slope parameter bounds.

```
parameter_value = model_lm$coefficients[2]
model_sum = model_lm %>% summary()
parameter_SE = model_sum$coefficients[ , 2][2]
LB_param = parameter_value - qnorm(0.975)*parameter_SE
UB_param = parameter_value + qnorm(0.975)*parameter_SE
cbind(LB_param, UB_param)
```

```
     LB_param UB_param
lmx 0.8614996 1.040481
```

Below is the significance test of the slope parameter for the null hypothesis $H_0 = 1.0$.

```
2*pt((-abs((parameter_value-1.0)/parameter_SE)),model_lm$df.residual)
```

```
      lmx 
0.2873314 
```

AP axis length

Plotted is the distribution of the AP axis length done manually and compared to MAPPER's automated output. Blue line indicates the median with the red lines being the 95% CI of the median.

```
#svg("MAPPERValidationPlots/maleAPAxisViolin.svg", family = "arial", width=3, height=2)

ggplot(melt(maleValidation_df) %>% dplyr::filter(variable=="Auto_AP" | variable =="Manual_AP"),
       aes(x=factor(variable),
           y=value)) +
  geom_violin(scale="width", alpha=0.50, fill="lightslateblue") +
  geom_jitter(width=0.25) +
  stat_summary(fun="median", geom="crossbar", size=0.5, fill="blue", color = "blue") +
  stat_summary(fun="cimed", geom="crossbar", size=0.5, fill="red", color = "red") +
  #stat_summary(fun="mean", geom="crossbar", size=0.5, fill="blue", color = "blue") +
  #stat_summary(fun="cimean", geom="crossbar", size=0.5, fill="red", color = "red") +
  theme(axis.text.x = element_text(angle = 45, vjust = 0.6),
        axis.title.y=element_blank()) +
  labs(x=expression(paste(""))) +
  theme(panel.grid.major = element_blank(), panel.grid.minor = element_blank(),
panel.background = element_blank(), axis.line = element_line(colour = "black"))
```

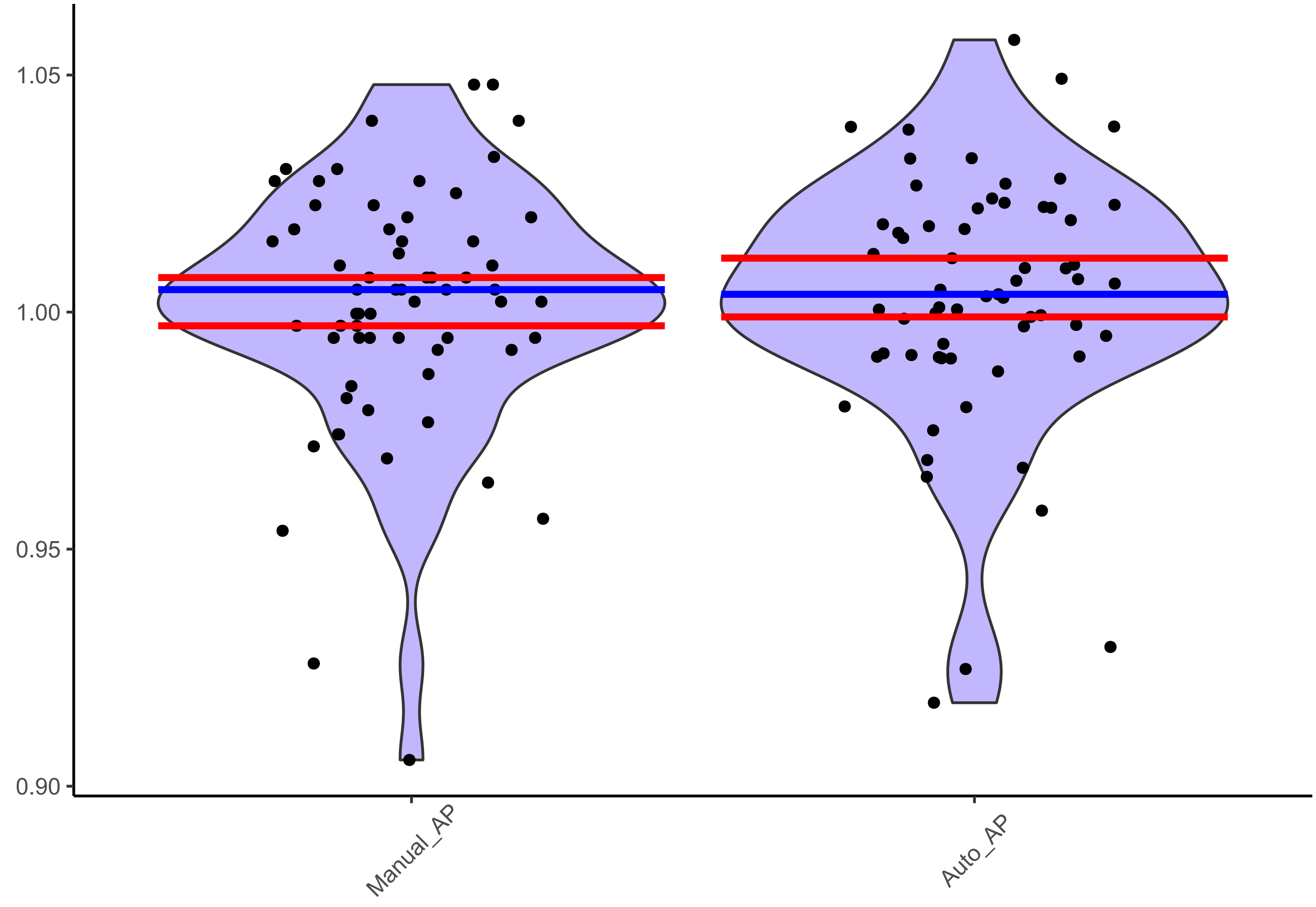

Plotted below is a simple linear fit for MAPPER vs. automated measurements. There is some linear correlation between the two.

```
#svg("MAPPERValidationPlots/maleAPAxisLM.svg", family = "arial", width=3, height=2)

lmx = maleValidation_df$Auto_AP
lmy = maleValidation_df$Manual_AP
model_lm = lm(lmy~lmx)

ggplot(maleValidation_df,
       aes(x=Auto_AP,
           y=Manual_AP)) +
  geom_point() +
  geom_smooth(method=lm, color="red", fill="lightslateblue", se=TRUE) +
  geom_text(x = 0.96, y = 1.05, label = lm_eqn(maleValidation_df$Auto_AP, maleValidation_df$Manu
al_AP, maleValidation_df), parse = TRUE) +
  labs(x=expression(paste("MAPPER AP Length")), y=expression(paste("Manual AP Length"))) +
  theme(panel.grid.major = element_blank(), panel.grid.minor = element_blank(),
panel.background = element_blank(), axis.line = element_line(colour = "black"))
```

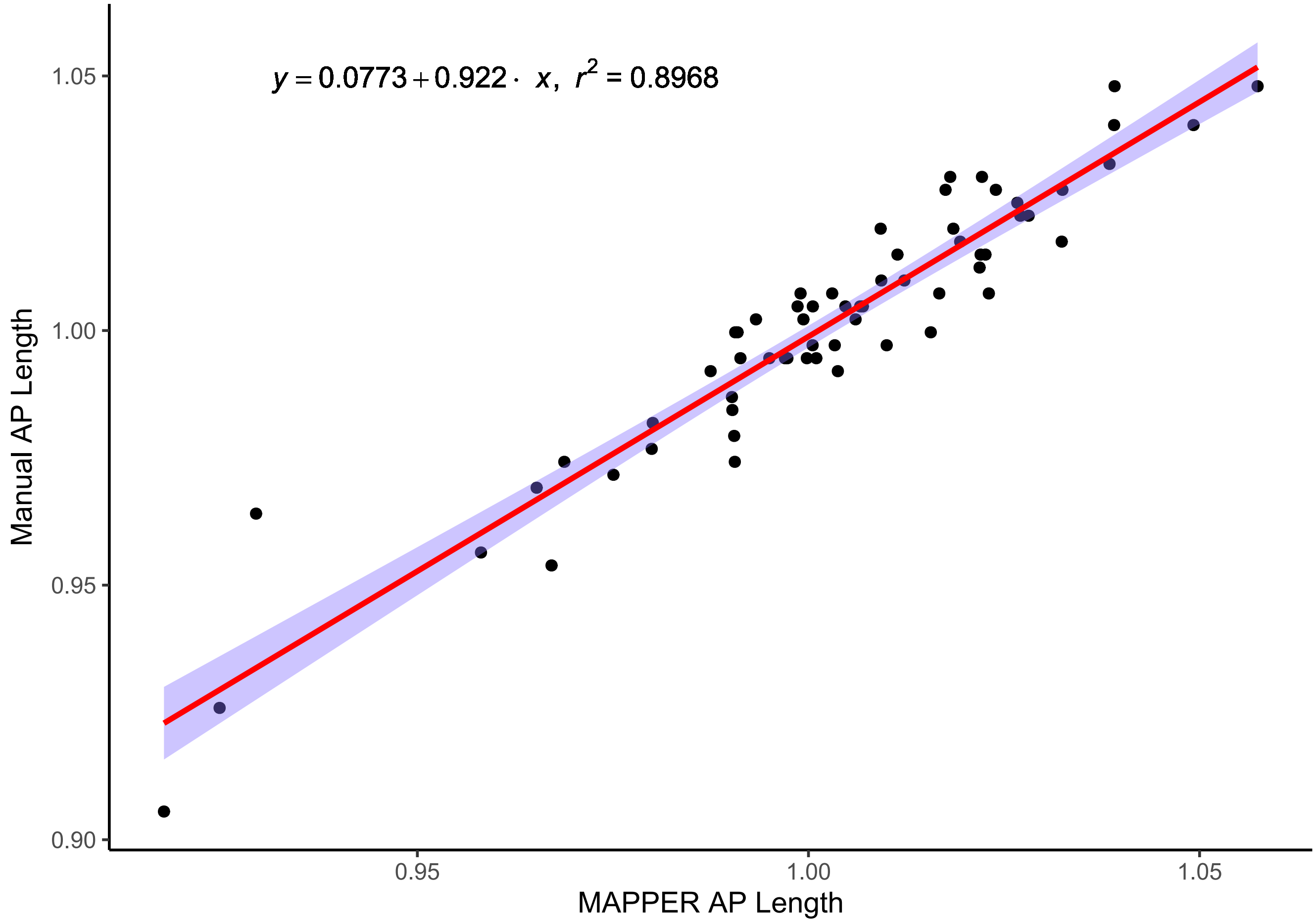

Below is the model summary for the linear fit.

```
lm(maleValidation_df$Manual_AP ~ maleValidation_df$Auto_AP) %>% summary()
```

```

Call:
lm(formula = maleValidation_df$Manual_AP ~ maleValidation_df$Auto_AP)

Residuals:
      Min        1Q    Median        3Q       Max 
-0.017346 -0.004170 -0.001094  0.005078  0.030309 

Coefficients:
                          Estimate Std. Error t value Pr(>|t|)    
(Intercept)                0.07727    0.04015   1.924    0.059 .  
maleValidation_df$Auto_AP  0.92155    0.04003  23.022   <2e-16 ***
---
Signif. codes:  0 '***' 0.001 '**' 0.01 '*' 0.05 '.' 0.1 ' ' 1

Residual standard error: 0.008452 on 61 degrees of freedom
Multiple R-squared:  0.8968,    Adjusted R-squared:  0.8951 
F-statistic:   530 on 1 and 61 DF,  p-value: < 2.2e-16
```

Below are the bounds for the slope parameter.

```
parameter_value = model_lm$coefficients[2]
model_sum = model_lm %>% summary()
parameter_SE = model_sum$coefficients[ , 2][2]
LB_param = parameter_value - qnorm(0.975)*parameter_SE
UB_param = parameter_value + qnorm(0.975)*parameter_SE
cbind(LB_param, UB_param)
```

```
    LB_param UB_param
lmx  0.84309 1.000001
```

Below is the significance test of the slope parameter for the null hypothesis $H_0 = 1.0$.

```
2*pt((-abs((parameter_value-1.0)/parameter_SE)),model_lm$df.residual)
```

```
       lmx 
0.05457695 
```

Combined violin plots

```
#svg("MAPPERValidationPlots/LengthAxisViolin.svg", family = "arial", width=3, height=2)

ggplot(melt(maleValidation_df) %>% dplyr::filter(variable=="Auto_PD" | variable =="Manual_PD" |
 variable =="Auto_AP" | variable =="Manual_AP"),
       aes(x=factor(variable),
           y=value)) +
  geom_violin(scale="width", alpha=0.50, fill="lightslateblue") +
  geom_jitter(width=0.25) +
  stat_summary(fun="median", geom="crossbar", size=0.5, fill="blue", color = "blue") +
  stat_summary(fun="cimed", geom="crossbar", size=0.5, fill="red", color = "red") +
  #stat_summary(fun="mean", geom="crossbar", size=0.5, fill="blue", color = "blue") +
  #stat_summary(fun="cimean", geom="crossbar", size=0.5, fill="red", color = "red") +
  theme(axis.text.x = element_text(angle = 45, vjust = 0.6),
        axis.title.y=element_blank()) +
  labs(x=expression(paste(""))) +
  theme(panel.grid.major = element_blank(), panel.grid.minor = element_blank(),
panel.background = element_blank(), axis.line = element_line(colour = "black"))
```

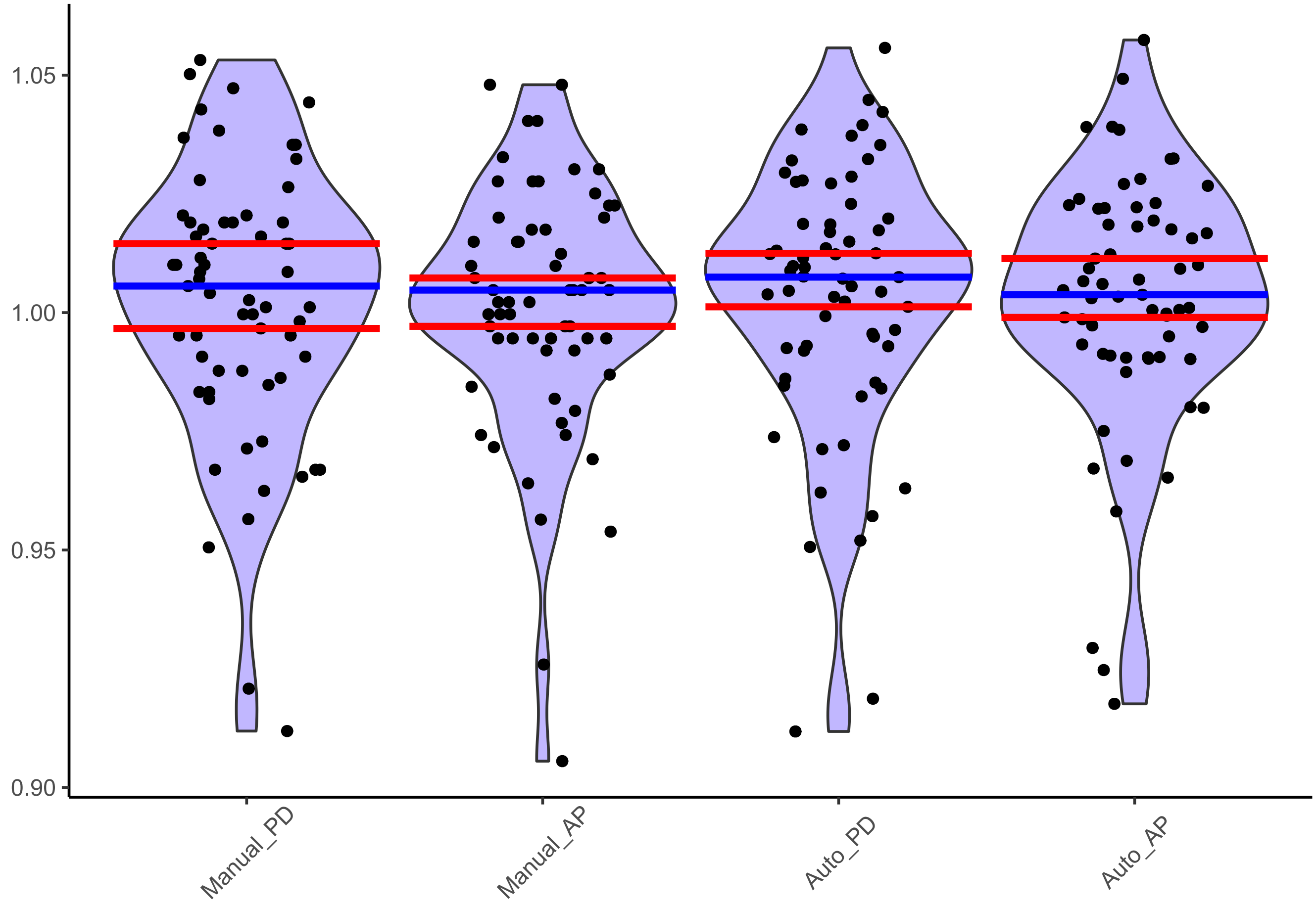

Female dataset validation plots

Check validity of unpaired T-test

Similar to before, check if we should use the unpaired T-test of Mann-Whitney U-Test for statistical comparisons. For all female comparisons of MAPPER automated vs. manual measurements, there is no statistical differences. All violin plot distributions are comparable (with means plotted as a blue line and 95% CIs plotted as red lines). Means are used instead of medians (seen previously in the male plots) because the comparisons made all pass the normality and variance assumptions for unpaired T-tests to be used. Categories plotted with the letter "m" prefix indicate "manual" measurements, and categories plotted with the letter "a" prefix indicate automated measurements. All simple linear fits have strong linear correlation. This indicates for the female data set, MAPPER performed consistently well.

IVA1 (T-test)

```
canUseUnpairedTTest(femaleValidation_df$mI1, femaleValidation_df$aI1)
```

```
[1] "Unpaired T-test is viable"
```

```

    Two Sample t-test

data:  df1 and df2
t = -0.19535, df = 96, p-value = 0.8455
alternative hypothesis: true difference in means is not equal to 0
95 percent confidence interval:
 -0.006270197  0.005146605
sample estimates:
mean of x mean of y
0.1962624 0.1968242
```

IVA2 (T-test)

```
canUseUnpairedTTest(femaleValidation_df$mI2, femaleValidation_df$aI2)
```

```
[1] "Unpaired T-test is viable"
```

```

    Two Sample t-test

data:  df1 and df2
t = -1.1469, df = 96, p-value = 0.2543
alternative hypothesis: true difference in means is not equal to 0
95 percent confidence interval:
 -0.012376536  0.003311883
sample estimates:
mean of x mean of y
0.3528869 0.3574193
```

IVA3 (T-test)

```
canUseUnpairedTTest(femaleValidation_df$mI3, femaleValidation_df$aI3)
```

```
[1] "Unpaired T-test is viable"
```

```


    Two Sample t-test

data:  df1 and df2
t = -1.3384, df = 96, p-value = 0.1839
alternative hypothesis: true difference in means is not equal to 0
95 percent confidence interval:
 -0.0017420328  0.0003389308
sample estimates:
 mean of x  mean of y 
0.02575649 0.02645804 
```

IVA4 (T-test)

```
canUseUnpairedTTest(femaleValidation_df$mI4, femaleValidation_df$aI4)
```

```
[1] "Unpaired T-test is viable"
```

```


    Two Sample t-test

data:  df1 and df2
t = -0.39276, df = 96, p-value = 0.6954
alternative hypothesis: true difference in means is not equal to 0
95 percent confidence interval:
 -0.01018941  0.00682321
sample estimates:
mean of x mean of y 
0.3992832 0.4009663 
```

IVA5 (T-test)

```
canUseUnpairedTTest(femaleValidation_df$mI5, femaleValidation_df$aI5)
```

```
[1] "Unpaired T-test is viable"
```

```
    Two Sample t-test

data:  df1 and df2
t = -0.95582, df = 96, p-value = 0.3416
alternative hypothesis: true difference in means is not equal to 0
95 percent confidence interval:
 -0.005587617  0.001955453
sample estimates:
mean of x mean of y 
0.1155311 0.1173472 
```

IVA6 (T-test)

```
canUseUnpairedTTest(femaleValidation_df$mI6, femaleValidation_df$aI6)
```

```
[1] "Unpaired T-test is viable"
```

```
    Two Sample t-test

data:  df1 and df2
t = -0.58367, df = 96, p-value = 0.5608
alternative hypothesis: true difference in means is not equal to 0
95 percent confidence interval:
 -0.012845149  0.007007598
sample estimates:
mean of x mean of y 
0.4421708 0.4450896 
```

IVA7 (T-test)

```
canUseUnpairedTTest(femaleValidation_df$mI7, femaleValidation_df$aI7)
```

```
[1] "Unpaired T-test is viable"
```

```
	Two Sample t-test

data:  df1 and df2
t = 0.51666, df = 96, p-value = 0.6066
alternative hypothesis: true difference in means is not equal to 0
95 percent confidence interval:
 -0.01107333  0.01886614
sample estimates:
mean of x mean of y 
0.5095768 0.5056804 
```

Total Area (T-test)

```
canUseUnpairedTTest(femaleValidation_df$mTA, femaleValidation_df$aTA)
```

```
[1] "Unpaired T-test is viable"
```

```
	Two Sample t-test

data:  df1 and df2
t = -1.1929, df = 96, p-value = 0.2358
alternative hypothesis: true difference in means is not equal to 0
95 percent confidence interval:
 -0.08723797  0.02174278
sample estimates:
mean of x mean of y 
 2.297286  2.330034 
```

Generate Actual Plots

IVA1

Plotted is the distribution of the intervein region 1 area done manually and compared to MAPPER's automated output. Blue line indicates the mean with the red lines being the 95% CI of the mean.

```
#svg("MAPPERValidationPlots/femaleIVA1Violin.svg", family = "arial", width=3, height=2)

ggplot(melt(femaleValidation_df) %>% dplyr::filter(variable=="aI1" | variable == "mI1"),
       aes(x=factor(variable),
           y=value)) +
  geom_violin(scale="width", alpha=0.50, fill="lightslateblue") +
  geom_jitter(width=0.25) +
  #stat_summary(fun="median", geom="crossbar", size=0.5, fill="blue", color = "blue") +
  #stat_summary(fun="cimed", geom="crossbar", size=0.5, fill="red", color = "red") +
  stat_summary(fun="mean", geom="crossbar", size=0.5, fill="blue", color = "blue") +
  stat_summary(fun="cimean", geom="crossbar", size=0.5, fill="red", color = "red") +
  theme(axis.text.x = element_text(angle = 45, vjust = 0.6),
        axis.title.y=element_blank()) +
  labs(x=expression(paste(""))) +
  theme(panel.grid.major = element_blank(), panel.grid.minor = element_blank(),
panel.background = element_blank(), axis.line = element_line(colour = "black"))
```

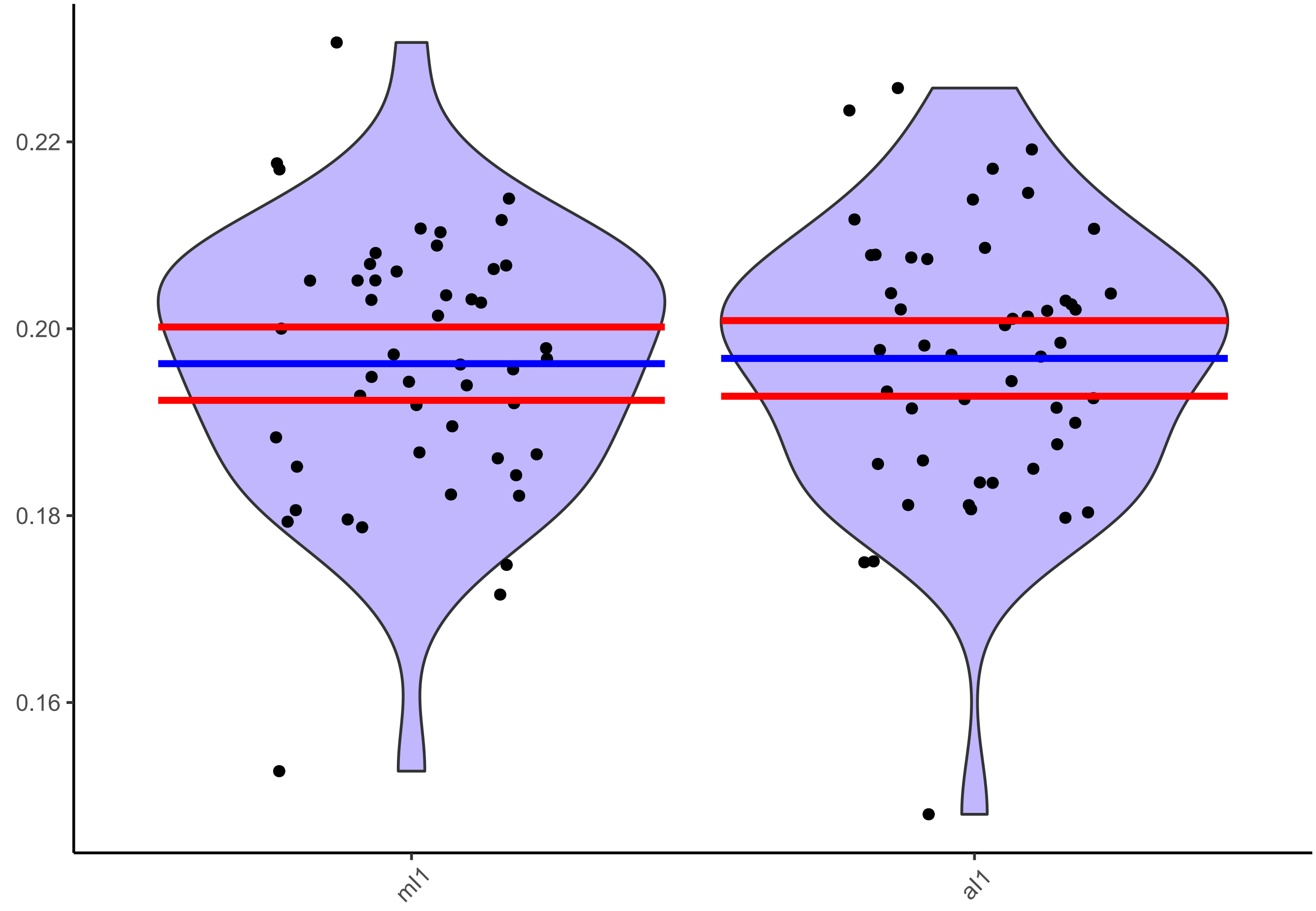

Plotted below is a simple linear fit for MAPPER vs. automated measurements.

```
lmx = femaleValidation_df$aI1
lmy = femaleValidation_df$mI1

#svg("MAPPERValidationPlots/femaleIVA1LM.svg", family = "arial", width=3, height=2)

ggplot(femaleValidation_df,
       aes(x=aI1,
           y=mI1)) +
  geom_point() +
  geom_smooth(method=lm, color="red", fill="lightslateblue", se=TRUE) +
  geom_text(x = min(lmx)*1.10, y = max(lmy)*0.95, label = lm_eqn(lmx, lmy, femaleValidation_df),
parse = TRUE) +
  labs(x=expression(paste("MAPPER Area")), y=expression(paste("Manual Area"))) +
  theme(panel.grid.major = element_blank(), panel.grid.minor = element_blank(),
panel.background = element_blank(), axis.line = element_line(colour = "black"))
```

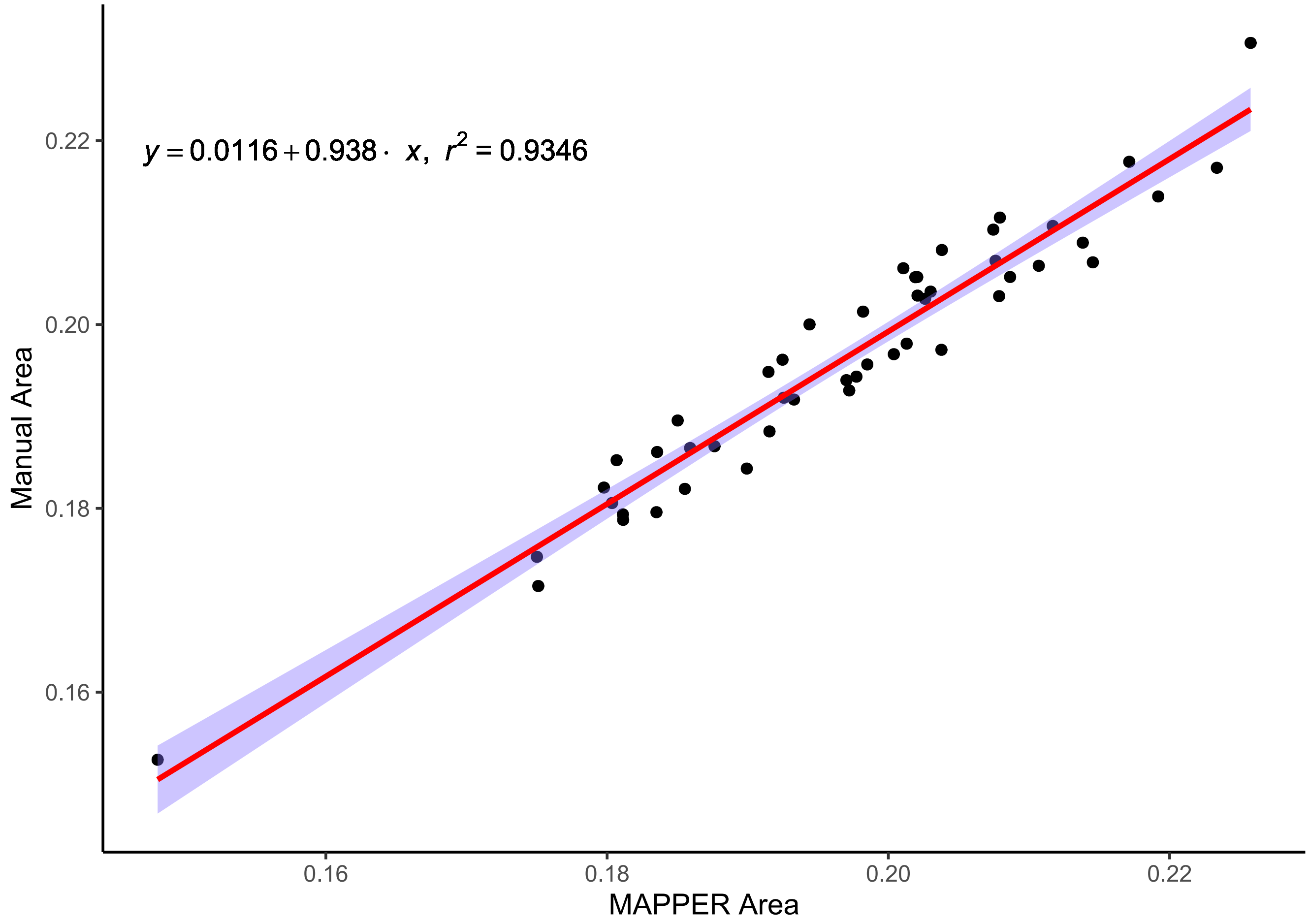

Below is the model summary for the linear fit.

```
model_lm = lm(lmy ~ lmx)
model_lm %>% summary()
```

```

Call:
lm(formula = lmy ~ lmx)

Residuals:
       Min         1Q     Median         3Q        Max 
-0.0061137 -0.0028876 -0.0002299  0.0036025  0.0072288 

Coefficients:
            Estimate Std. Error t value Pr(>|t|)    
(Intercept) 0.011621   0.007141   1.627     0.11    
lmx         0.938104   0.036188  25.923   <2e-16 ***
---
Signif. codes:  0 '***' 0.001 '**' 0.01 '*' 0.05 '.' 0.1 ' ' 1

Residual standard error: 0.003622 on 47 degrees of freedom
Multiple R-squared:  0.9346,	Adjusted R-squared:  0.9332 
F-statistic:   672 on 1 and 47 DF,  p-value: < 2.2e-16
```

Below are the slope parameter bounds.

```
parameter_value = model_lm$coefficients[2]
model_sum = model_lm %>% summary()
parameter_SE = model_sum$coefficients[ , 2][2]
LB_param = parameter_value - qnorm(0.975)*parameter_SE
UB_param = parameter_value + qnorm(0.975)*parameter_SE
cbind(LB_param, UB_param)
```

```
     LB_param UB_param
lmx 0.8671766 1.009031
```

Below is the significance test of the slope parameter for the null hypothesis $H_0 = 1.0$.

```
2*pt((-abs((parameter_value-1.0)/parameter_SE)),model_lm$df.residual)
```

```
       lmx 
0.09378547 
```

IVA2

Plotted is the distribution of the intervein region 2 area done manually and compared to MAPPER's automated output. Blue line indicates the mean with the red lines being the 95% CI of the mean.

```
#svg("MAPPERValidationPlots/femaleIVA2Violin.svg", family = "arial", width=3, height=2)

ggplot(melt(femaleValidation_df) %>% dplyr::filter(variable=="aI2" | variable == "mI2"),
       aes(x=factor(variable),
           y=value)) +
  geom_violin(scale="width", alpha=0.50, fill="lightslateblue") +
  geom_jitter(width=0.25) +
  #stat_summary(fun="median", geom="crossbar", size=0.5, fill="blue", color = "blue") +
  #stat_summary(fun="cimed", geom="crossbar", size=0.5, fill="red", color = "red") +
  stat_summary(fun="mean", geom="crossbar", size=0.5, fill="blue", color = "blue") +
  stat_summary(fun="cimean", geom="crossbar", size=0.5, fill="red", color = "red") +
  theme(axis.text.x = element_text(angle = 45, vjust = 0.6),
        axis.title.y=element_blank()) +
  labs(x=expression(paste(""))) +
  theme(panel.grid.major = element_blank(), panel.grid.minor = element_blank(),
panel.background = element_blank(), axis.line = element_line(colour = "black"))
```

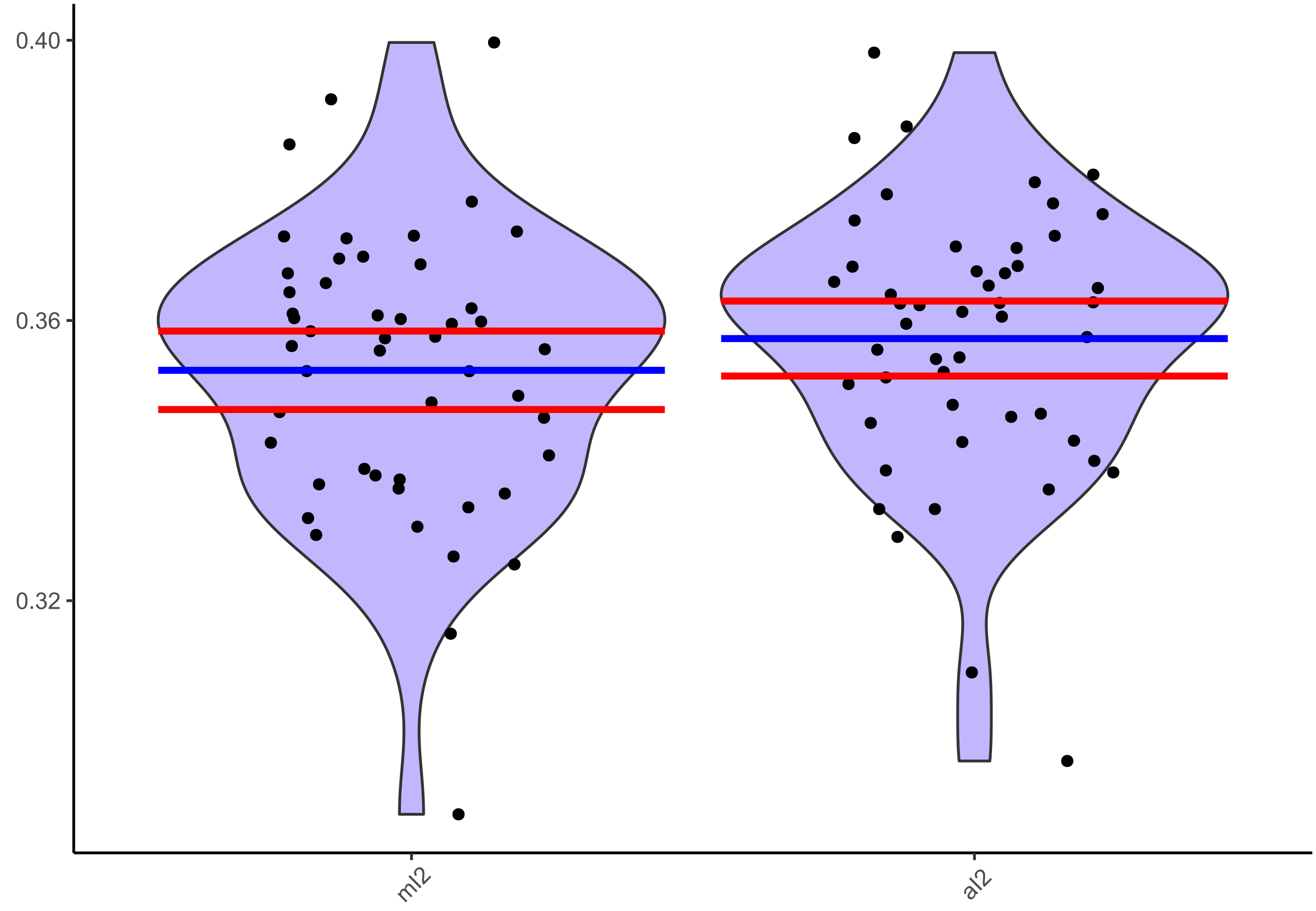

Plotted below is a simple linear fit for MAPPER vs. automated measurements.

```
lmx = femaleValidation_df$aI2
lmy = femaleValidation_df$mI2

#svg("MAPPERValidationPlots/femaleIVA2LM.svg", family = "arial", width=3, height=2)

ggplot(femaleValidation_df,
       aes(x=aI2,
           y=mI2)) +
  geom_point() +
  geom_smooth(method=lm, color="red", fill="lightslateblue", se=TRUE) +
  geom_text(x = min(lmx)*1.10, y = max(lmy)*0.95, label = lm_eqn(lmx, lmy, femaleValidation_df),
parse = TRUE) +
  labs(x=expression(paste("MAPPER Area")), y=expression(paste("Manual Area"))) +
  theme(panel.grid.major = element_blank(), panel.grid.minor = element_blank(),
panel.background = element_blank(), axis.line = element_line(colour = "black"))
```

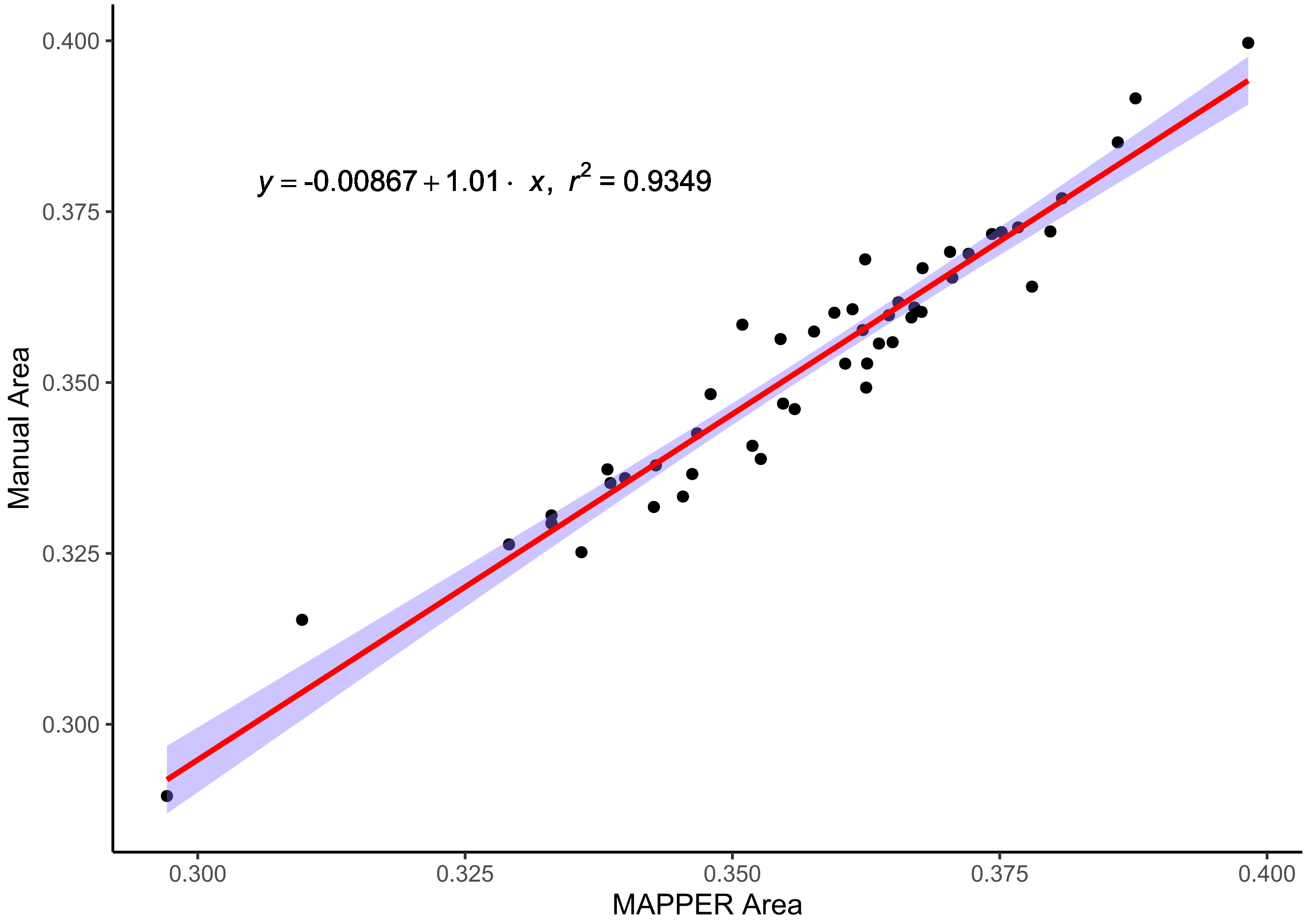

Below is the model summary for the linear fit.

```
model_lm = lm(lmy ~ lmx)
model_lm %>% summary()
```

```
Call:
lm(formula = lmy ~ lmx)

Residuals:
       Min         1Q     Median         3Q        Max 
-0.0096942 -0.0033621  0.0004013  0.0032928  0.0121435 

Coefficients:
             Estimate Std. Error t value Pr(>|t|)    
(Intercept) -0.008672   0.013938  -0.622    0.537    
lmx          1.011582   0.038942  25.977   <2e-16 ***
---
Signif. codes:  0 '***' 0.001 '**' 0.01 '*' 0.05 '.' 0.1 ' ' 1

Residual standard error: 0.005157 on 47 degrees of freedom
Multiple R-squared:  0.9349,    Adjusted R-squared:  0.9335 
F-statistic: 674.8 on 1 and 47 DF,  p-value: < 2.2e-16
```

Below are the slope parameter bounds.

```
parameter_value = model_lm$coefficients[2]
model_sum = model_lm %>% summary()
parameter_SE = model_sum$coefficients[ , 2][2]
LB_param = parameter_value - qnorm(0.975)*parameter_SE
UB_param = parameter_value + qnorm(0.975)*parameter_SE
cbind(LB_param, UB_param)
```

```
     LB_param UB_param
lmx 0.9352577 1.087906
```

Below is the significance test of the slope parameter for the null hypothesis $H_0 = 1.0$.

```
2*pt((-abs((parameter_value-1.0)/parameter_SE)),model_lm$df.residual)
```

```
      lmx 
0.7674611 
```

IVA3

Plotted is the distribution of the intervein region 3 area done manually and compared to MAPPER's automated output. Blue line indicates the mean with the red lines being the 95% CI of the mean.

```
#svg("MAPPERValidationPlots/femaleIVA3Violin.svg", family = "arial", width=3, height=2)

ggplot(melt(femaleValidation_df) %>% dplyr::filter(variable=="aI3" | variable == "mI3"),
       aes(x=factor(variable),
           y=value)) +
  geom_violin(scale="width", alpha=0.50, fill="lightslateblue") +
  geom_jitter(width=0.25) +
  #stat_summary(fun="median", geom="crossbar", size=0.5, fill="blue", color = "blue") +
  #stat_summary(fun="cimed", geom="crossbar", size=0.5, fill="red", color = "red") +
  stat_summary(fun="mean", geom="crossbar", size=0.5, fill="blue", color = "blue") +
  stat_summary(fun="cimean", geom="crossbar", size=0.5, fill="red", color = "red") +
  theme(axis.text.x = element_text(angle = 45, vjust = 0.6),
        axis.title.y=element_blank()) +
  labs(x=expression(paste(""))) +
  theme(panel.grid.major = element_blank(), panel.grid.minor = element_blank(),
panel.background = element_blank(), axis.line = element_line(colour = "black"))
```

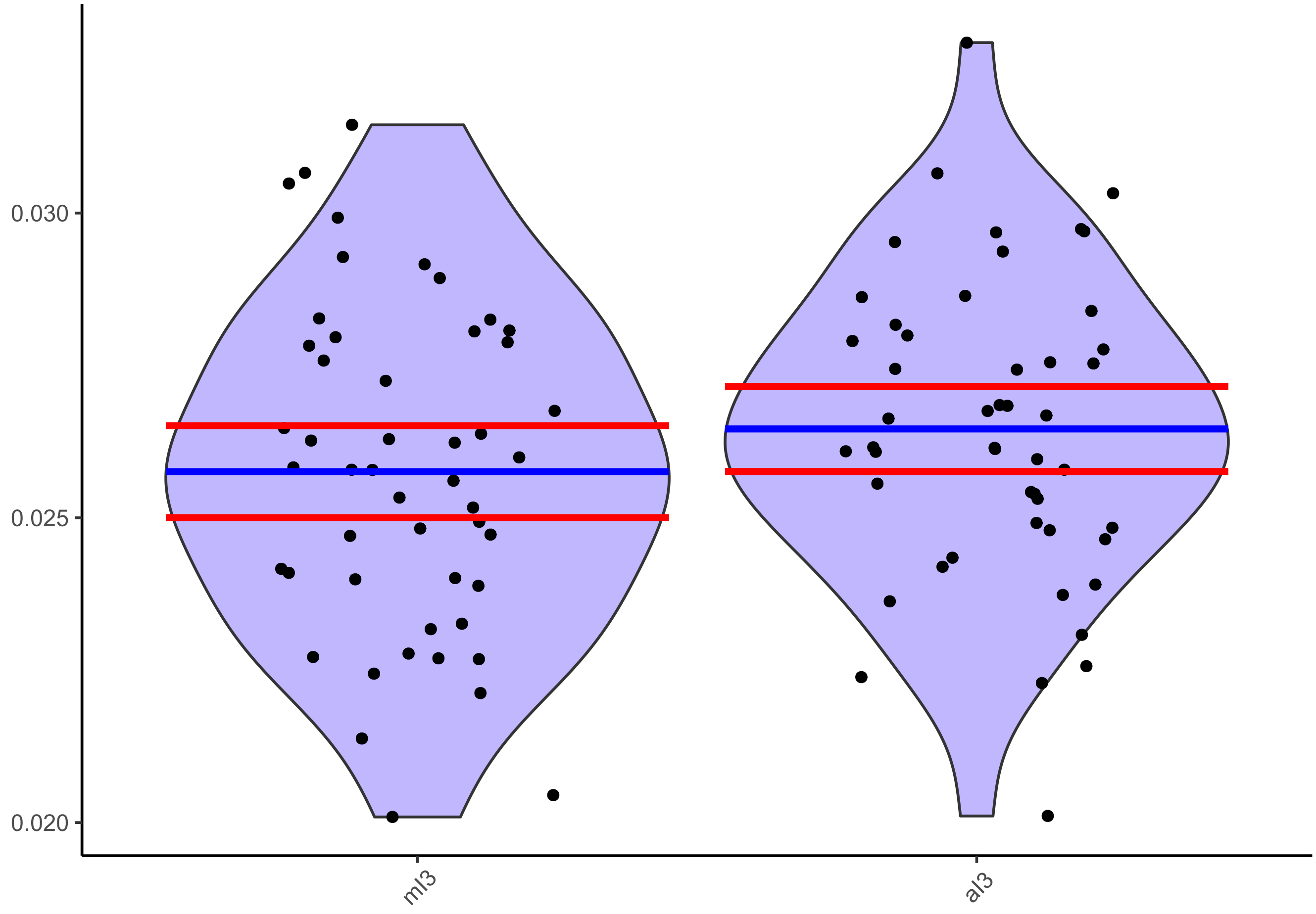

Plotted below is a simple linear fit for MAPPER vs. automated measurements.

```
lmx = femaleValidation_df$aI3
lmy = femaleValidation_df$mI3

#svg("MAPPERValidationPlots/femaleIVA3LM.svg", family = "arial", width=3, height=2)

ggplot(femaleValidation_df,
       aes(x=aI3,
           y=mI3)) +
  geom_point() +
  geom_smooth(method=lm, color="red", fill="lightslateblue", se=TRUE) +
  geom_text(x = min(lmx)*1.10, y = max(lmy)*0.95, label = lm_eqn(lmx, lmy, femaleValidation_df),
parse = TRUE) +
  labs(x=expression(paste("MAPPER Area")), y=expression(paste("Manual Area"))) +
  theme(panel.grid.major = element_blank(), panel.grid.minor = element_blank(),
panel.background = element_blank(), axis.line = element_line(colour = "black"))
```

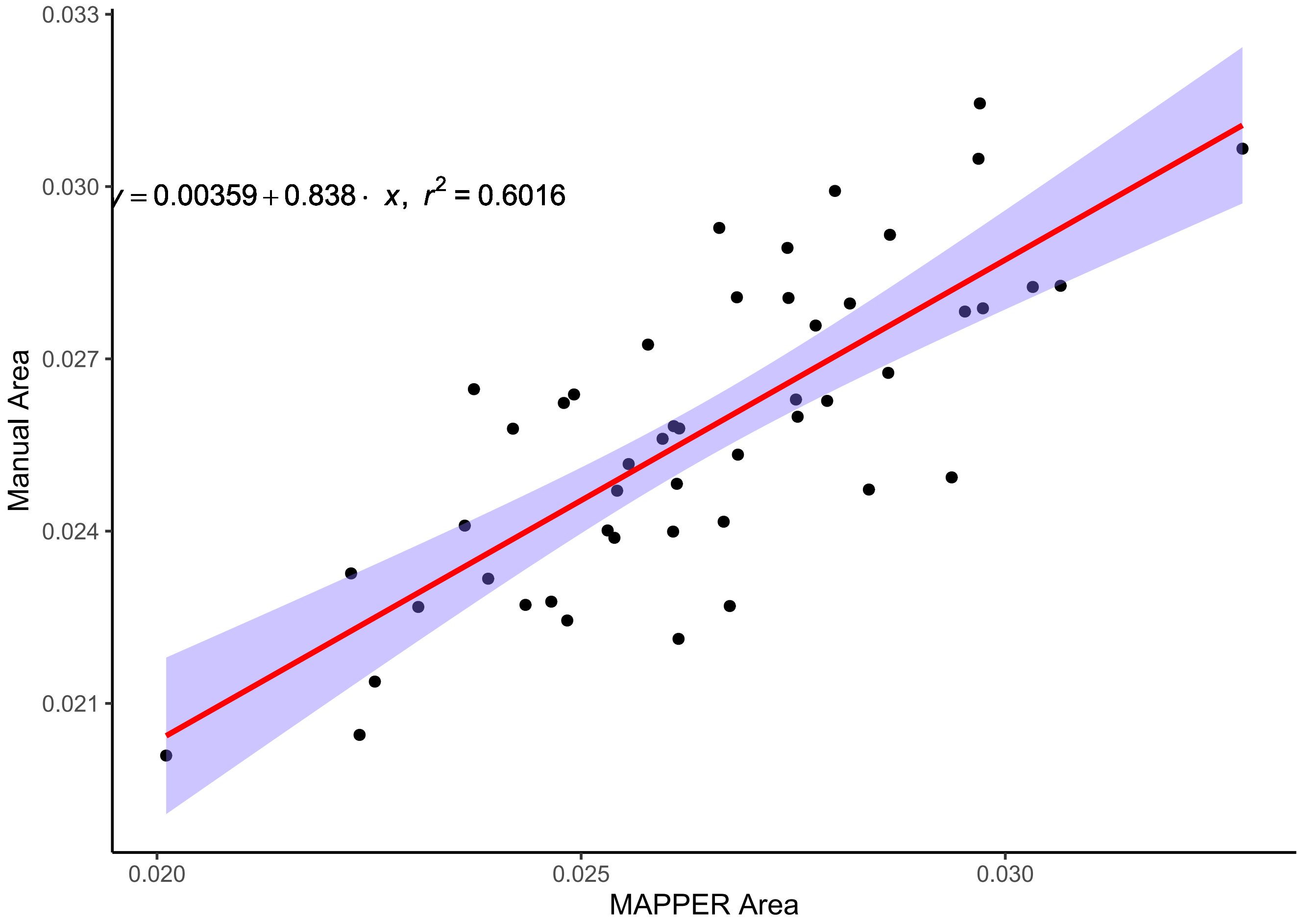

Below is the model summary for the linear fit.

```
model_lm = lm(lmy ~ lmx)
model_lm %>% summary()
```

```
Call:
lm(formula = lmy ~ lmx)

Residuals:
       Min         1Q     Median         3Q        Max 
-0.0033727 -0.0009792 -0.0003644  0.0014773  0.0033811 

Coefficients:
            Estimate Std. Error t value Pr(>|t|)    
(Intercept) 0.003585   0.002643   1.357    0.181    
lmx         0.837972   0.099466   8.425 5.96e-11 ***
---
Signif. codes:  0 '***' 0.001 '**' 0.01 '*' 0.05 '.' 0.1 ' ' 1

Residual standard error: 0.001718 on 47 degrees of freedom
Multiple R-squared:  0.6016,    Adjusted R-squared:  0.5931 
F-statistic: 70.98 on 1 and 47 DF,  p-value: 5.962e-11
```

Below are the slope parameter bounds.

```
parameter_value = model_lm$coefficients[2]
model_sum = model_lm %>% summary()
parameter_SE = model_sum$coefficients[ , 2][2]
LB_param = parameter_value - qnorm(0.975)*parameter_SE
UB_param = parameter_value + qnorm(0.975)*parameter_SE
cbind(LB_param, UB_param)
```

```
    LB_param UB_param
lmx 0.643022 1.032922
```

Below is the significance test of the slope parameter for the null hypothesis $H_0 = 1.0$.

```
2*pt((-abs((parameter_value-1.0)/parameter_SE)),model_lm$df.residual)
```

```
      lmx 
0.1100032 
```

IVA4

Plotted is the distribution of the intervein region 4 area done manually and compared to MAPPER's automated output. Blue line indicates the mean with the red lines being the 95% CI of the mean.

```
#svg("MAPPERValidationPlots/femaleIVA4Violin.svg", family = "arial", width=3, height=2)

ggplot(melt(femaleValidation_df) %>% dplyr::filter(variable=="aI4" | variable == "mI4"),
       aes(x=factor(variable),
           y=value)) +
  geom_violin(scale="width", alpha=0.50, fill="lightslateblue") +
  geom_jitter(width=0.25) +
  #stat_summary(fun="median", geom="crossbar", size=0.5, fill="blue", color = "blue") +
  #stat_summary(fun="cimed", geom="crossbar", size=0.5, fill="red", color = "red") +
  stat_summary(fun="mean", geom="crossbar", size=0.5, fill="blue", color = "blue") +
  stat_summary(fun="cimean", geom="crossbar", size=0.5, fill="red", color = "red") +
  theme(axis.text.x = element_text(angle = 45, vjust = 0.6),
        axis.title.y=element_blank()) +
  labs(x=expression(paste(""))) +
  theme(panel.grid.major = element_blank(), panel.grid.minor = element_blank(),
panel.background = element_blank(), axis.line = element_line(colour = "black"))
```

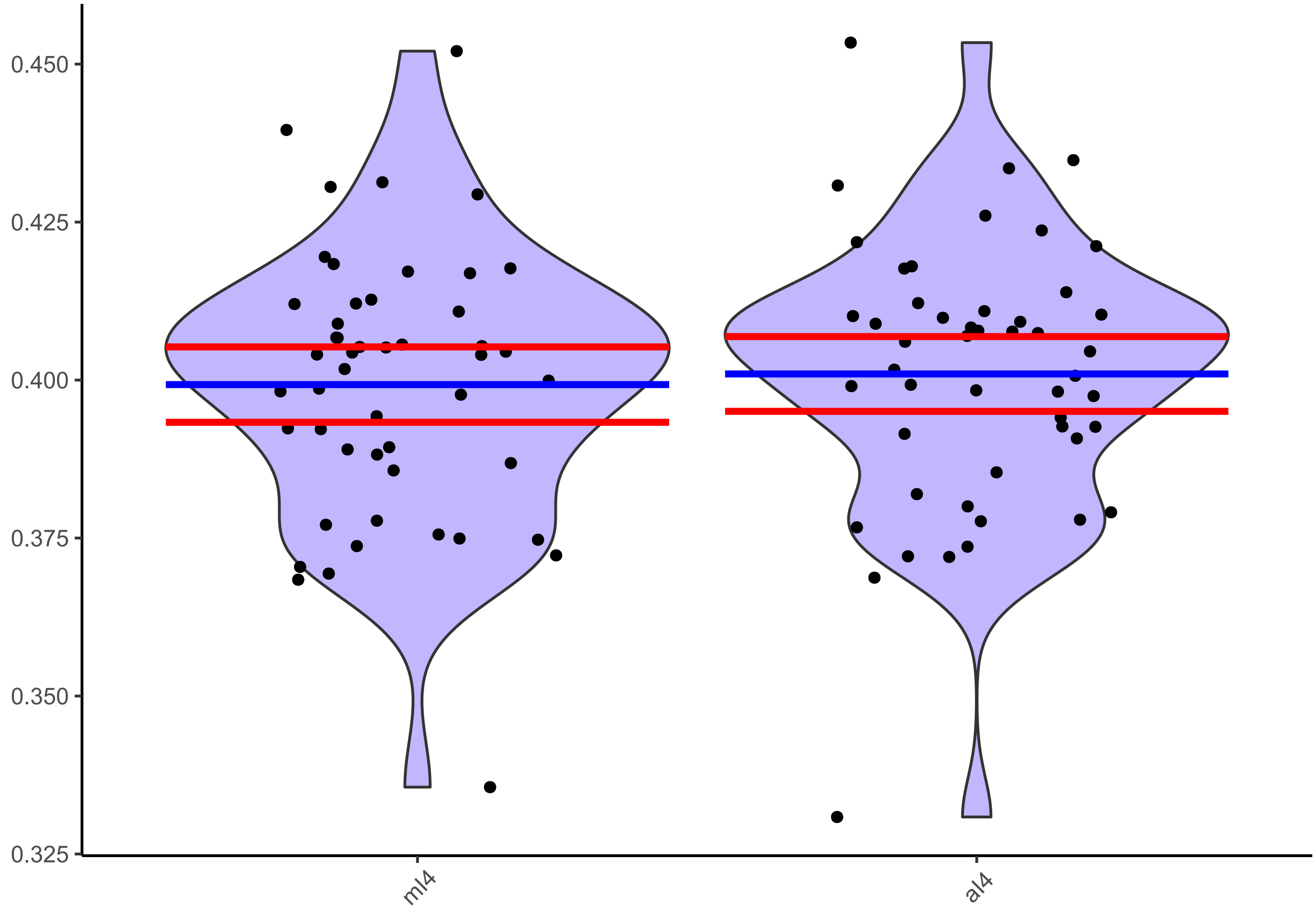

Plotted below is a simple linear fit for MAPPER vs. automated measurements.

```
lmx = femaleValidation_df$aI4
lmy = femaleValidation_df$mI4

#svg("MAPPERValidationPlots/femaleIVA4LM.svg", family = "arial", width=3, height=2)

ggplot(femaleValidation_df,
       aes(x=aI4,
           y=mI4)) +
  geom_point() +
  geom_smooth(method=lm, color="red", fill="lightslateblue", se=TRUE) +
  geom_text(x = min(lmx)*1.10, y = max(lmy)*0.95, label = lm_eqn(lmx, lmy, femaleValidation_df),
parse = TRUE) +
  labs(x=expression(paste("MAPPER Area")), y=expression(paste("Manual Area"))) +
  theme(panel.grid.major = element_blank(), panel.grid.minor = element_blank(),
panel.background = element_blank(), axis.line = element_line(colour = "black"))
```

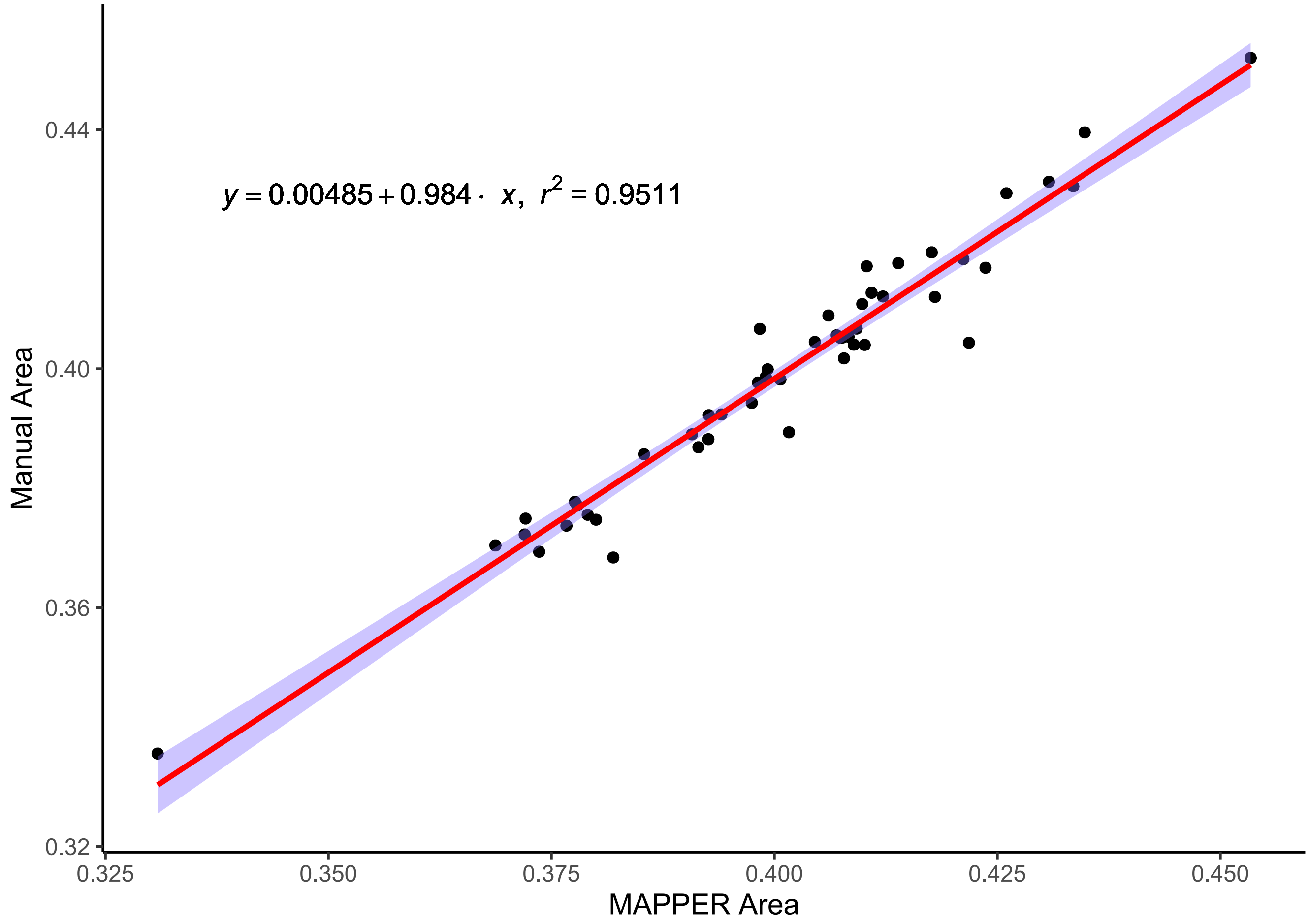

Below is the model summary for the linear fit.

```
model_lm = lm(lmy ~ lmx)
model_lm %>% summary()
```

```
Call:
lm(formula = lmy ~ lmx)

Residuals:
       Min         1Q     Median         3Q        Max 
-0.0154454 -0.0021893  0.0004051  0.0026886  0.0099409 

Coefficients:
            Estimate Std. Error t value Pr(>|t|)    
(Intercept) 0.004845   0.013064   0.371    0.712    
lmx         0.983718   0.032537  30.234   <2e-16 ***
---
Signif. codes:  0 '***' 0.001 '**' 0.01 '*' 0.05 '.' 0.1 ' ' 1

Residual standard error: 0.004761 on 47 degrees of freedom
Multiple R-squared:  0.9511,	Adjusted R-squared:  0.9501 
F-statistic: 914.1 on 1 and 47 DF,  p-value: < 2.2e-16
```

Below are the slope parameter bounds.

```
parameter_value = model_lm$coefficients[2]
model_sum = model_lm %>% summary()
parameter_SE = model_sum$coefficients[ , 2][2]
LB_param = parameter_value - qnorm(0.975)*parameter_SE
UB_param = parameter_value + qnorm(0.975)*parameter_SE
cbind(LB_param, UB_param)
```

```
     LB_param UB_param
lmx 0.9199458  1.04749
```

Below is the significance test of the slope parameter for the null hypothesis $H_0 = 1.0$.

```
2*pt((-abs((parameter_value-1.0)/parameter_SE)),model_lm$df.residual)
```

```
      lmx 
0.6191187 
```

IVA5

Plotted is the distribution of the intervein region 5 area done manually and compared to MAPPER's automated output. Blue line indicates the mean with the red lines being the 95% CI of the mean.

```
#svg("MAPPERValidationPlots/femaleIVA5Violin.svg", family = "arial", width=3, height=2)

ggplot(melt(femaleValidation_df) %>% dplyr::filter(variable=="aI5" | variable == "mI5"),
       aes(x=factor(variable),
           y=value)) +
  geom_violin(scale="width", alpha=0.50, fill="lightslateblue") +
  geom_jitter(width=0.25) +
  #stat_summary(fun="median", geom="crossbar", size=0.5, fill="blue", color = "blue") +
  #stat_summary(fun="cimed", geom="crossbar", size=0.5, fill="red", color = "red") +
  stat_summary(fun="mean", geom="crossbar", size=0.5, fill="blue", color = "blue") +
  stat_summary(fun="cimean", geom="crossbar", size=0.5, fill="red", color = "red") +
  theme(axis.text.x = element_text(angle = 45, vjust = 0.6),
        axis.title.y=element_blank()) +
  labs(x=expression(paste(""))) +
  theme(panel.grid.major = element_blank(), panel.grid.minor = element_blank(),
panel.background = element_blank(), axis.line = element_line(colour = "black"))
```

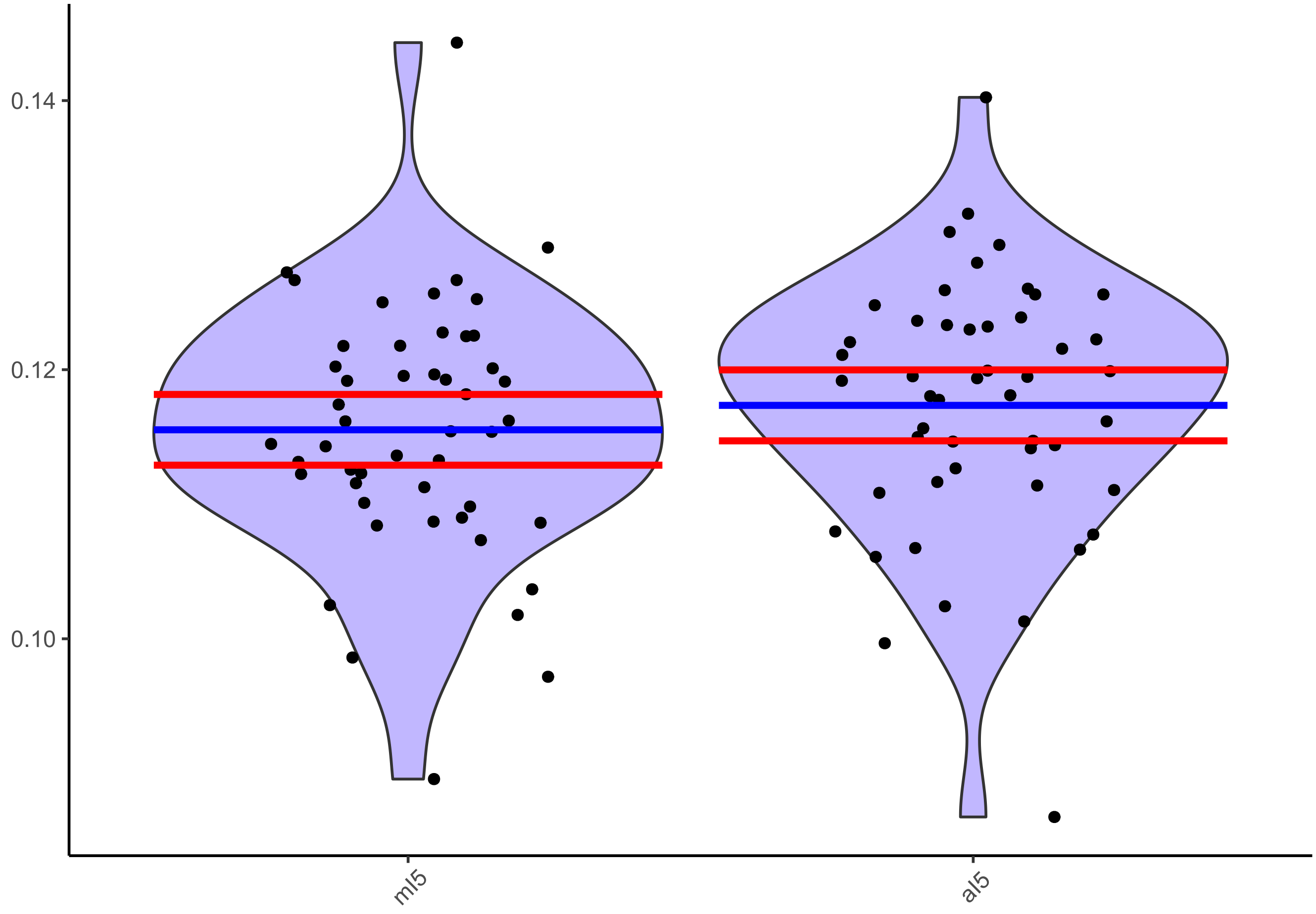

Plotted below is a simple linear fit for MAPPER vs. automated measurements.

```
lmx = femaleValidation_df$aI5
lmy = femaleValidation_df$mI5

#svg("MAPPERValidationPlots/femaleIVA5LM.svg", family = "arial", width=3, height=2)

ggplot(femaleValidation_df,
       aes(x=aI5,
           y=mI5)) +
  geom_point() +
  geom_smooth(method=lm, color="red", fill="lightslateblue", se=TRUE) +
  geom_text(x = min(lmx)*1.10, y = max(lmy)*0.95, label = lm_eqn(lmx, lmy, femaleValidation_df),
parse = TRUE) +
  labs(x=expression(paste("MAPPER Area")), y=expression(paste("Manual Area"))) +
  theme(panel.grid.major = element_blank(), panel.grid.minor = element_blank(),
panel.background = element_blank(), axis.line = element_line(colour = "black"))
```

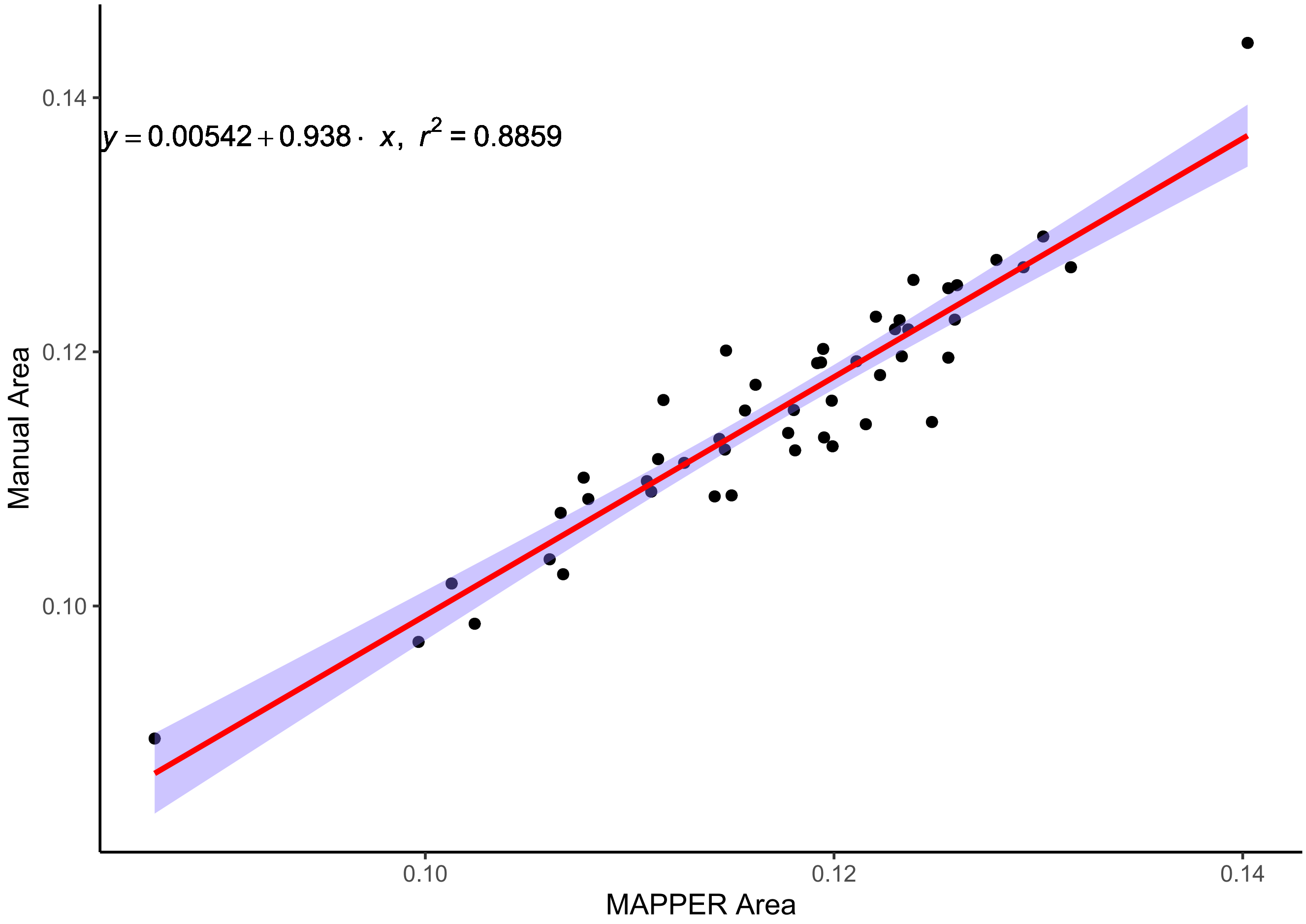

Below is the model summary for the linear fit.

```
model_lm = lm(lmy ~ lmx)
model_lm %>% summary()
```

```

Call:
lm(formula = lmy ~ lmx)

Residuals:
       Min         1Q     Median         3Q        Max 
-0.0080370 -0.0019616  0.0003355  0.0017444  0.0072953 

Coefficients:
            Estimate Std. Error t value Pr(>|t|)    
(Intercept) 0.005417   0.005783   0.937    0.354    
lmx         0.938358   0.049125  19.101   <2e-16 ***
---
Signif. codes:  0 '***' 0.001 '**' 0.01 '*' 0.05 '.' 0.1 ' ' 1

Residual standard error: 0.003206 on 47 degrees of freedom
Multiple R-squared:  0.8859,	Adjusted R-squared:  0.8835 
F-statistic: 364.9 on 1 and 47 DF,  p-value: < 2.2e-16
```

Below are the slope parameter bounds.

```
parameter_value = model_lm$coefficients[2]
model_sum = model_lm %>% summary()
parameter_SE = model_sum$coefficients[ , 2][2]
LB_param = parameter_value - qnorm(0.975)*parameter_SE
UB_param = parameter_value + qnorm(0.975)*parameter_SE
cbind(LB_param, UB_param)
```

```
     LB_param UB_param
lmx 0.8420746  1.03464
```

Below is the significance test of the slope parameter for the null hypothesis $H_0 = 1.0$.

```
2*pt((-abs((parameter_value-1.0)/parameter_SE)),model_lm$df.residual)
```

```
      lmx 
0.2157508 
```

IVA6

Plotted is the distribution of the intervein region 1 area done manually and compared to MAPPER's automated output. Blue line indicates the mean with the red lines being the 95% CI of the mean.

```
#svg("MAPPERValidationPlots/femaleIVA6Violin.svg", family = "arial", width=3, height=2)

ggplot(melt(femaleValidation_df) %>% dplyr::filter(variable=="aI6" | variable == "mI6"),
       aes(x=factor(variable),
           y=value)) +
  geom_violin(scale="width", alpha=0.50, fill="lightslateblue") +
  geom_jitter(width=0.25) +
  #stat_summary(fun="median", geom="crossbar", size=0.5, fill="blue", color = "blue") +
  #stat_summary(fun="cimed", geom="crossbar", size=0.5, fill="red", color = "red") +
  stat_summary(fun="mean", geom="crossbar", size=0.5, fill="blue", color = "blue") +
  stat_summary(fun="cimean", geom="crossbar", size=0.5, fill="red", color = "red") +
  theme(axis.text.x = element_text(angle = 45, vjust = 0.6),
        axis.title.y=element_blank()) +
  labs(x=expression(paste(""))) +
  theme(panel.grid.major = element_blank(), panel.grid.minor = element_blank(),
panel.background = element_blank(), axis.line = element_line(colour = "black"))
```

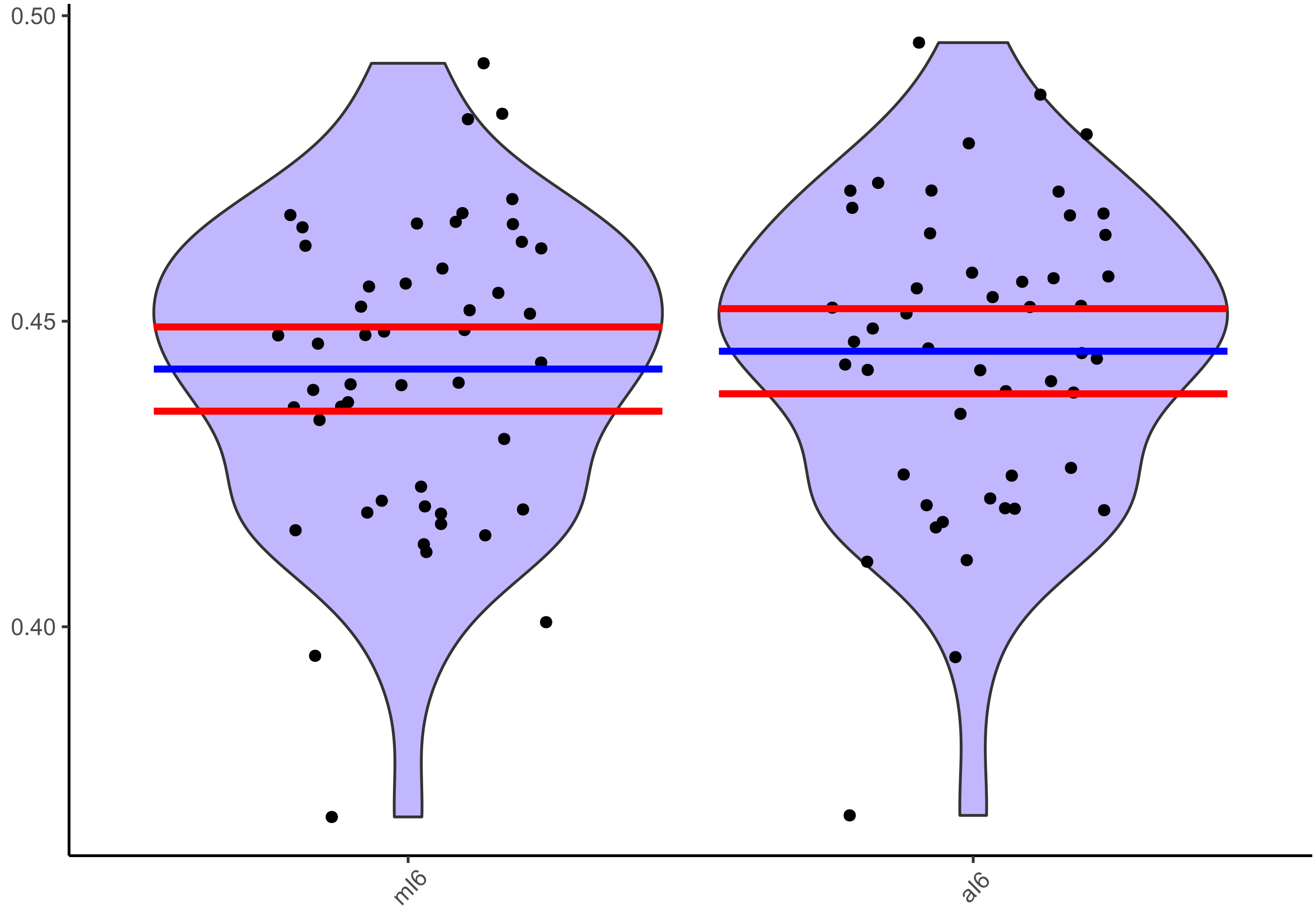

Plotted below is a simple linear fit for MAPPER vs. automated measurements.

```
lmx = femaleValidation_df$aI6
lmy = femaleValidation_df$mI6

#svg("MAPPERValidationPlots/femaleIVA6LM.svg", family = "arial", width=3, height=2)

ggplot(femaleValidation_df,
       aes(x=aI6,
           y=mI6)) +
  geom_point() +
  geom_smooth(method=lm, color="red", fill="lightslateblue", se=TRUE) +
  geom_text(x = min(lmx)*1.10, y = max(lmy)*0.95, label = lm_eqn(lmx, lmy, femaleValidation_df),
parse = TRUE) +
  labs(x=expression(paste("MAPPER Area")), y=expression(paste("Manual Area"))) +
  theme(panel.grid.major = element_blank(), panel.grid.minor = element_blank(),
panel.background = element_blank(), axis.line = element_line(colour = "black"))
```

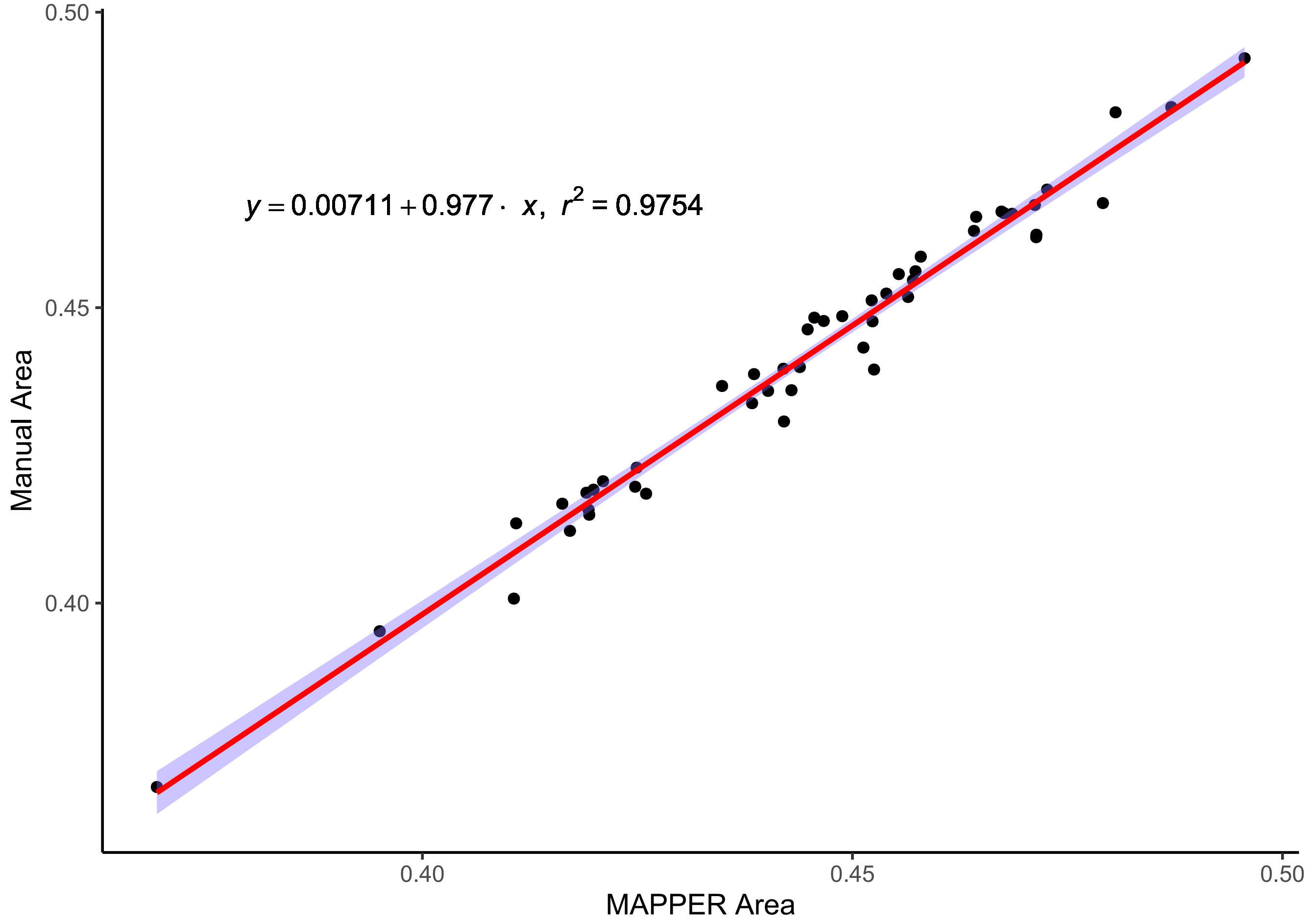

Below is the model summary for the linear fit.

```
model_lm = lm(lmy ~ lmx)
model_lm %>% summary()
```

```

Call:
lm(formula = lmy ~ lmx)

Residuals:
      Min        1Q    Median        3Q       Max 
-0.009894 -0.001730  0.000784  0.002352  0.006191 

Coefficients:
            Estimate Std. Error t value Pr(>|t|)    
(Intercept) 0.007114   0.010085   0.705    0.484    
lmx         0.977459   0.022625  43.203   <2e-16 ***
---
Signif. codes:  0 '***' 0.001 '**' 0.01 '*' 0.05 '.' 0.1 ' ' 1

Residual standard error: 0.0039 on 47 degrees of freedom
Multiple R-squared:  0.9754,    Adjusted R-squared:  0.9749 
F-statistic:  1867 on 1 and 47 DF,  p-value: < 2.2e-16
```

Below are the slope parameter bounds.

```
parameter_value = model_lm$coefficients[2]
model_sum = model_lm %>% summary()
parameter_SE = model_sum$coefficients[ , 2][2]
LB_param = parameter_value - qnorm(0.975)*parameter_SE
UB_param = parameter_value + qnorm(0.975)*parameter_SE
cbind(LB_param, UB_param)
```

```
    LB_param UB_param
lmx 0.933115 1.021802
```

Below is the significance test of the slope parameter for the null hypothesis $H_0 = 1.0$.

```
2*pt((-abs((parameter_value-1.0)/parameter_SE)),model_lm$df.residual)
```

```
      lmx 
0.3241975 
```

IVA7

Plotted is the distribution of the intervein region 1 area done manually and compared to MAPPER's automated output. Blue line indicates the mean with the red lines being the 95% CI of the mean.

```
#svg("MAPPERValidationPlots/femaleIVA7Violin.svg", family = "arial", width=3, height=2)

ggplot(melt(femaleValidation_df) %>% dplyr::filter(variable=="aI7" | variable == "mI7"),
       aes(x=factor(variable),
           y=value)) +
  geom_violin(scale="width", alpha=0.50, fill="lightslateblue") +
  geom_jitter(width=0.25) +
  stat_summary(fun="median", geom="crossbar", size=0.5, fill="blue", color = "blue") +
  stat_summary(fun="cimed", geom="crossbar", size=0.5, fill="red", color = "red") +
  #stat_summary(fun="mean", geom="crossbar", size=0.5, fill="blue", color = "blue") +
  #stat_summary(fun="cimean", geom="crossbar", size=0.5, fill="red", color = "red") +
  theme(axis.text.x = element_text(angle = 45, vjust = 0.6),
        axis.title.y=element_blank()) +
  labs(x=expression(paste(""))) +
  theme(panel.grid.major = element_blank(), panel.grid.minor = element_blank(),
panel.background = element_blank(), axis.line = element_line(colour = "black"))
```

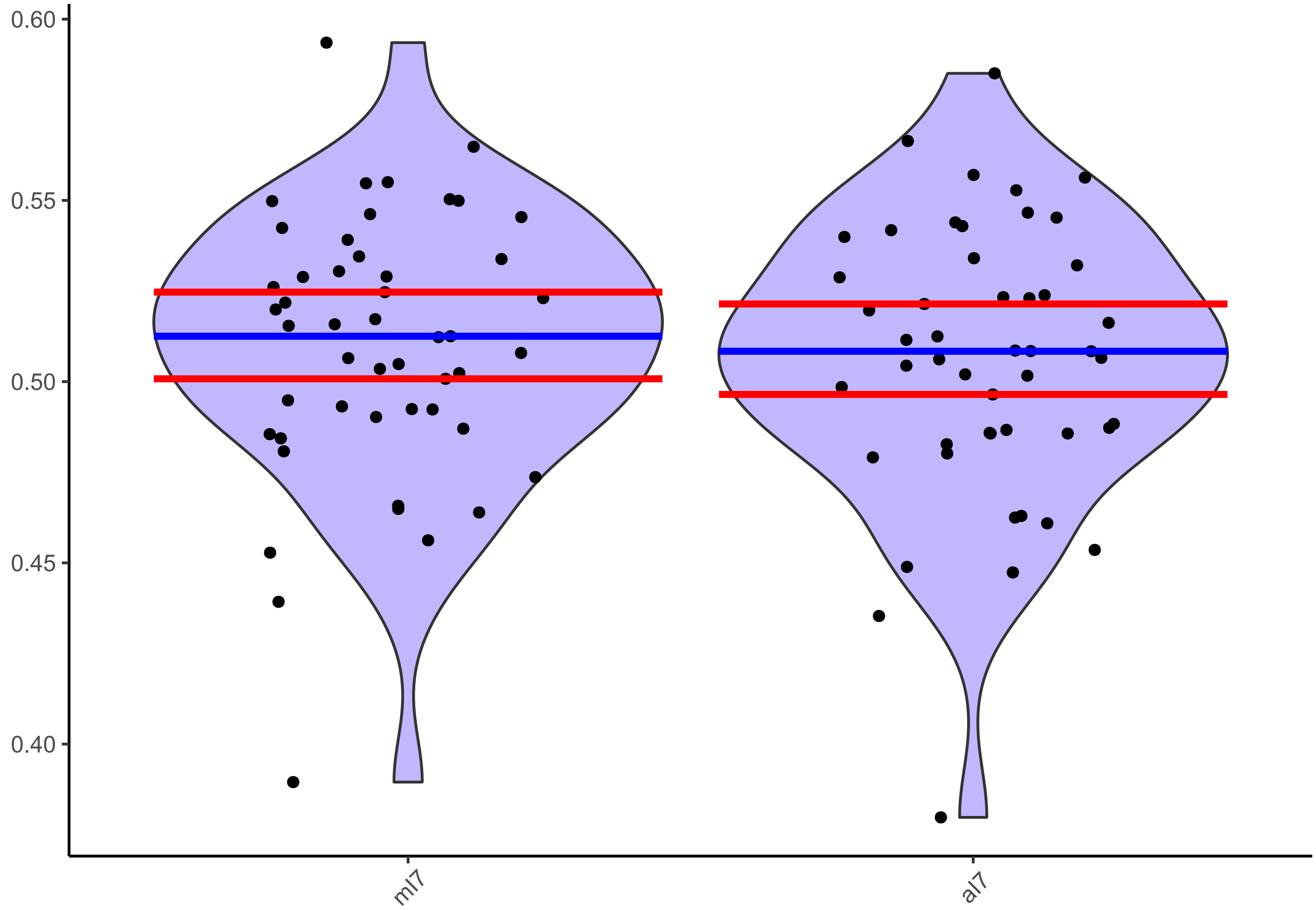

Plotted below is a simple linear fit for MAPPER vs. automated measurements.

```
lmx = femaleValidation_df$aI7
lmy = femaleValidation_df$mI7

#svg("MAPPERValidationPlots/femaleIVA7LM.svg", family = "arial", width=3, height=2)

ggplot(femaleValidation_df,
       aes(x=aI7,
           y=mI7)) +
  geom_point() +
  geom_smooth(method=lm, color="red", fill="lightslateblue", se=TRUE) +
  geom_text(x = min(lmx)*1.10, y = max(lmy)*0.95, label = lm_eqn(lmx, lmy, femaleValidation_df),
parse = TRUE) +
  labs(x=expression(paste("MAPPER Area")), y=expression(paste("Manual Area"))) +
  theme(panel.grid.major = element_blank(), panel.grid.minor = element_blank(),
panel.background = element_blank(), axis.line = element_line(colour = "black"))
```

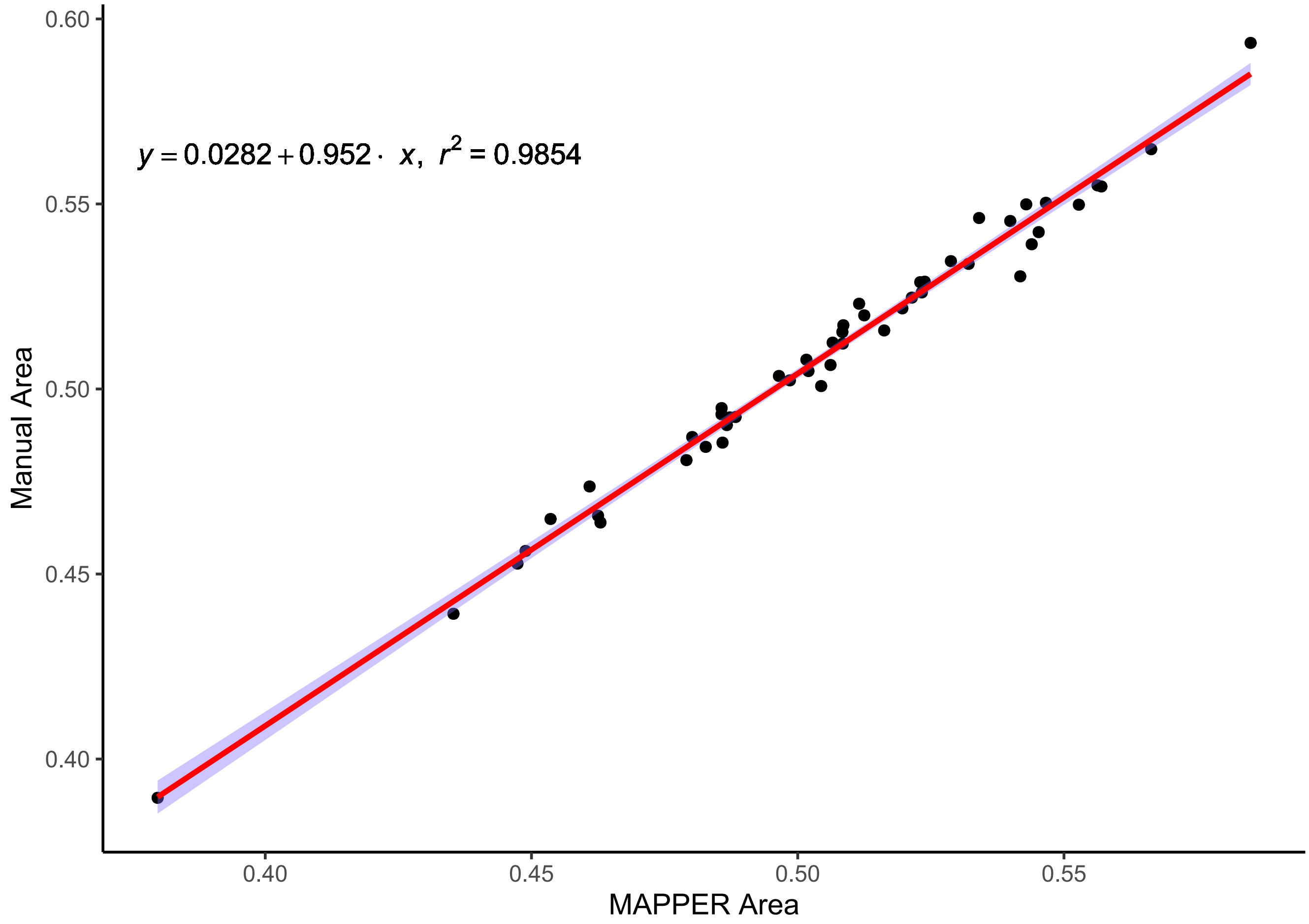

Below is the model summary for the linear fit.

```
model_lm = lm(lmy ~ lmx)
model_lm %>% summary()
```

```

Call:
lm(formula = lmy ~ lmx)

Residuals:
       Min         1Q     Median         3Q        Max 
-0.0135075 -0.0033584 -0.0002064  0.0027914  0.0096174 

Coefficients:
            Estimate Std. Error t value Pr(>|t|)    
(Intercept) 0.028166   0.008574   3.285  0.00193 ** 
lmx         0.952006   0.016908  56.306  < 2e-16 ***
---
Signif. codes:  0 '***' 0.001 '**' 0.01 '*' 0.05 '.' 0.1 ' ' 1

Residual standard error: 0.004463 on 47 degrees of freedom
Multiple R-squared:  0.9854,    Adjusted R-squared:  0.9851 
F-statistic:  3170 on 1 and 47 DF,  p-value: < 2.2e-16
```

Below are the slope parameter bounds.

```
parameter_value = model_lm$coefficients[2]
model_sum = model_lm %>% summary()
parameter_SE = model_sum$coefficients[ , 2][2]
LB_param = parameter_value - qnorm(0.975)*parameter_SE
UB_param = parameter_value + qnorm(0.975)*parameter_SE
cbind(LB_param, UB_param)
```

```
     LB_param  UB_param
lmx 0.9188675 0.9851451
```

Below is the significance test of the slope parameter for the null hypothesis $H_0 = 1.0$.

```
2*pt((-abs((parameter_value-1.0)/parameter_SE)),model_lm$df.residual)
```

```
        lmx 
0.006672622 
```

Total Area

Plotted is the distribution of the total wing area done manually and compared to MAPPER's automated output. Blue line indicates the mean with the red lines being the 95% CI of the mean.

```
#svg("MAPPERValidationPlots/femaleIVATotViolin.svg", family = "arial", width=3, height=2)

ggplot(melt(femaleValidation_df) %>% dplyr::filter(variable=="aTA" | variable == "mTA"),
       aes(x=factor(variable),
           y=value)) +
  geom_violin(scale="width", alpha=0.50, fill="lightslateblue") +
  geom_jitter(width=0.25) +
  #stat_summary(fun="median", geom="crossbar", size=0.5, fill="blue", color = "blue") +
  #stat_summary(fun="cimed", geom="crossbar", size=0.5, fill="red", color = "red") +
  stat_summary(fun="mean", geom="crossbar", size=0.5, fill="blue", color = "blue") +
  stat_summary(fun="cimean", geom="crossbar", size=0.5, fill="red", color = "red") +
  theme(axis.text.x = element_text(angle = 45, vjust = 0.6),
        axis.title.y=element_blank()) +
  labs(x=expression(paste(""))) +
  theme(panel.grid.major = element_blank(), panel.grid.minor = element_blank(),
panel.background = element_blank(), axis.line = element_line(colour = "black"))
```

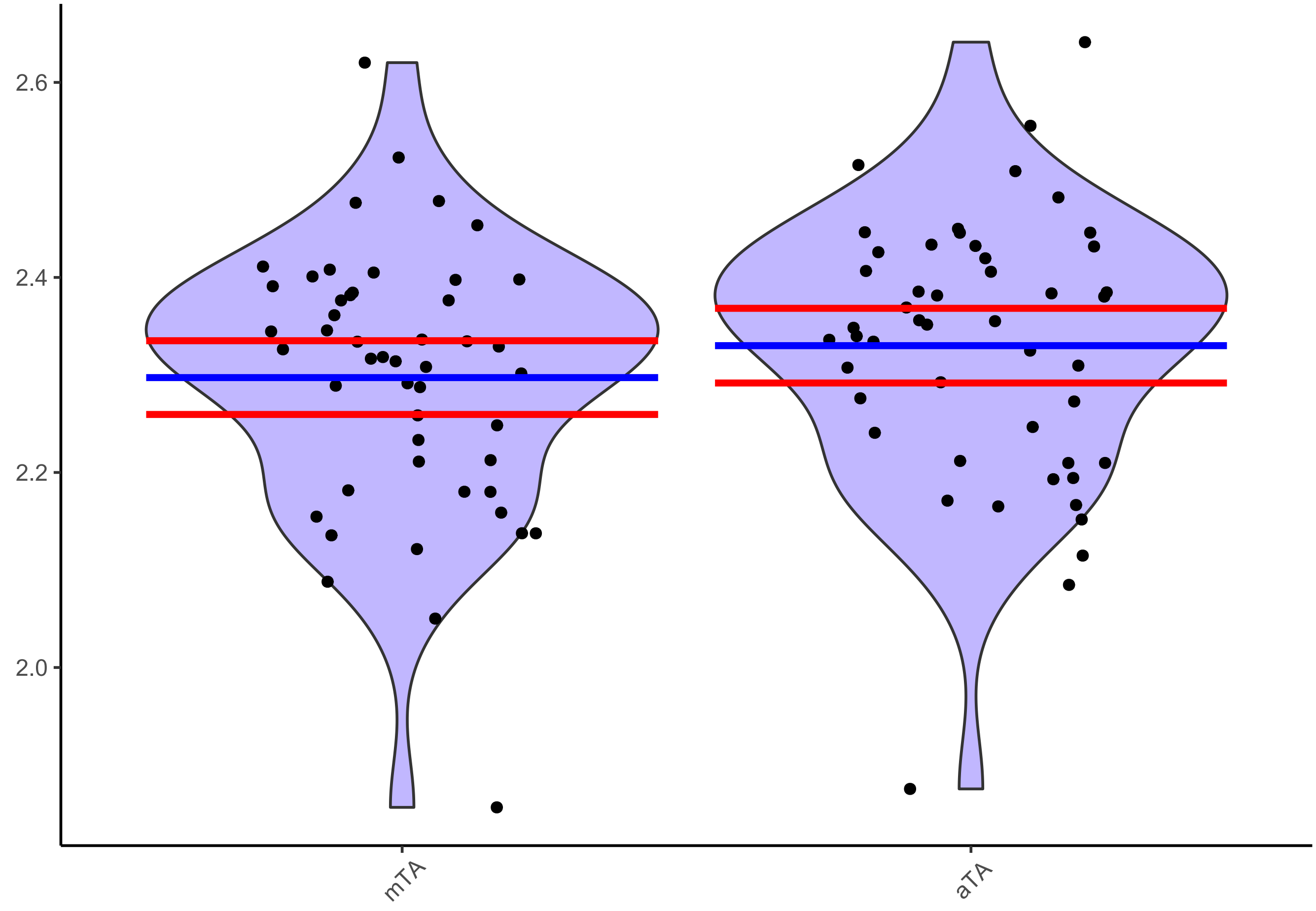

Plotted below is a simple linear fit for MAPPER vs. automated measurements.

```
lmx = femaleValidation_df$aTA
lmy = femaleValidation_df$mTA

#svg("MAPPERValidationPlots/femaleIVATotLM.svg", family = "arial", width=3, height=2)

ggplot(femaleValidation_df,
       aes(x=aTA,
           y=mTA)) +
  geom_point() +
  geom_smooth(method=lm, color="red", fill="lightslateblue", se=TRUE) +
  geom_text(x = min(lmx)*1.10, y = max(lmy)*0.95, label = lm_eqn(lmx, lmy, femaleValidation_df),
parse = TRUE) +
  labs(x=expression(paste("MAPPER Area")), y=expression(paste("Manual Area"))) +
  theme(panel.grid.major = element_blank(), panel.grid.minor = element_blank(),
panel.background = element_blank(), axis.line = element_line(colour = "black"))
```

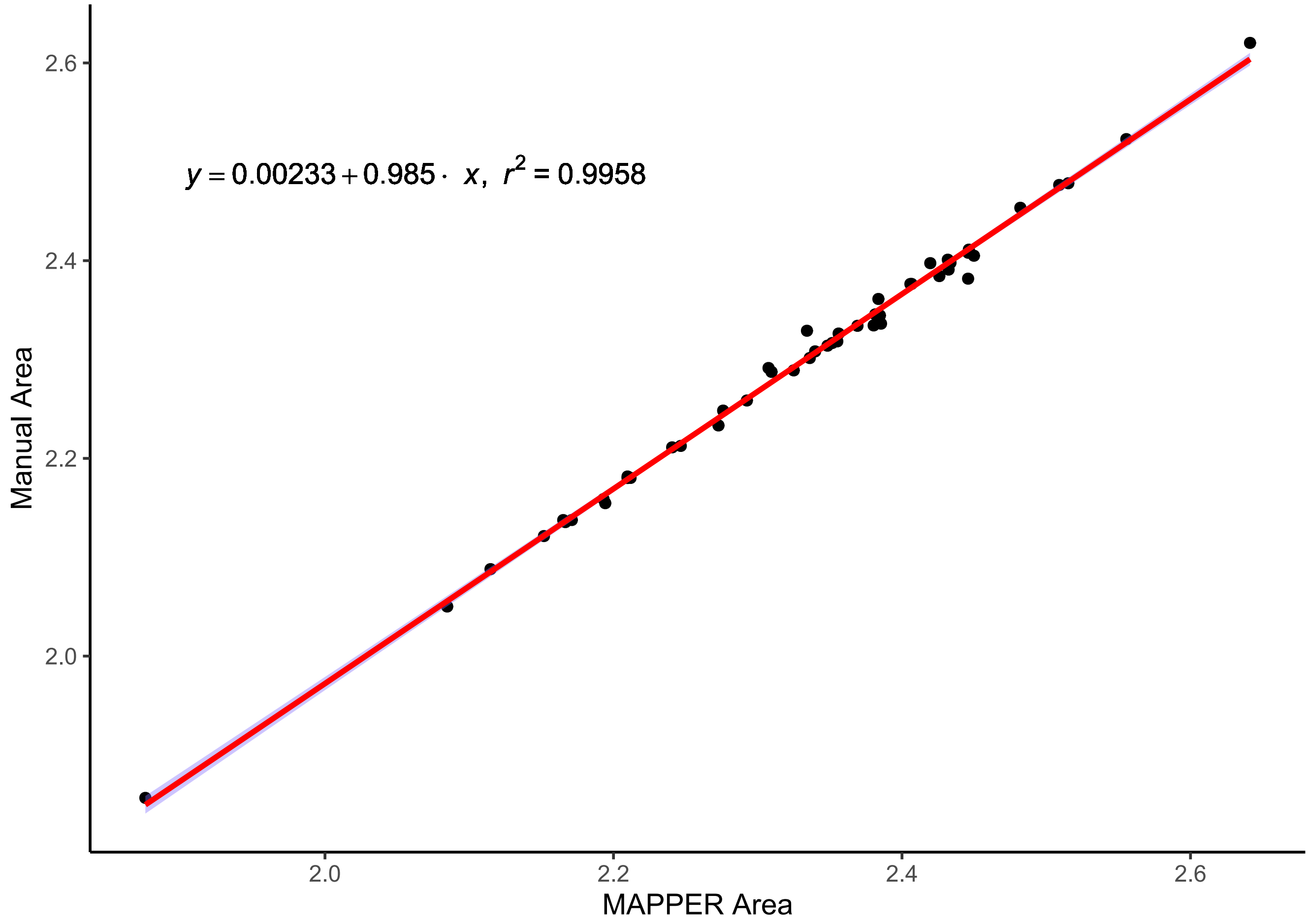

Below is the model summary for the linear fit.

```
model_lm = lm(lmy ~ lmx)
model_lm %>% summary()
```

```

Call:
lm(formula = lmy ~ lmx)

Residuals:
       Min         1Q     Median         3Q        Max 
-0.0295573 -0.0034398 -0.0008122  0.0034989  0.0277932 

Coefficients:
            Estimate Std. Error t value Pr(>|t|)    
(Intercept) 0.002328   0.021807   0.107    0.915    
lmx         0.984946   0.009343 105.417   <2e-16 ***
---
Signif. codes:  0 '***' 0.001 '**' 0.01 '*' 0.05 '.' 0.1 ' ' 1

Residual standard error: 0.008853 on 47 degrees of freedom
Multiple R-squared:  0.9958,    Adjusted R-squared:  0.9957 
F-statistic: 1.111e+04 on 1 and 47 DF,  p-value: < 2.2e-16
```

Below are the slope parameter bounds.

```
parameter_value = model_lm$coefficients[2]
model_sum = model_lm %>% summary()
parameter_SE = model_sum$coefficients[ , 2][2]
LB_param = parameter_value - qnorm(0.975)*parameter_SE
UB_param = parameter_value + qnorm(0.975)*parameter_SE
cbind(LB_param, UB_param)
```

```
     LB_param UB_param
lmx 0.9666338 1.003259
```

Below is the significance test of the slope parameter for the null hypothesis $H_0 = 1.0$.

```
2*pt((-abs((parameter_value-1.0)/parameter_SE)),model_lm$df.residual)
```

```
     lmx 
0.113842 
```

Multiple independent runs of MAPPER

Load repeated trials

```
samarkand_1 = read.csv("Samarkand_1.csv", header=T, sep=",") %>% tibble()
samarkand_2 = read.csv("Samarkand_2.csv", header=T, sep=",") %>% tibble()
samarkand_3 = read.csv("Samarkand_3.csv", header=T, sep=",") %>% tibble()
```

View the dataframes

```
samarkand_1
```

Wing.name <chr>	Genot… <int>	Intervein.area_1 <dbl>	Intervein.area_2 <dbl>	Intervein.area_3 <dbl>
samw_F_L_oly_4X_12	1	0.207876	0.374276	0.029736
samw_F_L_oly_4X_13	1	0.194392	0.367684	0.026156
samw_F_L_oly_4X_14	1	0.183556	0.351868	0.024916
samw_F_L_oly_4X_16	1	0.189932	0.366740	0.025560
samw_F_L_oly_4X_17	1	0.181140	0.335880	0.024196
samw_F_L_oly_4X_18	1	0.197216	0.364980	0.027992
samw_F_L_oly_4X_19	1	0.201064	0.365520	0.027552
samw_F_L_oly_4X_20	1	0.210696	0.386040	0.029684
samw_F_L_oly_4X_21	1	0.181120	0.342652	0.023736
samw_F_L_oly_4X_22	1	0.191472	0.350928	0.027432

1-10 of 62 rows | 1-6 of 27 columns Previous **1** 2 3 4 5 6 7 Next

```
samarkand_2$Genotype = 2
samarkand_2
```

Wing.name <chr>	Genot… <dbl>	Intervein.area_1 <dbl>	Intervein.area_2 <dbl>	Intervein.area_3 <dbl>
samw_F_L_oly_4X_12	2	0.222112	0.383456	0.029676
samw_F_L_oly_4X_13	2	0.204548	0.374880	0.022376
samw_F_L_oly_4X_14	2	0.197208	0.359524	0.024544
samw_F_L_oly_4X_16	2	0.200804	0.374716	0.022600
samw_F_L_oly_4X_17	2	0.192028	0.342700	0.023472
samw_F_L_oly_4X_18	2	0.210188	0.370912	0.027088
samw_F_L_oly_4X_19	2	0.224496	0.379740	0.027848
samw_F_L_oly_4X_20	2	0.223572	0.396260	0.029052
samw_F_L_oly_4X_21	2	0.197884	0.353472	0.024088
samw_F_L_oly_4X_22	2	0.211192	0.368356	0.026508

1-10 of 69 rows | 1-6 of 27 columns Previous **1** 2 3 4 5 6 7 Next

```
samarkand_3$Genotype = 3
samarkand_3
```

Wing.name <chr>	Genot… <dbl>	Intervein.area_1 <dbl>	Intervein.area_2 <dbl>	Intervein.area_3 <dbl>
samw_F_L_oly_4X_12	3	0.219080	0.383904	0.032220
samw_F_L_oly_4X_13	3	0.205408	0.376140	0.026908
samw_F_L_oly_4X_14	3	0.197804	0.362588	0.026640
samw_F_L_oly_4X_16	3	0.201152	0.375848	0.024596
samw_F_L_oly_4X_17	3	0.194300	0.346816	0.025948
samw_F_L_oly_4X_18	3	0.211440	0.374976	0.028644
samw_F_L_oly_4X_19	3	0.220012	0.383832	0.030108
samw_F_L_oly_4X_20	3	0.223028	0.397064	0.030704
samw_F_L_oly_4X_21	3	0.199452	0.360456	0.025588
samw_F_L_oly_4X_22	3	0.210316	0.374980	0.028824

1-10 of 69 rows | 1-6 of 27 columns Previous **1** 2 3 4 5 6 7 Next

Join the dataframes to create a single dataframe with all samples

```
join_1 = inner_join(samarkand_1, samarkand_2, by="Wing.name", suffix=c(".run1", ".run2"))
join_1
```

Wing.name <chr>	Genotype.run1 <int>	Intervein.area_1.run1 <dbl>	Intervein.area_2.run1 <dbl>
samw_F_L_oly_4X_12	1	0.207876	0.374276
samw_F_L_oly_4X_13	1	0.194392	0.367684
samw_F_L_oly_4X_14	1	0.183556	0.351868
samw_F_L_oly_4X_16	1	0.189932	0.366740
samw_F_L_oly_4X_17	1	0.181140	0.335880
samw_F_L_oly_4X_18	1	0.197216	0.364980
samw_F_L_oly_4X_19	1	0.201064	0.365520
samw_F_L_oly_4X_20	1	0.210696	0.386040

Wing.name <chr>	Genotype.run1 <int>	Intervein.area_1.run1 <dbl>	Intervein.area_2.run1 <dbl>
samw_F_L_oly_4X_21	1	0.181120	0.342652
samw_F_L_oly_4X_22	1	0.191472	0.350928

1-10 of 61 rows | 1-4 of 53 columns Previous **1** 2 3 4 5 6 7 Next

```
join_2 = inner_join(join_1, samarkand_3, by="Wing.name")
join_2
```

Wing.name <chr>	Genotype.run1 <int>	Intervein.area_1.run1 <dbl>	Intervein.area_2.run1 <dbl>
samw_F_L_oly_4X_12	1	0.207876	0.374276
samw_F_L_oly_4X_13	1	0.194392	0.367684
samw_F_L_oly_4X_14	1	0.183556	0.351868
samw_F_L_oly_4X_16	1	0.189932	0.366740
samw_F_L_oly_4X_17	1	0.181140	0.335880
samw_F_L_oly_4X_18	1	0.197216	0.364980
samw_F_L_oly_4X_19	1	0.201064	0.365520
samw_F_L_oly_4X_20	1	0.210696	0.386040
samw_F_L_oly_4X_21	1	0.181120	0.342652
samw_F_L_oly_4X_22	1	0.191472	0.350928

1-10 of 61 rows | 1-4 of 79 columns Previous **1** 2 3 4 5 6 7 Next

Total Wing Area across all independent MAPPER runs and manual measurements

Statistical comparisons

```
first_group = melt(join_2) %>% dplyr::filter(variable=="Total.wing.area.run1") %>% mutate(variab
le=1)
second_group = melt(join_2) %>% dplyr::filter(variable=="Total.wing.area.run2") %>% mutate(varia
ble=2)
third_group = melt(join_2) %>% dplyr::filter(variable=="Total.wing.area") %>% mutate(variable=3)


comparison_df = bind_rows(first_group, second_group, third_group)
comparison_df
```

Wing.name <chr>	variable <dbl>	value <dbl>
samw_F_L_oly_4X_12	1	2.406632
samw_F_L_oly_4X_13	1	2.380364
samw_F_L_oly_4X_14	1	2.272792
samw_F_L_oly_4X_16	1	2.309568
samw_F_L_oly_4X_17	1	2.165168
samw_F_L_oly_4X_18	1	2.351600
samw_F_L_oly_4X_19	1	2.419604
samw_F_L_oly_4X_20	1	2.515296
samw_F_L_oly_4X_21	1	2.209732
samw_F_L_oly_4X_22	1	2.334128

1-10 of 183 rows Previous **1** 2 3 4 5 6 ... 19 Next

Check normality assumptions

Data is not normal, by Shapiro-Wilk's test, thus we must proceed with Levene's test.

```
testSelection = comparison_df %>% dplyr::filter(variable==1)
shapiro.test(testSelection$value)
```

```

	Shapiro-Wilk normality test

data:  testSelection$value
W = 0.92922, p-value = 0.001665
```

```
testSelection = comparison_df %>% dplyr::filter(variable==2)
shapiro.test(testSelection$value)
```

```

	Shapiro-Wilk normality test

data:  testSelection$value
W = 0.93484, p-value = 0.002914
```

```
testSelection = comparison_df %>% dplyr::filter(variable==3)
shapiro.test(testSelection$value)
```

```
	Shapiro-Wilk normality test

data:  testSelection$value
W = 0.91983, p-value = 0.0006768
```

Levene's test for equal variance of non-parametric distribution

Because the data is not normally distributed, we must use Levene's test for evaluating homogeneity of variances. More information can be found Here (https://www.itl.nist.gov/div898/handbook/eda/section3/eda35a.htm) and Here (http://www.sthda.com/english/wiki/compare-multiple-sample-variances-in-r). There is no statistical difference in variances of the independent MAPPER runs.

```
leveneTest(value~factor(variable), data=comparison_df)
```

	Df <int>	F value <dbl>	Pr(>F) <dbl>
group	2	0.02651616	0.9738361
	180	NA	NA

2 rows

Kruskal-Wallis test for non-parametric ANOVA

Because the data is not normally distributed, we must use the Kruskal-Wallis test for evaluating equalty of the means. More information can be found Here (http://www.sthda.com/english/wiki/kruskal-wallis-test-in-r). There is no statistical difference in means of the independent MAPPER runs.

```
kruskal.test(value~factor(variable), data=comparison_df)
```

```
	Kruskal-Wallis rank sum test

data:  value by factor(variable)
Kruskal-Wallis chi-squared = 0.98642, df = 2, p-value = 0.6107
```

Compare to manual

Create a dataframe that joins manual measurements to the independent MAPPER measurements.

```
femaleValidation_df_2 = femaleValidation_df %>% dplyr::rename(Wing.name=Filename)
join_3 = inner_join(join_2, femaleValidation_df_2, by="Wing.name")
join_3
```

Wing.name <chr>	Genotype.run1 <int>	Intervein.area_1.run1 <dbl>	Intervein.area_2.run1 <dbl>

Wing.name <chr>	Genotype.run1 <int>	Intervein.area_1.run1 <dbl>	Intervein.area_2.run1 <dbl>
samw_F_L_oly_4X_12	1	0.207876	0.374276
samw_F_L_oly_4X_13	1	0.194392	0.367684
samw_F_L_oly_4X_14	1	0.183556	0.351868
samw_F_L_oly_4X_16	1	0.189932	0.366740
samw_F_L_oly_4X_17	1	0.181140	0.335880
samw_F_L_oly_4X_18	1	0.197216	0.364980
samw_F_L_oly_4X_19	1	0.201064	0.365520
samw_F_L_oly_4X_20	1	0.210696	0.386040
samw_F_L_oly_4X_21	1	0.181120	0.342652
samw_F_L_oly_4X_22	1	0.191472	0.350928

1-10 of 49 rows | 1-4 of 95 columns Previous **1** 2 3 4 5 Next

Create a second comparison dataframe.

```
first_group = melt(join_3) %>% dplyr::filter(variable=="Total.wing.area.run1") %>% mutate(variab
le=1)
second_group = melt(join_3) %>% dplyr::filter(variable=="Total.wing.area.run2") %>% mutate(varia
ble=2)
third_group = melt(join_3) %>% dplyr::filter(variable=="Total.wing.area") %>% mutate(variable=3)
fourth_group = melt(join_3) %>% dplyr::filter(variable=="mTA") %>% mutate(variable=4)


comparison_df_2 = bind_rows(first_group, second_group, third_group, fourth_group)
comparison_df_2
```

Wing.name <chr>	variable <dbl>	value <dbl>
samw_F_L_oly_4X_12	1	2.406632
samw_F_L_oly_4X_13	1	2.380364
samw_F_L_oly_4X_14	1	2.272792
samw_F_L_oly_4X_16	1	2.309568
samw_F_L_oly_4X_17	1	2.165168
samw_F_L_oly_4X_18	1	2.351600
samw_F_L_oly_4X_19	1	2.419604
samw_F_L_oly_4X_20	1	2.515296

Wing.name <chr>	variable <dbl>	value <dbl>
samw_F_L_oly_4X_21	1	2.209732
samw_F_L_oly_4X_22	1	2.334128

1-10 of 196 rows Previous **1** 2 3 4 5 6 ... 20 Next

Check normality assumptions for the complete combined data

Each dataset does not differ significantly from a normal distribution via Shaprio-Wilk's test ($p < 0.05$). Therefore, we can proceed with Bartlett's test and a one-way ANOVA test.

NOTE: The data for the indpendent MAPPER runs had sample sizes of 61 wings each. The manual measurements to compare to only had a sample size of 49 wings. The second comparison dataframe has a sample size of 49 wings for each independent MAPPER run and manual measurement to be able to match MAPPER's predicted output to manual measurements for root-mean-square error calculations.

```
testSelection = comparison_df_2 %>% dplyr::filter(variable==1)
shapiro.test(testSelection$value)
```

```
    Shapiro-Wilk normality test

data:  testSelection$value
W = 0.96514, p-value = 0.1541
```

```
testSelection = comparison_df_2 %>% dplyr::filter(variable==2)
shapiro.test(testSelection$value)
```

```
    Shapiro-Wilk normality test

data:  testSelection$value
W = 0.96835, p-value = 0.208
```

```
testSelection = comparison_df_2 %>% dplyr::filter(variable==3)
shapiro.test(testSelection$value)
```

```
    Shapiro-Wilk normality test

data:  testSelection$value
W = 0.95865, p-value = 0.08337
```

```
testSelection = comparison_df_2 %>% dplyr::filter(variable==4)
shapiro.test(testSelection$value)
```

```
	Shapiro-Wilk normality test

data:  testSelection$value
W = 0.96453, p-value = 0.1455
```

Bartlett's test

In order to assess homogeneity of variances, we will perform Bartlett's test. More information can be found Here (http://www.sthda.com/english/wiki/compare-multiple-sample-variances-in-r). There is no statistical differences in variances of the independent MAPPER runs and manual measurements.

```
bartlett.test(value~factor(variable), data=comparison_df_2)
```

```
	Bartlett test of homogeneity of variances

data:  value by factor(variable)
Bartlett's K-squared = 0.15332, df = 3, p-value = 0.9847
```

ANOVA

In order to assess equlity of means, we will perform a one-way ANOVA test. More information can be found Here (http://www.sthda.com/english/wiki/one-way-anova-test-in-r). There is no statistical differences in the means of the independent MAPPER runs and manual measurements.

```
aov(value~factor(variable), data=comparison_df_2) %>% summary()
```

```
                  Df Sum Sq Mean Sq F value Pr(>F)
factor(variable)   3  0.062 0.02080   1.161  0.326
Residuals        192  3.441 0.01792
```

Violin plot of MAPPER and manual independent runs

Plotted is the distribution of total wing area done manually and compared to MAPPER's automated output for all independent runs of MAPPER. Blue line indicates the mean with the red lines being the 95% CI of the mean.

```
#svg("MAPPERValidationPlots/RMSEviolin.svg", family = "arial", width=3, height=2)
ggplot(melt(join_3) %>% dplyr::filter(variable=="Total.wing.area.run1" | variable == "Total.win
g.area.run2" | variable == "Total.wing.area" | variable =="mTA"),
       aes(x=factor(variable),
           y=value)) +
  geom_violin(scale="width", alpha=0.50, fill="lightslateblue") +
  geom_jitter(width=0.25) +
  #stat_summary(fun="median", geom="crossbar", size=0.5, fill="blue", color = "blue") +
  #stat_summary(fun="cimed", geom="crossbar", size=0.5, fill="red", color = "red") +
  stat_summary(fun="mean", geom="crossbar", size=0.5, fill="blue", color = "blue") +
  stat_summary(fun="cimean", geom="crossbar", size=0.5, fill="red", color = "red") +
  theme(axis.text.x = element_text(angle = 45, vjust = 0.6),
        axis.title.x=element_blank()) +
  labs(x=expression(paste("")), y=expression(paste("Total Wing Area"))) +
  theme(panel.grid.major = element_blank(), panel.grid.minor = element_blank(),
panel.background = element_blank(), axis.line = element_line(colour = "black"))
```

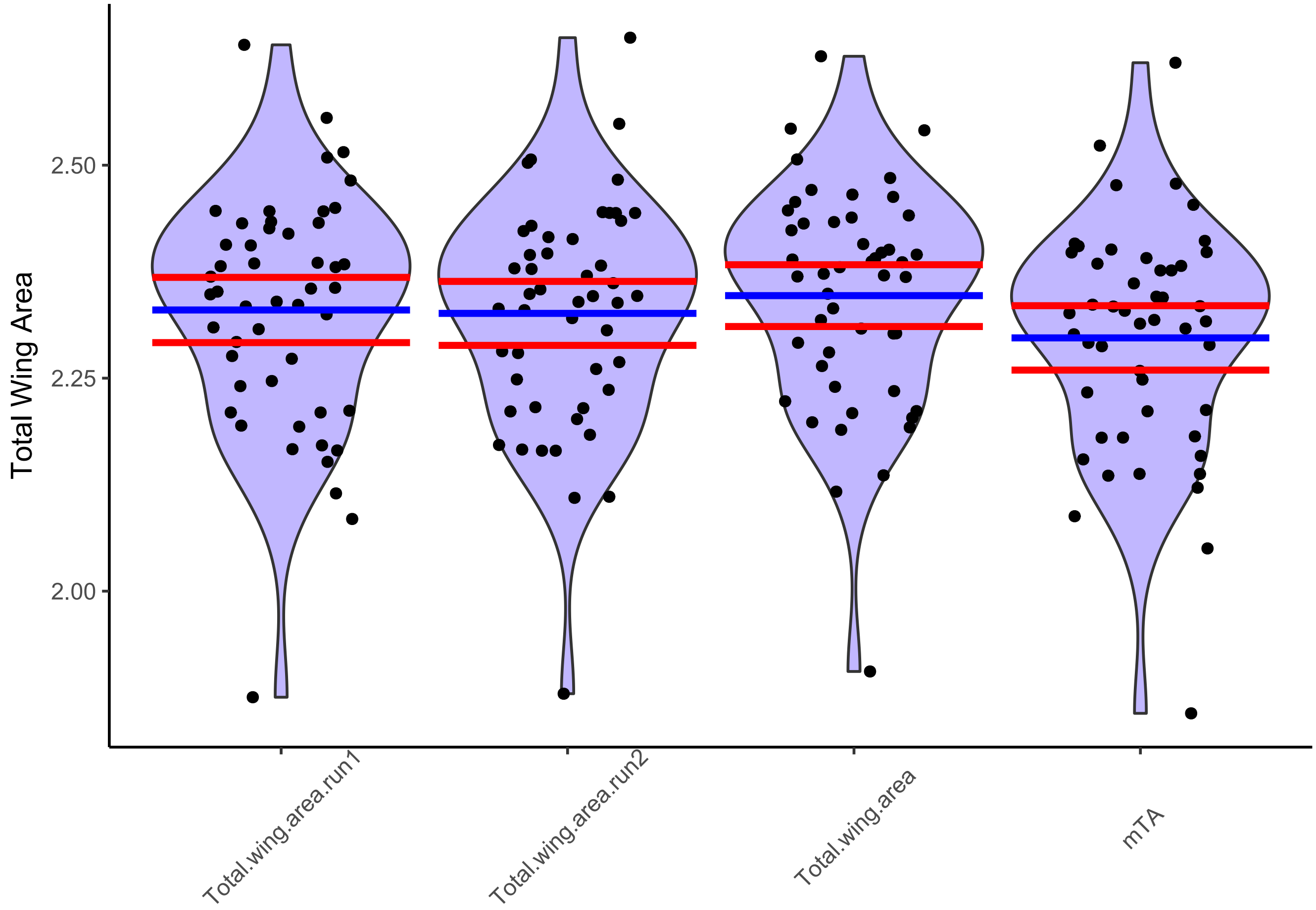

Root-mean-square error (RMSE) evaluations

Extract the values from each independent run to compare to manual measurements.

```
manual_vals = fourth_group$value
run_one_vals = first_group$value
run_two_vals = second_group$value
run_three_vals = third_group$value
```

Run one prediction metrics

```
rmserr(manual_vals, run_one_vals, summary=TRUE)
```

```
-- Error Terms ---------------------------------------------------
 MAE:   0.0327      - mean absolute error (in range [ 1.856576 2.620272 ])
 MSE:   0.0012      - mean squared error (the variance?!)
 RMSE:  0.0339      - root mean squared error (std. dev.)
 MAPE:  0.0143      - mean absolute percentage error
 LMSE:  0.0645      - normalized mean squared error
 rSTD:  0.0148      - relative standard deviation ( 2.297286 )
-----------------------------------------------------------------
```

Run two prediction metrics

```
rmserr(manual_vals, run_two_vals, summary=TRUE)
```

```
-- Error Terms ---------------------------------------------------
 MAE:   0.0291      - mean absolute error (in range [ 1.856576 2.620272 ])
 MSE:   0.0010      - mean squared error (the variance?!)
 RMSE:  0.0313      - root mean squared error (std. dev.)
 MAPE:  0.0127      - mean absolute percentage error
 LMSE:  0.0547      - normalized mean squared error
 rSTD:  0.0136      - relative standard deviation ( 2.297286 )
-----------------------------------------------------------------
```

Run three prediction metrics

```
rmserr(manual_vals, run_three_vals, summary=TRUE)
```

```
-- Error Terms ---------------------------------------------------
 MAE:   0.0496      - mean absolute error (in range [ 1.856576 2.620272 ])
 MSE:   0.0028      - mean squared error (the variance?!)
 RMSE:  0.0530      - root mean squared error (std. dev.)
 MAPE:  0.0218      - mean absolute percentage error
 LMSE:  0.1571      - normalized mean squared error
 rSTD:  0.0231      - relative standard deviation ( 2.297286 )
-----------------------------------------------------------------
```

Normalized root-mean-square error (NRMSE)

To better assess how well the independent runs performed compared to manual measurements, the RMSE value of each independent MAPPER run was normalized to the mean value ($\bar{y}$) of the manual measurements to obtain:

$$\mathrm{NRMSE} = \frac{\mathrm{RMSE}}{\bar{y}} \qquad \mathrm{RMSE} = \sqrt{\frac{\sum_{i=1}^{N}(x_i - \hat{x}_i)^2}{N}}$$

This is similar to the form of the coefficient of variation (CV) of a dataset, wherein the CV is the standard deviation scaled to the mean of the dataset:

$$\mathrm{CV} = \frac{\sigma}{\mu} \qquad \sigma = \sqrt{\frac{\sum_{i=1}^{N}(x_i - \bar{x}_i)^2}{N}}$$

Because RMSE is an estimator for the standard deviation of the distribution of the MAPPER predicted residuals, benchmarking MAPPER NRMSEs to the manual measurement CV will provide insight into how well MAPPER performed in measuring the wing areas.

Run one NRMSE

```
(100 * sqrt(sum((run_one_vals-manual_vals)^2)/length(manual_vals)) / mean(manual_vals)) %>% roun
d(2)
```

```
[1] 1.48
```

Run two NRMSE

```
(100 * sqrt(sum((run_two_vals-manual_vals)^2)/length(manual_vals)) / mean(manual_vals)) %>% roun
d(2)
```

```
[1] 1.36
```

Run three NRMSE

```
(100 * sqrt(sum((run_three_vals-manual_vals)^2)/length(manual_vals)) / mean(manual_vals)) %>% ro
und(2)
```

```
[1] 2.31
```

Coefficient of varation

```
100* std(manual_vals) / mean(manual_vals)
```

```
[1] 5.875931
```

Mean absolute percentage error (MAPE)

Another metric to evaulate how well MAPPER performed is the mean absolute percentage error. This metric measures the size of the prediction error in percentage form.

Run one MAPE

```
(100 * sum(abs((run_one_vals-manual_vals)/manual_vals))/length(manual_vals)) %>% round(2)
```

```
[1] 1.43
```

Run two MAPE

```
(100 * sum(abs((run_two_vals-manual_vals)/manual_vals))/length(manual_vals)) %>% round(2)
```

```
[1] 1.27
```

Run three MAPE

```
(100 * sum(abs((run_three_vals-manual_vals)/manual_vals))/length(manual_vals)) %>% round(2)
```

```
[1] 2.18
```

Insulin signaling and FIJI wings comparisons

Load datasets

```
FIJI_area_df = read.csv("MAPPER_FIJI_Area.csv", header=T, sep=",") %>% tibble()
FIJI_trichome_df = read.csv("MAPPER_FIJI_Trichome.csv", header=T, sep=",") %>% tibble()
```

```
FIJI_area_df
```

ï..Filename <chr>	Genotype <int>	MAPPER.area <dbl>	FIJI.area <dbl>
'1346 x 1194 Sample 1'	1	1.1444723	1.1324279
'1346 x 1194 Sample 10'	1	1.1794971	1.1775789
'1346 x 1194 Sample 11'	1	1.1172226	1.1119504
'1346 x 1194 Sample 12'	1	1.1790965	1.1754063
'1346 x 1194 Sample 13'	1	1.1427500	1.1356327
'1346 x 1194 Sample 14'	1	1.1597682	1.1614525
'1346 x 1194 Sample 15'	1	1.1052601	1.1038361
'1346 x 1194 Sample 16'	1	1.1200736	1.1163161

ï..Filename<br><chr>	Genotype<br><int>	MAPPER.area<br><dbl>	FIJI.area<br><dbl>
'1346 x 1194 Sample 17'	1	1.1341005	1.1329045
'1346 x 1194 Sample 2'	1	1.0912069	1.0982276

1-10 of 47 rows Previous **1** 2 3 4 5 Next

```
FIJI_trichome_df
```

ï..Filename<br><chr>	Genotype<br><int>	MAPPER.Trichome<br><int>	FIJI.Trichome<br><int>
'1346 x 1194 Sample 1.tif'	1	168	238
'1346 x 1194 Sample 10.tif'	1	154	234
'1346 x 1194 Sample 11.tif'	1	163	242
'1346 x 1194 Sample 12.tif'	1	151	211
'1346 x 1194 Sample 13.tif'	1	146	223
'1346 x 1194 Sample 14.tif'	1	160	207
'1346 x 1194 Sample 15.tif'	1	161	239
'1346 x 1194 Sample 16.tif'	1	162	197
'1346 x 1194 Sample 17.tif'	1	147	217
'1346 x 1194 Sample 2.tif'	1	143	204

1-10 of 47 rows Previous **1** 2 3 4 5 Next

Statistical comparisons for area

Create separate groups for RyR-RNAi, InsR-CA, and InsR-DN.

```
Ryr_group = FIJI_area_df %>% dplyr::filter(Genotype==1)
InsRCA_group = FIJI_area_df %>% dplyr::filter(Genotype==2)
InsRDN_group = FIJI_area_df %>% dplyr::filter(Genotype==3)
```

Compare MAPPER to FIJI for RyR-RNAi with unpaired T-Test and F-test

There are no statistical differences in wing area for RyR-RNAi comparing MAPPER to FIJI.

```
canUseUnpairedTTest(Ryr_group$MAPPER.area, Ryr_group$FIJI.area)
```

```
[1] "Unpaired T-test is viable"
```

```
	Two Sample t-test

data:  df1 and df2
t = 0.14396, df = 32, p-value = 0.8864
alternative hypothesis: true difference in means is not equal to 0
95 percent confidence interval:
 -0.02038289  0.02348313
sample estimates:
mean of x mean of y 
 1.132098  1.130548 
```

```
var.test(Ryr_group$MAPPER.area, Ryr_group$FIJI.area)
```

```
	F test to compare two variances

data:  Ryr_group$MAPPER.area and Ryr_group$FIJI.area
F = 1.0891, num df = 16, denom df = 16, p-value = 0.8665
alternative hypothesis: true ratio of variances is not equal to 1
95 percent confidence interval:
 0.3944114 3.0074277
sample estimates:
ratio of variances 
          1.089111 
```

Compare MAPPER to FIJI for InsR-CA with unpaired T-Test and F-test

There are no statistical differences in wing area for InsR-CA comparing MAPPER to FIJI.

```
canUseUnpairedTTest(InsRCA_group$MAPPER.area, InsRCA_group$FIJI.area)
```

```
[1] "Unpaired T-test is viable"
```

```
	Two Sample t-test

data:  df1 and df2
t = 0.5005, df = 48, p-value = 0.619
alternative hypothesis: true difference in means is not equal to 0
95 percent confidence interval:
 -0.01617868  0.02690288
sample estimates:
mean of x mean of y 
 1.462169  1.456807 
```

```
var.test(InsRCA_group$MAPPER.area, InsRCA_group$FIJI.area)
```

```

    F test to compare two variances

data:  InsRCA_group$MAPPER.area and InsRCA_group$FIJI.area
F = 1.0192, num df = 24, denom df = 24, p-value = 0.9633
alternative hypothesis: true ratio of variances is not equal to 1
95 percent confidence interval:
 0.4491126 2.3127588
sample estimates:
ratio of variances
          1.019161
```

Compare MAPPER to FIJI for InsR-CA with unpaired T-Test and F-test

There are no statistical differences in wing area for InsR-DN comparing MAPPER to FIJI.

```
canUseUnpairedTTest(InsRDN_group$MAPPER.area, InsRDN_group$FIJI.area)
```

```
[1] "Unpaired T-test is viable"
```

```

    Two Sample t-test

data:  df1 and df2
t = 0.18868, df = 8, p-value = 0.855
alternative hypothesis: true difference in means is not equal to 0
95 percent confidence interval:
 -0.01923573  0.02266395
sample estimates:
mean of x mean of y
0.6557265 0.6540124
```

```
var.test(InsRDN_group$MAPPER.area, InsRDN_group$FIJI.area)
```

```
	F test to compare two variances

data:  InsRDN_group$MAPPER.area and InsRDN_group$FIJI.area
F = 0.88595, num df = 4, denom df = 4, p-value = 0.9094
alternative hypothesis: true ratio of variances is not equal to 1
95 percent confidence interval:
 0.09224254 8.50909665
sample estimates:
ratio of variances
         0.8859462
```

Generate violin plots

RyR-RNAi area violin plot

Plotted is the distribution of the total wing area for MAPPER compared to FIJI. Blue line indicates the mean with the red lines being the 95% CI of the mean.

```
#svg("MAPPERValidationPlots/FIJI_RyR_Violin.svg", family = "arial", width=3, height=2)

ggplot(melt(Ryr_group) %>% dplyr::filter(variable=="MAPPER.area" | variable == "FIJI.area"),
       aes(x=factor(variable),
           y=value)) +
  geom_violin(scale="width", alpha=0.50, fill="lightslateblue") +
  geom_jitter(width=0.25) +
  #stat_summary(fun="median", geom="crossbar", size=0.5, fill="blue", color = "blue") +
  #stat_summary(fun="cimed", geom="crossbar", size=0.5, fill="red", color = "red") +
  stat_summary(fun="mean", geom="crossbar", size=0.5, fill="blue", color = "blue") +
  stat_summary(fun="cimean", geom="crossbar", size=0.5, fill="red", color = "red") +
  theme(axis.text.x = element_text(angle = 45, vjust = 0.6),
        axis.title.y=element_blank()) +
  labs(x=expression(paste(""))) +
  theme(panel.grid.major = element_blank(), panel.grid.minor = element_blank(),
panel.background = element_blank(), axis.line = element_line(colour = "black"))
```

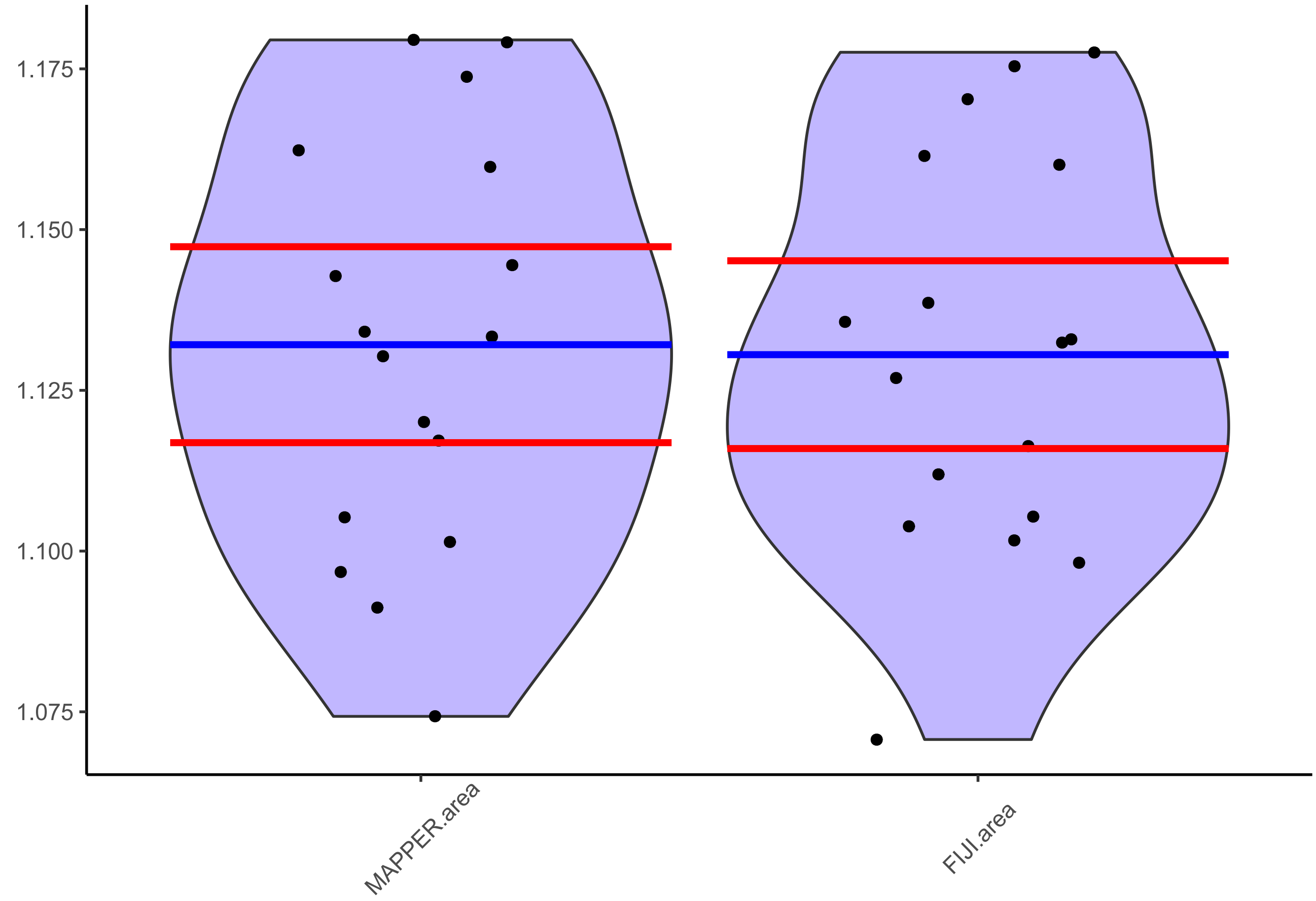

InsR-CA area violin plot

Plotted is the distribution of the total wing area for MAPPER compared to FIJI. Blue line indicates the mean with the red lines being the 95% CI of the mean.

```
#svg("MAPPERValidationPlots/FIJI_InsRCA_Violin.svg", family = "arial", width=3, height=2)

ggplot(melt(InsRCA_group) %>% dplyr::filter(variable=="MAPPER.area" | variable == "FIJI.area"),
       aes(x=factor(variable),
           y=value)) +
  geom_violin(scale="width", alpha=0.50, fill="lightslateblue") +
  geom_jitter(width=0.25) +
  #stat_summary(fun="median", geom="crossbar", size=0.5, fill="blue", color = "blue") +
  #stat_summary(fun="cimed", geom="crossbar", size=0.5, fill="red", color = "red") +
  stat_summary(fun="mean", geom="crossbar", size=0.5, fill="blue", color = "blue") +
  stat_summary(fun="cimean", geom="crossbar", size=0.5, fill="red", color = "red") +
  theme(axis.text.x = element_text(angle = 45, vjust = 0.6),
        axis.title.y=element_blank()) +
  labs(x=expression(paste(""))) +
  theme(panel.grid.major = element_blank(), panel.grid.minor = element_blank(),
panel.background = element_blank(), axis.line = element_line(colour = "black"))
```

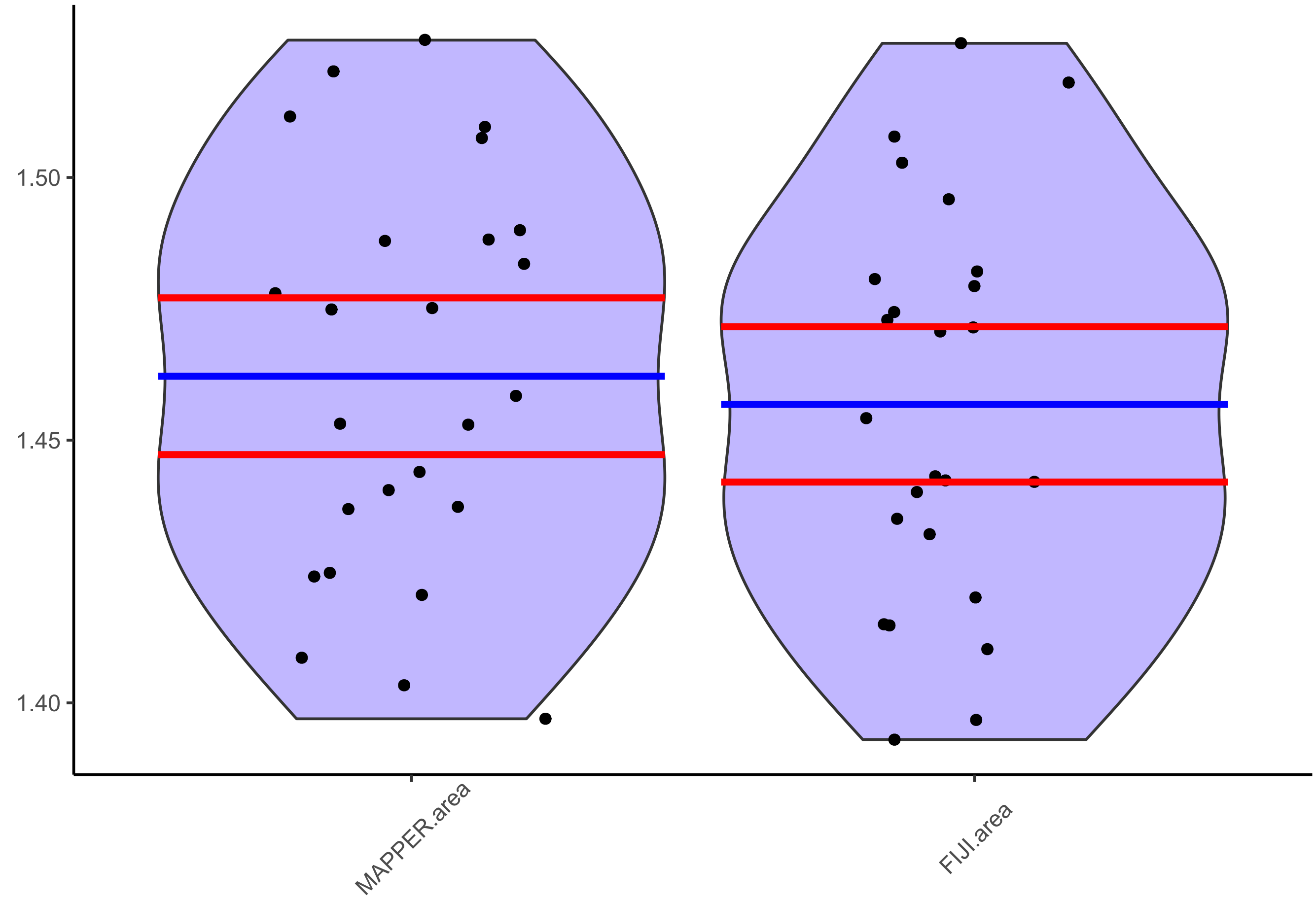

InsR-DN area violin plot

Plotted is the distribution of the total wing area for MAPPER compared to FIJI. Blue line indicates the mean with the red lines being the 95% CI of the mean.

```
#svg("MAPPERValidationPlots/FIJI_InsRDN_violin.svg", family = "arial", width=3, height=2)

ggplot(melt(InsRDN_group) %>% dplyr::filter(variable=="MAPPER.area" | variable == "FIJI.area"),
       aes(x=factor(variable),
           y=value)) +
  geom_violin(scale="width", alpha=0.50, fill="lightslateblue") +
  geom_jitter(width=0.25) +
  #stat_summary(fun="median", geom="crossbar", size=0.5, fill="blue", color = "blue") +
  #stat_summary(fun="cimed", geom="crossbar", size=0.5, fill="red", color = "red") +
  stat_summary(fun="mean", geom="crossbar", size=0.5, fill="blue", color = "blue") +
  stat_summary(fun="cimean", geom="crossbar", size=0.5, fill="red", color = "red") +
  theme(axis.text.x = element_text(angle = 45, vjust = 0.6),
        axis.title.y=element_blank()) +
  labs(x=expression(paste(""))) +
  theme(panel.grid.major = element_blank(), panel.grid.minor = element_blank(),
panel.background = element_blank(), axis.line = element_line(colour = "black"))
```

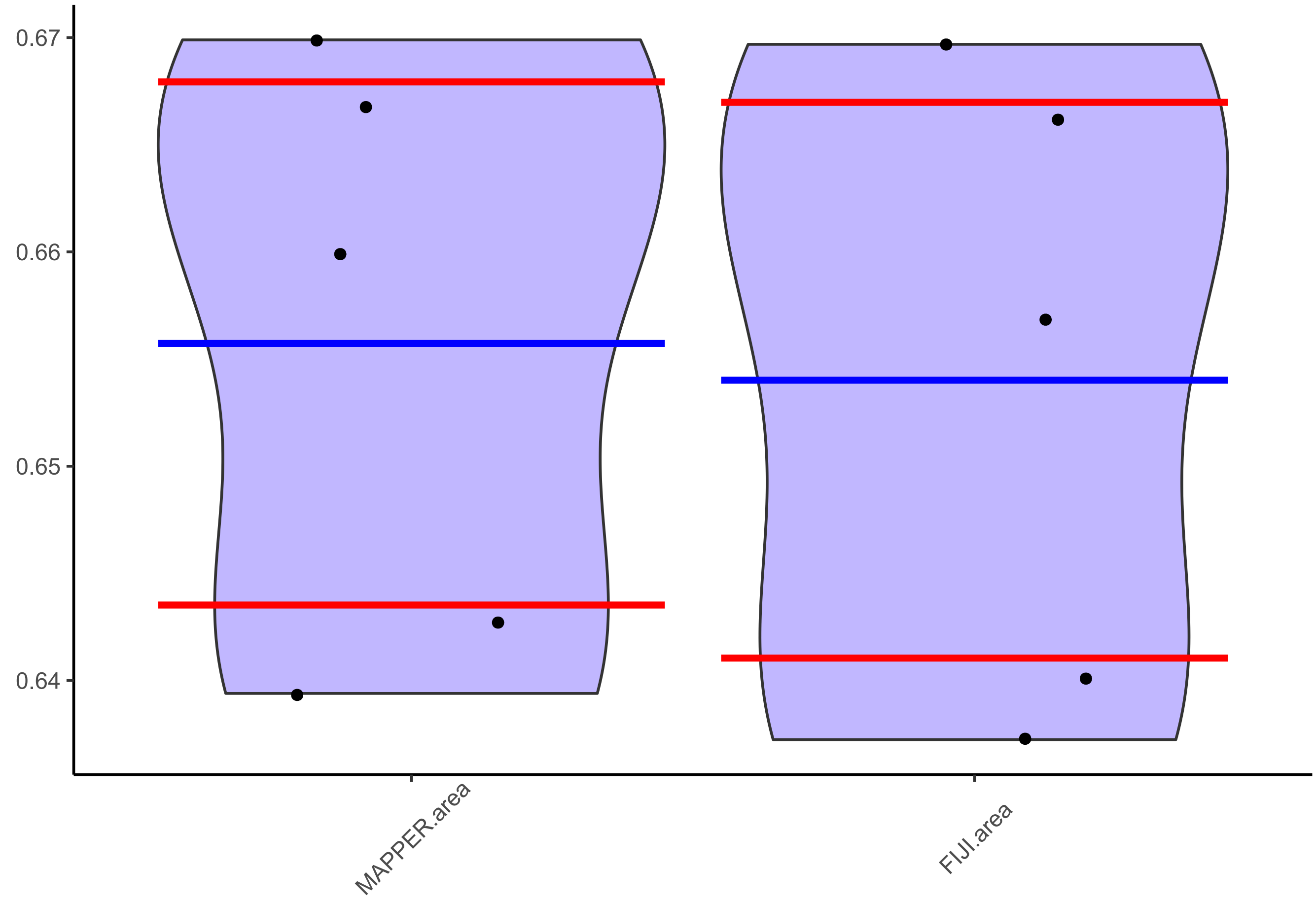

Statistical comparisons for trichome count

Create separate groups for RyR-RNAi, InsR-CA, and InsR-DN.

```
Ryr_group_trichome = FIJI_trichome_df %>% dplyr::filter(Genotype==1)
InsRCA_group_trichome = FIJI_trichome_df %>% dplyr::filter(Genotype==2)
InsRDN_group_trichome = FIJI_trichome_df %>% dplyr::filter(Genotype==3)
```

Compare MAPPER to FIJI for RyR-RNAi with Mann-Whitney U Test

FIJIWings significantly overestimates trichome counts compared to MAPPER for RyR-RNAi.

```
wilcox.test(Ryr_group_trichome$MAPPER.Trichome, Ryr_group_trichome$FIJI.Trichome,
                      correct = FALSE,
                      alternative = "two.sided", paired = FALSE, exact=TRUE, conf.level = 1-alph
a)
```

```
    Wilcoxon rank sum test

data:  Ryr_group_trichome$MAPPER.Trichome and Ryr_group_trichome$FIJI.Trichome
W = 7, p-value = 2.145e-06
alternative hypothesis: true location shift is not equal to 0
```

Compare MAPPER to FIJI for InsR-CA with Mann-Whitney U Test

FIJIWings significantly overestimates trichome counts compared to MAPPER for InsR-CA.

```
wilcox.test(InsRCA_group_trichome$MAPPER.Trichome, InsRCA_group_trichome$FIJI.Trichome,
                      correct = FALSE,
                      alternative = "two.sided", paired = FALSE, exact=TRUE, conf.level = 1-alph
a)
```

```
    Wilcoxon rank sum test

data:  InsRCA_group_trichome$MAPPER.Trichome and InsRCA_group_trichome$FIJI.Trichome
W = 78.5, p-value = 5.589e-06
alternative hypothesis: true location shift is not equal to 0
```

Compare MAPPER to FIJI for InsR-DN with Mann-Whitney U Test

FIJIWings significantly overestimates trichome counts compared to MAPPER for InsR-DN.

```
wilcox.test(InsRDN_group_trichome$MAPPER.Trichome, InsRDN_group_trichome$FIJI.Trichome,
                      correct = FALSE,
                      alternative = "two.sided", paired = FALSE, exact=TRUE, conf.level = 1-alph
a)
```

```
    Wilcoxon rank sum exact test

data:  InsRDN_group_trichome$MAPPER.Trichome and InsRDN_group_trichome$FIJI.Trichome
W = 0, p-value = 0.007937
alternative hypothesis: true location shift is not equal to 0
```

Generate violin plots

RyR-RNAi trichome violin plot

Plotted is the distribution of the total trichome counts for MAPPER compared to FIJIWings. Blue line indicates the median with the red lines being the 95% CI of the median.

```
#svg("MAPPERValidationPlots/RyR_Trichome_FIJI.svg", family = "arial", width=3, height=2)
ggplot(melt(Ryr_group_trichome) %>% dplyr::filter(variable=="MAPPER.Trichome" | variable == "FIJ
I.Trichome"),
       aes(x=factor(variable),
           y=value)) +
  geom_violin(scale="width", alpha=0.50, fill="lightslateblue") +
  geom_jitter(width=0.25) +
  stat_summary(fun="median", geom="crossbar", size=0.5, fill="blue", color = "blue") +
  stat_summary(fun="cimed", geom="crossbar", size=0.5, fill="red", color = "red") +
  #stat_summary(fun="mean", geom="crossbar", size=0.5, fill="blue", color = "blue") +
  #stat_summary(fun="cimean", geom="crossbar", size=0.5, fill="red", color = "red") +
  theme(axis.text.x = element_text(angle = 45, vjust = 0.6),
        axis.title.x=element_blank()) +
  labs(x=expression(paste("")), y=expression(paste("75 x 75 pixel area trichome count"))) +
  theme(panel.grid.major = element_blank(), panel.grid.minor = element_blank(),
panel.background = element_blank(), axis.line = element_line(colour = "black"))
```

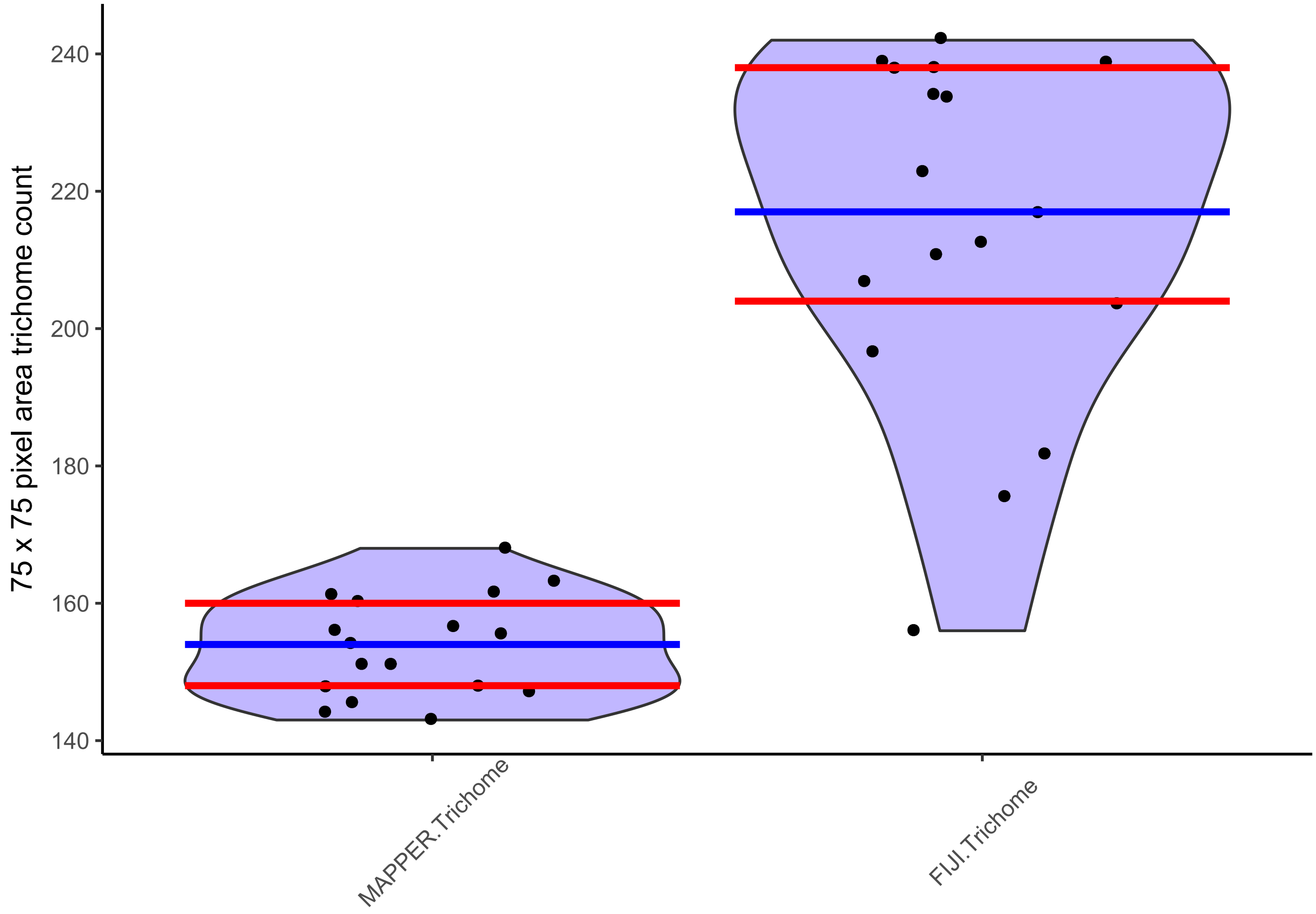

InsR-CA trichome violin plot

Plotted is the distribution of the total trichome counts for MAPPER compared to FIJIWings. Blue line indicates the median with the red lines being the 95% CI of the median.

```
#svg("MAPPERValidationPlots/InsRCA_Trichome_FIJI.svg", family = "arial", width=3, height=2)
ggplot(melt(InsRCA_group_trichome) %>% dplyr::filter(variable=="MAPPER.Trichome" | variable ==
"FIJI.Trichome"),
       aes(x=factor(variable),
           y=value)) +
  geom_violin(scale="width", alpha=0.50, fill="lightslateblue") +
  geom_jitter(width=0.25) +
  stat_summary(fun="median", geom="crossbar", size=0.5, fill="blue", color = "blue") +
  stat_summary(fun="cimed", geom="crossbar", size=0.5, fill="red", color = "red") +
  #stat_summary(fun="mean", geom="crossbar", size=0.5, fill="blue", color = "blue") +
  #stat_summary(fun="cimean", geom="crossbar", size=0.5, fill="red", color = "red") +
  theme(axis.text.x = element_text(angle = 45, vjust = 0.6),
        axis.title.x=element_blank()) +
  labs(x=expression(paste("")), y=expression(paste("75 x 75 pixel area trichome count"))) +
  theme(panel.grid.major = element_blank(), panel.grid.minor = element_blank(),
panel.background = element_blank(), axis.line = element_line(colour = "black"))
```

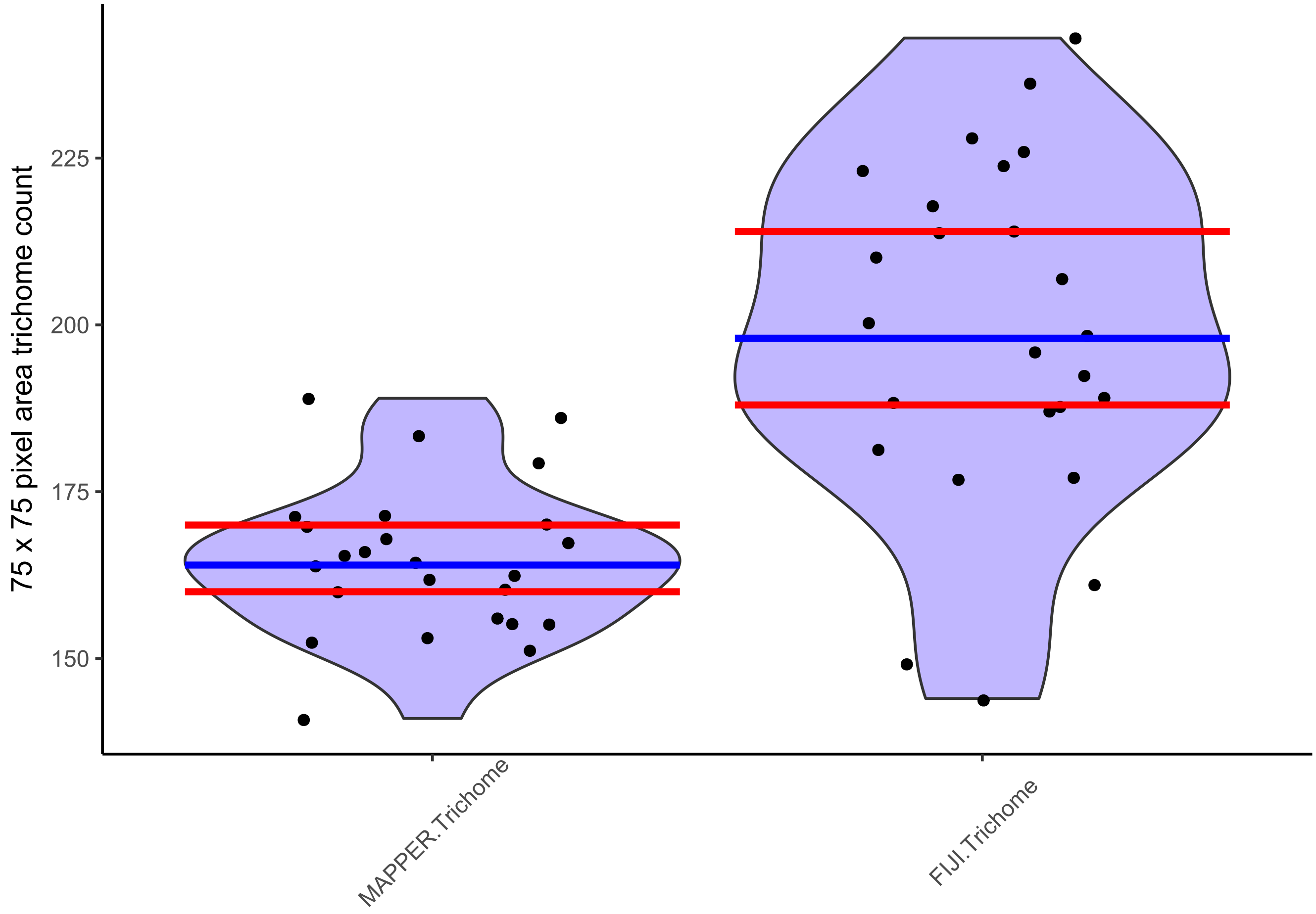

InsR-DN trichome violin plot

Plotted is the distribution of the total trichome counts for MAPPER compared to FIJIWings. Blue line indicates the median.

NOTE: The sample sizes are too small to create confidence intervals of the median for each group.

```
#svg("MAPPERValidationPlots/InsRDN_Trichome_FIJI.svg", family = "arial", width=3, height=2)
ggplot(melt(InsRDN_group_trichome) %>% dplyr::filter(variable=="MAPPER.Trichome" | variable ==
"FIJI.Trichome"),
       aes(x=factor(variable),
           y=value)) +
  geom_violin(scale="width", alpha=0.50, fill="lightslateblue") +
  geom_jitter(width=0.25) +
  stat_summary(fun="median", geom="crossbar", size=0.5, fill="blue", color = "blue") +
  stat_summary(fun="cimed", geom="crossbar", size=0.5, fill="red", color = "red") +
  #stat_summary(fun="mean", geom="crossbar", size=0.5, fill="blue", color = "blue") +
  #stat_summary(fun="cimean", geom="crossbar", size=0.5, fill="red", color = "red") +
  theme(axis.text.x = element_text(angle = 45, vjust = 0.6),
        axis.title.x=element_blank()) +
  labs(x=expression(paste("")), y=expression(paste("75 x 75 pixel area trichome count"))) +
  theme(panel.grid.major = element_blank(), panel.grid.minor = element_blank(),
panel.background = element_blank(), axis.line = element_line(colour = "black"))
```

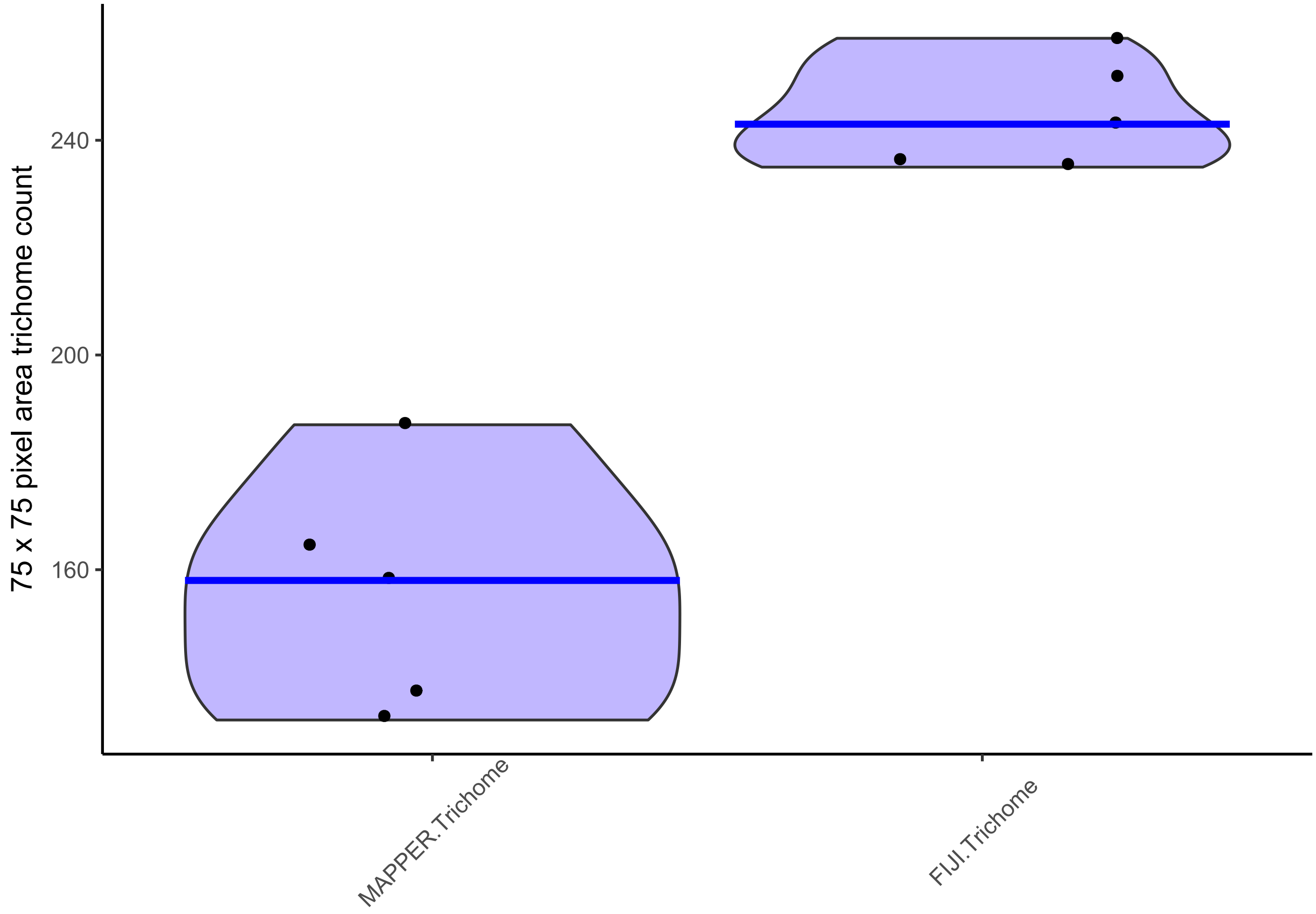

Instead of plotting the medians, the mean is plotted. Comparisons should be taken with much consideration as the sample sizes are small and the distributions are for discrete count data.

```
#svg("MAPPERValidationPlots/InsRDN_Trichome_FIJI_Mean.svg", family = "arial", width=3, height=2)
ggplot(melt(InsRDN_group_trichome) %>% dplyr::filter(variable=="MAPPER.Trichome" | variable ==
"FIJI.Trichome"),
       aes(x=factor(variable),
           y=value)) +
  geom_violin(scale="width", alpha=0.50, fill="lightslateblue") +
  geom_jitter(width=0.25) +
  #stat_summary(fun="median", geom="crossbar", size=0.5, fill="blue", color = "blue") +
  #stat_summary(fun="cimed", geom="crossbar", size=0.5, fill="red", color = "red") +
  stat_summary(fun="mean", geom="crossbar", size=0.5, fill="blue", color = "blue") +
  stat_summary(fun="cimean", geom="crossbar", size=0.5, fill="red", color = "red") +
  theme(axis.text.x = element_text(angle = 45, vjust = 0.6),
        axis.title.x=element_blank()) +
  labs(x=expression(paste("")), y=expression(paste("75 x 75 pixel area trichome count"))) +
  theme(panel.grid.major = element_blank(), panel.grid.minor = element_blank(),
panel.background = element_blank(), axis.line = element_line(colour = "black"))
```

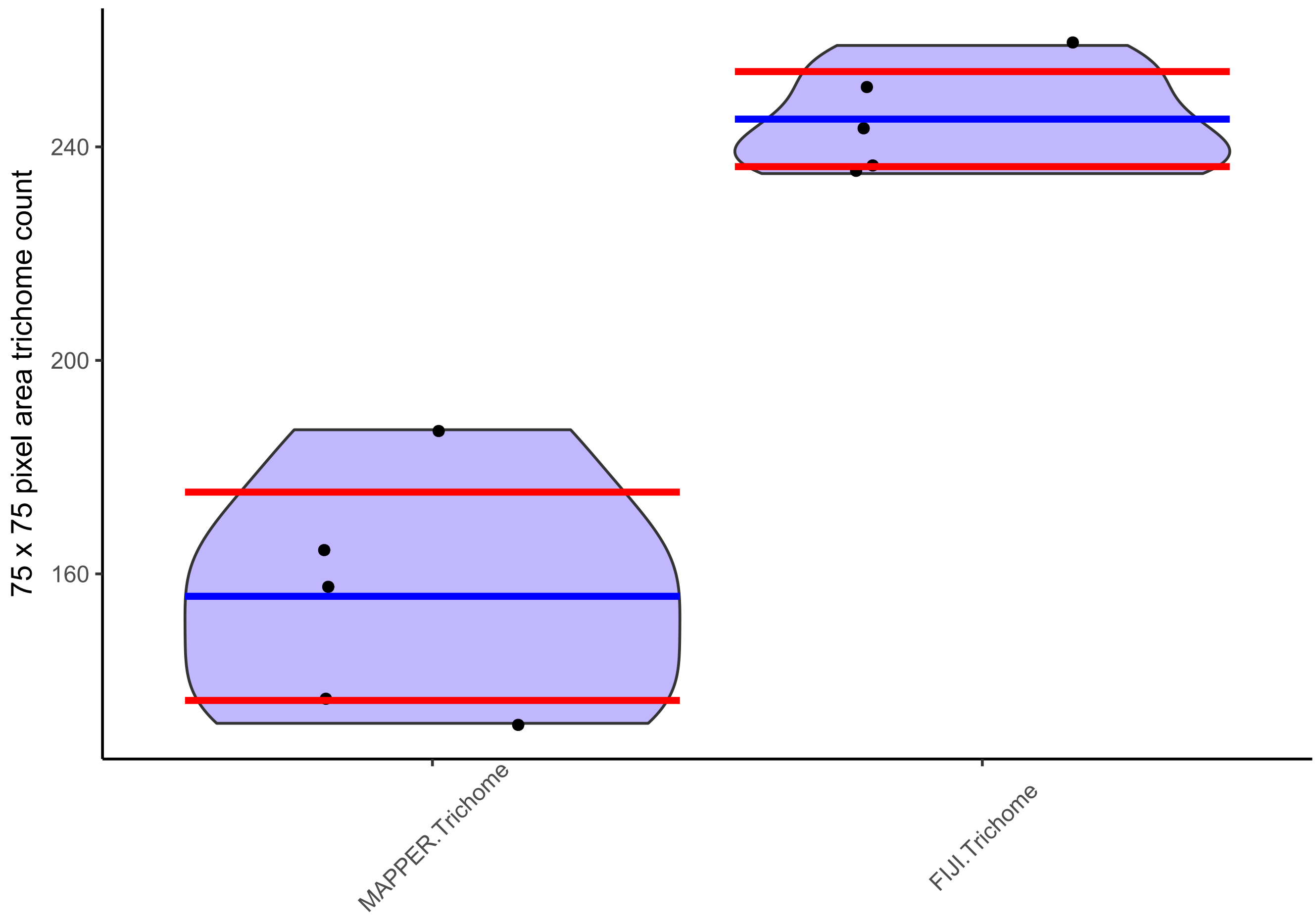

Supplement: Supplementary file 19 [file Presentation2.pdf]
